# A machine learning integrated multi-omics framework for risk prediction and target discovery in insomnia aggravated sepsis induced acute lung injury

**DOI:** 10.3389/fimmu.2026.1721749

**Published:** 2026-06-01

**Authors:** Jinquan Zhang, Yuwei Zhang, Zeyu Liu, Xiaona Chen, Zhengzheng Yan, Zhixia Chen, Quan Li

**Affiliations:** 1 Guangzhou University of Chinese Medicine, Guangzhou, China; 2 Department of Anesthesiology, National Cancer Center/National Clinical Research Center for Cancer/Cancer Hospital and Shenzhen Hospital, Chinese Academy of Medical Sciences and Peking Union Medical College, Shenzhen, China; 3 Shenzhen Clinical Medical College, Guangzhou University of Chinese Medicine, Shenzhen, China; 4 Department of Anesthesiology, Renji Hospital, Shanghai Jiao Tong University School of Medicine, Key Laboratory of Anesthesiology (Shanghai Jiao Tong University), Ministry of Education, Shanghai, China; 5 Department of Anesthesiology, The Third Affiliated Hospital of Anhui Medical University, Hefei, Anhui, China; 6 School of Medicine, Southern University of Science and Technology, Shenzhen, Guangdong, China; 7 Department of Anesthesiology, Dongguan Key Laboratory of Anesthesia and Organ Protection, The Tenth Affiliated Hospital of Southern Medical University, Dongguan, Guangdong, China

**Keywords:** biomarkers, insomnia, machine learning, PTPN6, sepsis induced acute lung injury (SALI)

## Abstract

**Objective:**

This study aims to identify critical biomarkers and clarify how insomnia exacerbates sepsis-induced acute lung injury (SALI). We used integrative multi-omics approaches and machine learning.

**Methods:**

A causal association between sepsis and insomnia was established using Mendelian randomization (MR). We used weighted gene co-expression network analysis (WGCNA) to identify genes linked to both insomnia and SALI. We used machine learning techniques (Random Forest, SVM, KNN) with SHAP interpretability modeling to refine gene signatures. The diagnostic and prognostic value of these genes was investigated. To elucidate the underlying molecular pathways, functional enrichment analyses, including KEGG, GO, PPI, and GSEA were performed. To validate gene expression patterns and cellular localization, transcriptomic profiling, single-cell RNA sequencing (scRNA-seq), and *in vivo* and vitro experimental validation were employed.

**Results:**

MR analysis identified insomnia as a causal determinant in susceptibility to sepsis. Complementary pathological evidence from preclinical sleep deprivation models further confirmed its role in exacerbating progression of SALI. The WGCNA revealed 1,294 co-dysregulated genes shared between insomnia and SALI. These genes were significantly enriched in biological processes, including immune regulation and phagocytic vesicle formation, as well as KEGG pathways such as tuberculosis infection and chemokine signaling. Among these,102 genes exhibited differential expression in a murine SALI model induced by LPS. Through machine learning analysis, ISG20, MYO1F, and PTPN6 were identified as robust hub genes. Further diagnostic stratification and prognostic evaluation prioritized PTPN6 as the most promising candidate. Immune infiltration analysis, scRNA-seq profiling and GSEA collectively demonstrated that PTPN6 expression is predominantly localized to macrophages and functionally involved in modulating the JAK/STAT3 signaling pathway. Functional validation via PTPN6 overexpression in macrophages confirmed its suppressive effects on pro-inflammatory cytokine production, STAT3 phosphorylation, and M1 polarization.

**Conclusion:**

This work identifies PTPN6 as a critical biomarker mechanistically linking insomnia to an exacerbation of SALI, potentially through the amplification of pro-inflammatory responses and JAK/STAT3-dependent macrophage polarization. These findings enhance our understanding of the molecular processes underlying this pathogenic axis; however, further mechanistic investigations and comprehensive clinical validation are required to fully elucidate the complex regulatory network involved.

## 1 Introduction

Insomnia is a prevalent sleep disorder characterized by difficulty initiating and maintaining sleep, representing a global public health challenge. Core symptoms, including frequent nocturnal awakenings and non-restorative sleep, commonly lead to daytime impairments such as cognitive dysfunction and fatigue. Epidemiological studies estimate that insomnia affects approximately 30% of the general population, with a significantly higher prevalence in China (45.5%) (1–3). The condition presents a substantial medical burden and is associated with immune dysregulation, cardiovascular disorders, and an increased risk of depression and anxiety (4–6).

Recent research highlights a significant bidirectional relationship between sleep and immune function. Physiological sleep is crucial for maintaining immune homeostasis, primarily regulated by the sympathetic nervous system and the hypothalamic-pituitary-adrenal axis (7, 8). In contrast, insomnia and sleep deprivation disturb this balance, promoting a pro-inflammatory condition characterized by increased levels of cytokines such as TNF-α, IL-6, and CRP, as well as the deregulation of critical signaling pathways including NF-κB and JAK-STAT (9, 10).Collectively, these findings provide critical insights into the molecular mechanisms underlying systemic inflammation induced by sleep disruption, thereby forming a robust foundation for further explorations into its pathological consequences.

Multiple organ failure arises from sepsis, driven by a dysregulated host response to infection. Among its various complications, sepsis induced acute lung injury (SALI) is the most frequent and deadly consequence (11–14). The pathogenesis of SALI involves disruption of the alveolar-capillary barrier and excessive cytokine release (15–18). Recent Mendelian randomization (MR) has established a causal relationship between sleeplessness and an increased risk of sepsis, suggesting shared pathophysiological pathways (4). Nevertheless, the precise molecular mechanisms by which sleeplessness accelerates the development of SALI are still not well understood, which makes it difficult to create focused risk assessments and treatment plans.

**Figure 1:**
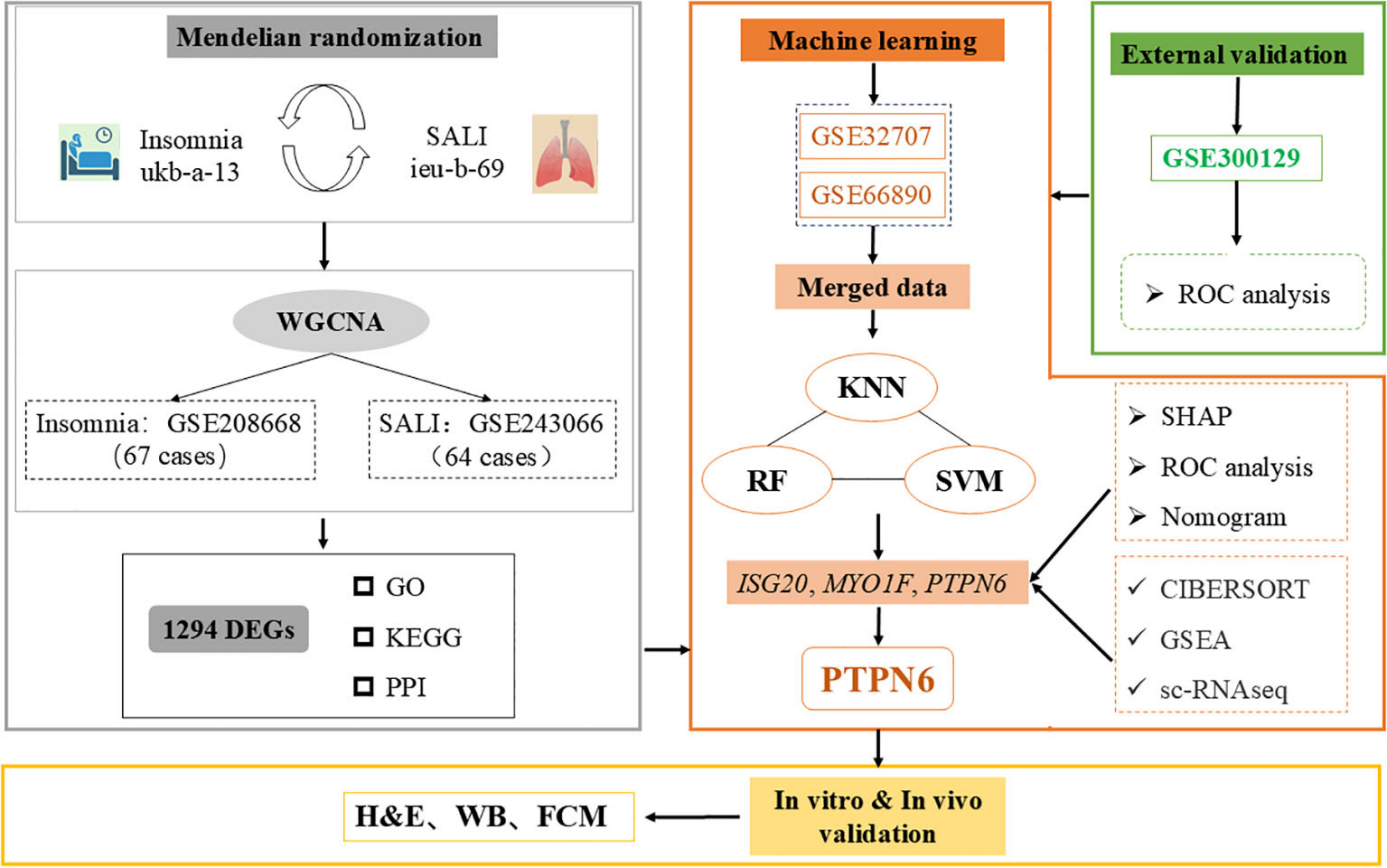
A schematic diagram of the research process.

To address these gaps, this study employs an integrative multi-omics approach that combines MR, weighted gene co-expression network analysis (WGCNA), and machine learning algorithms. A flowchart summarizing the study design is presented in **Figure 1**. We aim to identify pivotal biomarkers and elucidate the mechanistic links between insomnia and SALI. Our findings identify PTPN6 as a critical mediator, potentially operating through the JAK-STAT3 signaling pathway in macrophages to amplify inflammatory responses. This study provides a theoretical basis for early clinical identification of high-risk patients and offers insights for future therapeutic interventions. Further clinical and mechanistic validation is warranted.

## 2 Materials and methods

### 2.1 Mendelian randomization analysis

To investigate the potential causal relationship between insomnia and sepsis, we performed a MR analysis using summary statistics from genome-wide association studies (GWAS). The genetic instrument for insomnia was obtained from a publicly available GWAS dataset (https://opengwas.io/datasets/ukb-a-13; GWAS ID: ukb-a-13) which included 336,965 participants and 10,894,596 single nucleotide polymorphisms (SNPs) (19). The outcome data for sepsis were obtained from the IEU OpenGWAS database (https://gwas.mrcieu.ac.uk/; GWAS ID: ieu-b-69), comprising 462,918 individuals and 12,321,875 SNPs. Both datasets consisted of summary-level statistics, therefore, no additional ethical approval was required.

MR analysis were conducted using the “TwoSampleMR” package in R (20). To ensure robust causal inference, five methods were applied: Inverse-variance weighted (IVW), MR-Egger, weighted median, simple mode, and weighted mode. The IVW and weighted median methods served as the primary estimators. Sensitivity studies were performed to assess potential biases, including Cochran’s Q test for heterogeneity, the MR-Egger intercept test and MR-PRESSO for horizontal pleiotropy, and the leave-one-out analysis to evaluate the impact of individual SNPs. Results were visualized using scatter, funnel, and forest plots to enhance interpretability.

### 2.2 Gene expression data acquisition and processing

Gene expression data were obtained from the National Center for Biotechnology Information Gene Expression Omnibus (NCBI GEO) database (http://www.ncbi.nlm.nih.gov/geo/). A systematic retrieval and rigorous screening process was applied to select high-quality datasets related to insomnia and SALI. For insomnia, we used the GSE208668 dataset, which contains RNA sequencing profiles of peripheral blood mononuclear cells (PBMCs) from 25 insomnia patients and 17 healthy controls. For SALI, we searched the GEO database using keywords such as “ARDS” (Acute Respiratory Distress Syndrome), “ALI”, and “sepsis”. Six key datasets were selected: GSE243066, GSE32707, GSE66890, GSE95233, GSE69063, and GSE276682. Each dataset served a specific analytical purpose: GSE243066 was used to identify clinically relevant genes shared between SALI and insomnia; GSE32707 and GSE66890 were used to create a robust machine-learning prediction model; GSE95233 was used to predict patient prognosis in SALI using XGBoost algorithm; GSE69063 was used to detail disease severity stratification; and GSE276682, a single-cell RNA sequencing (scRNA-seq) dataset, was used to map the key gene expression dynamics across various immune cell subsets (**Table 1**). Together, these datasets formed a multifaceted analytical framework to investigate how insomnia exacerbates SALI pathophysiology.

**Table 1:** Information of GEO datasets containing the insomnia and SALI patients.

Diseases	GEO series	Control case	Sample size	Methodology	Platform
Insomnia	GSE208668	25	42	WGCNA	GPL10904
SALI	GSE243066	15	49	WGCNA, GSEA	GPL30209
	GSE32707	13	47	Machine learning	GPL10558
	GSE66890	0	28	Machine learning	GPL6244
	GSE300129	55	57	External validation	GPL34133
	GSE95233	22	49	Xgboost	GPL570
	GSE69063	11	68	ROC analysis	GPL19983
	GSE276682	3	6	Single-cell RNAseq	GPL24247
	GSE54514	18	53	Kaplan-Meier survival analysis	GPL6947

To increase sample size for machine learning, we merged GSE32707 (13 healthy controls, 47 sepsis cases) with GSE66890 (28 sepsis cases, no controls). The batch origin was defined by dataset (GSE32707 vs. GSE66890), with samples labeled by their GEO accession prefixes (GSM812 and GSM163, respectively). A common gene set was extracted and combined into a single expression matrix. Missing values were imputed using the k-nearest neighbors (kNN) approach. Batch effects were corrected using limma::removeBatchEffect, with dataset origin as the batch covariate and disease status (control vs. case) as the biological design matrix. After batch correction, quantile normalization was applied using normalizeBetweenArrays. Notably, we chose removeBatchEffect rather than ComBat or normalizeBatchEffects, as the former better preserves biological variation while removing technical batch effects in our two-batch scenario. The effectiveness of batch correction was visually confirmed using principal component analysis (PCA), performed with the FactoMineR and factoextra Bioconductor packages. Complete code for batch variable specification, platform information, and step-by-step preprocessing is provided in **Supplementary File 1**.

### 2.3 Weighted gene co-expression network analysis

WGCNA was employed to identify gene modules associated with clinical phenotypes of insomnia and SALI. The analysis was performed separately on the GSE208668 (insomnia) and GSE243066 (SALI) datasets using the WGCNA package (v1.72) in R (v4.3.2) (21).

Expression matrices were preprocessed by removing low-variance genes. Missing values were imputed using row-wise means before the network construction. Sample outliers were identified and removed through Hierarchical clustering based on Euclidean distance. A suitable soft-thresholding power was chosen for each dataset to achieve a scale-free topology (R^2^ > 0.9). The optimal powers were 3 for insomnia and 7 for SALI. An unsigned topological overlap matrix (TOM) was then created. Gene modules were identified using dynamic tree-cutting (minimum module size = 30, deepSplit = 2, merge threshold height = 0.25). Finally, the resulting modules were correlated with numerically encoded clinical traits to identify phenotype−associated gene clusters.

### 2.4 Functional enrichment analysis

Functional enrichment analysis was performed to interpret the biological relevance of the selected gene sets. Gene symbols were first converted to standardized Entrez IDs to ensure annotation consistency. Gene Ontology (GO) enrichment analysis was conducted using the clusterProfiler package (v4.8.1). This analysis covered three categories: Biological Process (BP), Cellular Component (CC), and Molecular Function (MF). Terms with an adjusted p-value and q-value below 0.05were considered statistically significant.

Kyoto Encyclopedia of Genes and Genomes (KEGG) pathway enrichment was performed using the enrich KEGG function with the murine reference database (mmu). Significantly enriched pathways were identified, and the top ten pathways were selected for visualization using customized bar plots to display enrichment scores and statistical significance.

### 2.5 DEGs analysis and construction of PPI network

To systematically characterize the molecular signatures of shared genes implicated in SALI, we profiled their transcriptional dynamics in murine models. Homologous mouse genes corresponding to the identified shared genes were first mapped. Differential expression was then analyzed on in-house murine pulmonary bulk RNA-seq datasets. Differentially expressed genes (DEGs) were defined using stringent statistical thresholds: an adjusted p-value < 0.05 and an absolute log_2_fold-change > 1.5. Detailed protocols and workflows are documented in Section 2.12.

Protein-protein interaction (PPI) networks were reconstructed using the STRING database (https://cn.string-db.org/). To ensure reliability, interactions were filtered based on a minimum confidence score threshold of 0.400 (22), which excluded low-confidence associations. Network topology was analyzed to identify highly interconnected hub genes, and interaction landscapes were visualized to elucidate potential functional modules.

### 2.6 Machine learning model construction

To ensure methodological transparency and prevent information leakage, we established a strict analytical pipeline consisting of the following steps:: (i) Independent feature selection, (ii) dataset merging (GSE32707+GSE66890), (iii) batch correction, (iv) train/test splitting (70/30), (v) model fitting with 5×10 repeated CV, and (vi) independent testing. Batch effects between GSE32707 and GSE66890 were corrected using removeBatchEffect (sva package) after merging but before splitting. Batch variables were incorporated into the design matrix without using phenotype labels. This approach was necessary because batch correction requires the full combined dataset to estimate technical variation. However, we emphasize that all feature selection was performed independently using WGCNA-derived genes from separate insomnia (GSE208668) and SALI (GSE243066) cohorts, as well as mouse lung transcriptome DEGs. None of these features came from the ML datasets.

Stratified random partitioning was used to split the specimens into training (70%) and validation (30%) cohorts. This ensured representative selection while preserving the proportionate representation of phenotypic categories across subsets. Gene expression data was standardized synchronously across both cohorts during preprocessing to meet the computational demands of the methods. A systematic benchmarking procedure was carried out using the caret package, to assess the prediction capabilities of eight traditional machine learning models in a single computing environment. Hyperparameter tuning was performed using 5×10 repeated cross-validation (5-fold CV with 10 iterations) to guarantee model stability and reproducibility during training. The validation cohort’s provided a thorough evaluation of each model’s predicted performance, facilitating the identification of high−confidence molecular biomarkers for SALI diagnosis. This comprehensive approach highlights the potential of including insomnia−related genes as diagnostic markers in SALI and lays the groundwork for future translational applications.

The following eight classical algorithmic approaches were systematically evaluated: Random Forest (RF) enhanced generalization through bootstrap resampling to assemble multiple decision trees (23); Support Vector Machine (SVM) addressed intricate nonlinear classification problems utilizing a radial basis function kernels (24); Generalized Linear Model (GLM) established decision boundaries via binomial logistic regression (25); Gradient Boosting Machine (GBM) iteratively optimized predictive performance through successive refinement (26); K-Nearest Neighbors (KNN) classified specimens by Euclidean distance-based similarity metrics (27); Neural Network with Single Hidden Layer (NNET) implemented a feedforward architecture for nonlinear modeling (28); LASSO Regression performed feature selection and variable compression through L_1_-penalized regularization (27); and Decision Tree (DT) constructed interpretable classification rules following the CART algorithmic framework (29). All models operated in probabilistic output mode. Predictive performance was comprehensively evaluated using metrics including the area under the receiver operating characteristic curve (AUC). Generalizability was further assessed by testing all eight pre-trained models on an independent external cohort (GSE300129), which had been completely withheld from all prior analytical steps.

Residual analysis was performed using the DALEX package. Distribution histograms, residual boxplots, and reversed cumulative distribution curves were generated. Multi-model ROC curves were created using the pROC software, and precision-recall curves were examined to account for class imbalance. Key genetic drivers were identified by extracting feature significance rankings from the optimum model.

### 2.7 Identification of key genes via RF, SVM, and KNN algorithms

The randomForest package was used to construct and optimize the RF model. The basic setup started with 500 decision trees, and the Out-of-Bag (OOB) error was used as the main evaluation measure to guide model development. To improve the accuracy of classification, a methodical error analysis process was implemented. The OOB error rate was monitored across a range of decision tree, and the optimal tree ensemble size was determined by identifying the turning point corresponding to the minimum OOB error. This facilitated the assembly of a more accurate prediction system. Feature importance was quantitatively assessed using the Mean Decrease Accuracy measure. To determine the precise contribution of each gene to predictive accuracy, the difference in model classification accuracy was evaluated by randomly switching around certain gene expression levels. This approach enabled the exact quantification of each gene contributed to the accuracy of the predictions. Consequently, a ranked list of feature genes was generated in descending order of importance.

The SVM framework used seven feature dimensionality gradients (1/10/30/50/70/90/110 genes) and a 5-fold repeated cross-validation approach. The discriminating performance of each feature subset was assessed using the Root Mean Squared Error (RMSE) metric, with a linear kernel SVM serving as the core classifier. Grid search parameterization was used to determine the optimal feature dimensionality, which corresponds to the feature set yielding the lowest cross-validated RMSE. Subsequently, the top 10 key feature genes were selected based on variable significance scores derived from the varImp function.

Recursive Feature Elimination (RFE) was optimized within KNN architecture. Six feature subset sizes were evaluated using sixfold cross-validation. With a KNN classifier (k=5) as the computational engine, feature selection increasingly included predictive factors. The ideal feature subset dimensionality was found by cross-validation classification accuracy. Using these diagnostic characteristics, a reconstituted KNN classifier (k=5) was created and fivefold cross-validated. Finally, boxplot visualizations showed transcriptional variability between categorization groupings by examining crucial gene expression patterns.

### 2.8 Interpretable ML models

An interpretable modeling approach was conducted using the eXtreme Gradient Boosting (XGBoost) method on the GSE95233 patient survival dataset. This framework evaluated the genes selected by RF, SVM, and KNN models. The dataset was divided into 70% training and 30% independent testing cohorts using stratified sampling. This ensured proportional representation of phenotypic categories across both subsets. Optimized model settings included maximum tree depth to 3(max_depth=3), learning rate to 0.1 (η=0.1), and training iterations to 10 (nrounds=10). Prognostic contributions were assessed using the mean absolute SHAP (SHapley Additive exPlanations) values. This metric quantified the magnitude of each feature’s impact within a binary logistic regression loss function framework. Waterfall visualizations showed the positive or negative effect of different genes on expected survival outcomes for each specimen to identify gene-specific contributions. Furthermore, dependent plots were constructed to reveal relationships between the gene expression levels and anticipated survival risk. The SHAP mean absolute value as the primary evaluation metric. It quantified the prognostic utility of each feature within the prediction model. This thorough method revealed the importance of potential biomarker and supported the development of a robust survival analysis prediction architecture.

### 2.9 Construction of the nomogram model and assessment of diagnostic marker prediction model

This study utilized the GSE69063 database to develop a multistage biomarker modeling framework. The framework aimed to predict the severity progression of SALI disorder. The analytical pipeline started with multivariate logistic regression to synthesize critical hub gene expression signatures. A stratification model for severity prognosis was then constructed. Model calibration robustness was rigorously assessed using a 1000-cycle bootstrap validation approach, which yielded graphical concordance evaluations. The evaluations compared predicted probabilities with observed clinical outcomes. To quantify diagnostic effectiveness, comparative ROC analyses were performed to systematically evaluate against that of individual hub gene classifiers. This benchmarking provided insights into the model’s ability to differentiate between varying severity levels, thereby underscoring its diagnostic precision. To enhance clinical applicability, two visualization tools were developed. First, a static nomogram interface was constructed. Logarithmic transformation algorithms were employed to convert linear predictor variables into clinically interpretable severity probability metrics. Second, a consolidated probabilistic projection diagram was introduced. This diagram integrated kernel density distributions with boxplot visualizations. This dual-layer visualization framework simultaneously depicted individual patient risk trajectories and their corresponding 95% confidence interval boundaries. It provided an intuitive overview of prognostic risk assessments. Together, these methodologies and tools establish a robust, clinically deployable system for stratifying SALI disorder severity progression. The system holds potential implications for precision medicine applications.

### 2.10 Immune cell infiltration and correlation analysis

Immune cell infiltration analysis was performed using the CIBERSORT deconvolution framework with a mouse-adapted immune signature matrix. Unlike the classical LM22 reference derived from human immune cells, this study utilized a murine immune signature matrix as described by Chen et al. (30). It contains gene expression signatures representing murine immune cell populations and enables estimation of immune cell composition in mouse bulk RNA-seq datasets. Normalized murine lung RNA-seq expression profiles were used as input for the deconvolution procedure. Immune cell proportions were estimated using the modified CIBERSORT algorithm implemented in R. A total of 1000 permutations were performed to ensure statistical robustness. Spearman’s rank correlation analysis was subsequently applied to assess the relationships between hub gene expression levels and immune cell infiltration abundance. This method revealed the architecture of putative gene-immune interactions. It also offered crucial insights into the molecular underpinning of the regulatory interaction between important genes and immune dynamics.

### 2.11 Gene correlation and GSEA analysis

This research used the GSE243066 standardized expression matrix to explore transcriptional co-regulatory networks among important hub genes to better understand their functions. The first phase used non-parametric Spearman’s rank-correlation analysis to evaluate target hub gene-genome-wide expression profiles correlations. In parallel, bivariate scatterplots were created using standardized hub gene expression levels on the abscissa and covariate gene expression magnitudes on the ordinate. This showed critical intergenic regulatory structures. After ranking genes by absolute Spearman correlation coefficients, Gene Set Enrichment Analysis (GSEA) was performed using computational techniques based on the MSigDB repository’s authorized Hallmark gene set collection (h.all.v2024.1.Hs.symbols.gmt). This method deconstructed hub gene molecular pathways, revealing their functional regulatory networks. This integrative analytical paradigm quantified transcriptional concordance dynamics and provided a systems-level understanding of hub gene activity operational mechanisms, revealing their biological significance.

### 2.12 Single-cell RNA analysis of hub genes

Additionally, this study added to the GSE276682 single-cell transcriptome collection by including three biological control replicates and experimental groups that were given 30 mg/ml LPS intraperitoneally over a 72-hour period. In order to make sure the results of the analysis were accurate, a strict quality control system was set up. This system weeded out transcriptionally competent cells (200 < nFeature RNA < 4500) and mitochondrial-compromised outliers (>5% mitochondrial gene fraction). The next step in the analysis used a series of computer steps: first, the dimensionality was reduced using PCA; then, the Harmony algorithm was added to get rid of technical issues by fixing batches of effects; finally, the cellular subpopulations were sorted by using optimized parameterization (resolution = 0.4); this led to the definitive phenotyping of separate cellular subsets that allowed a full description of differential hub gene expression dynamics and their compartment-specific distribution paradigms within the cellular ecosystem.

### 2.13 *In vivo* experimental validation

Male C57BL/6 mice (6~8 weeks old, 25 ± 3 g) were housed under specific pathogen-free conditions. All procedures were approved by the Institutional Animal Care and Use Committee of the Tenth Affiliated Hospital of Southern Medical University (Approval No. IACUC-AWEC-202401503).

Following a 7-day acclimatization under SPF conditions, mice were stochastically allocated into four cohorts: (1) Ctrl (unmanipulated controls), (2) SD (21-day sleep deprivation via gentle handling/rotating platform, 20 h/day), (3) LPS (SALI induced by intranasal instillation of 40 μL Lipopolysaccharide [LPS,1.25 μg/μL] under anesthesia), and (4) SD+LPS (undergoing SD followed by identical LPS administration). The 21-day SD paradigm was selected based on established protocols demonstrating sustained sleep restriction without excessive stress (31). All procedures were performed during the animals’ inactive phase to minimize circadian confounders, with LPS groups receiving standardized anesthesia and transnasal delivery while Ctrl/SD cohorts remained undisturbed unless sampled for parallel endpoints. After twelve hours, mice were euthanasia by CO2 asphyxiation, and pulmonary tissues were expeditiously harvested and partitioned preservation in RNA stabilization medium. Lung tissues from the Ctrl, SD, LPS, and SD+LPS groups were collected for comprehensive analysis, including RT-qPCR to validate gene expression, and histopathological examination (H&E staining) performed by Shanghai Servicebio Technology Co., Ltd.

Nucleic acid isolation was conducted employing the commercially procured purification suite (Tissue RNA Purification Kit, Cat.No. EZB-RN001A; EZBioscience), wherein approximately 40 mg of freshly harvested pulmonary tissue was subjected to mechanical homogenization within 500 μL of snap-cooled Lysis Buffer. The purification protocol meticulously adhered to manufacturer specifications through sequential introductions of chloroform, absolute ethanol, Wash Buffer, and Elution Buffer. Subsequent complementary DNA synthesis utilized the Color Reverse Transcription Kit (EZBioscience, Cat.No. A0010CGQ) to reverse-transcribe 1 μg of isolated RNA. Quantitative transcriptional analysis proceeded via real-time fluorescence quantification using the 2x Color SYBR Green qPCR Master Mix (EZBioscience, Cat.No.A0012-R2) executed on a dedicated detection platform. Experimental integrity was safeguarded through systematic implementation of technical replicates across all specimens, with relative transcriptional quantitation determined through the 2^-ΔΔCt^ algorithm. Oligonucleotide primer sequences are cataloged in **Table 2**. Blank contamination controls were instituted throughout experimental procedures to ensure amplification specificity.

**Table 2:** RT-qPCR primers.

Primers	Sequences (5’-3’)
β-actin	Reverse: CCAGTTGGTAACAATGCCATGT
	Forward: GGCTGTATTCCCCTCCATCG
IL-1β	Reverse: TGGGTATTGCTTGGGATCCA
	Forward: ACCTGTCCTGTGTAATGAAAGACG
IL-6	Reverse: AAGTGCATCATCGTTGTTCATACA
	Forward: ATCGTGGAAATGAGAAAAGAGTTGT
TNF-α	Reverse: TCAGCCACTCCAGCTGCTC
	Forward: CATCTTCTCAAAATTCGAGTGACAA
PTPN6	Reverse: CCCGAGTAGCGTAGTAAGGCT
	Forward: CCCGCTCAGGGTCACTCATA

### 2.14 *In vitro* experimental validation

RAW264.7 cells (cat. no. CL-0190; Procell Life Science & Technology Co., Ltd.) were cultured in DMEM (cat. no. C11995500BT; Gibco) supplemented with 10% FBS (cat. no. FND500; Shanghai ExCell Biology, Inc.) and confirmed mycoplasma-negative using MycoBlue Mycoplasma Detector kit (cat. no. D101; Vazyme Biotech Co., Ltd.). Lentiviral vectors encoding mouse Ptpn6 (pLV3-EF1a-Ptpn6-3×FLAG-EGFP-Puro; MiaoLing Plasmid Platform, G104487) or empty vector (pLV3-EF1a-MCS-3×FLAG-EGFP-Puro; MiaoLing Plasmid Platform, P83822) were used to generate oe-Ptpn6 and oe-Ctrl stable cell lines, respectively. Cells were stimulated with 1 μg/ml LPS (cat. no. L2630; MilliporeSigma) for indicated durations prior to mRNA and protein collection.

### 2.15 Western blotting

Total protein was extracted using RIPA lysis buffer (cat. no. WB3100; Suzhou NCM Biotech Co., Ltd.) supplemented with protease inhibitor cocktail (cat. no. HY-K0010; MedChemExpress) and phosphatase inhibitor (cat. no. GRF102; Epizyme Biotech Co., Ltd.). Protein concentration was quantified by BCA assay (cat. no. P0009; Beyotime Biotechnology). Equal amounts of protein (25 μg/lane) were separated on 8–10% SDS-PAGE gels and transferred to 0.22 μm PVDF membranes (cat. no. ISEQ00010; MilliporeSigma). Membranes were blocked with 5% non-fat milk (cat. no. 36120ES76; Shanghai Yeasen Biotechnology Co., Ltd.) for 1 h at room temperature, then incubated overnight at 4 °C with primary antibodies: anti-GAPDH (cat. no. LF205; Epizyme Biotech), anti-PTPN6 (cat. no. YM8819; Immunoway Biotechnology), anti-p-STAT3 (cat. no. YM8551; Immunoway Biotechnology), and anti-CD86 (cat. no. YM8023; Immunoway Biotechnology). After washing, membranes were incubated with HRP-conjugated secondary antibodies (1:5,000; cat. no. E-LF101/LF102; Epizyme Biotech Co., Ltd.) for 1 h at room temperature. Signals were detected using ECL reagents (cat. no. 1705061; Bio-Rad Laboratories) and imaged on a ChemiDoc system (G:B0XChemiXX9; Syngene).

### 2.16 Flow cytometry

RAW264.7 cells were stained with BV421-conjugated anti-CD86 (Biolegend, 105031), and PE-conjugated anti-CD206 (BD Biosciences, 3222347). Live cells were selected based on FSC-A/SSC-A profiles. M1 (CD86^+/^CD206^−^) and M2 (CD86^−/^CD206^+^) populations were quantified as a percentage of total live cells.

### 2.17 Statistical analyses

Comprehensive biostatistical analyses were conducted exclusively within the computational environments of R (v 4.3.2) and GraphPad Prism (v 9.5.0). Differential gene expression identification was executed through implementation of the DESeq2 package. Correlation analyses between hub genes and immune cell infiltration levels were performed using Spearman’s rank correlation coefficient. Intergroup comparative analyses for lung injury score and PCR-derived data were subjected to Student’s t-test framework. Threshold for statistical significance was established at p < 0.05. False discovery rate (FDR) correction was administered utilizing the Benjamini–Hochberg procedure to adjust raw p-values. Statistical significance gradation was denoted as follows: *P < 0.05, **P < 0.01, ***P < 0.001.

## 3 Results

### 3.1 Causal relationship between insomnia and sepsis

**Figure 2:**
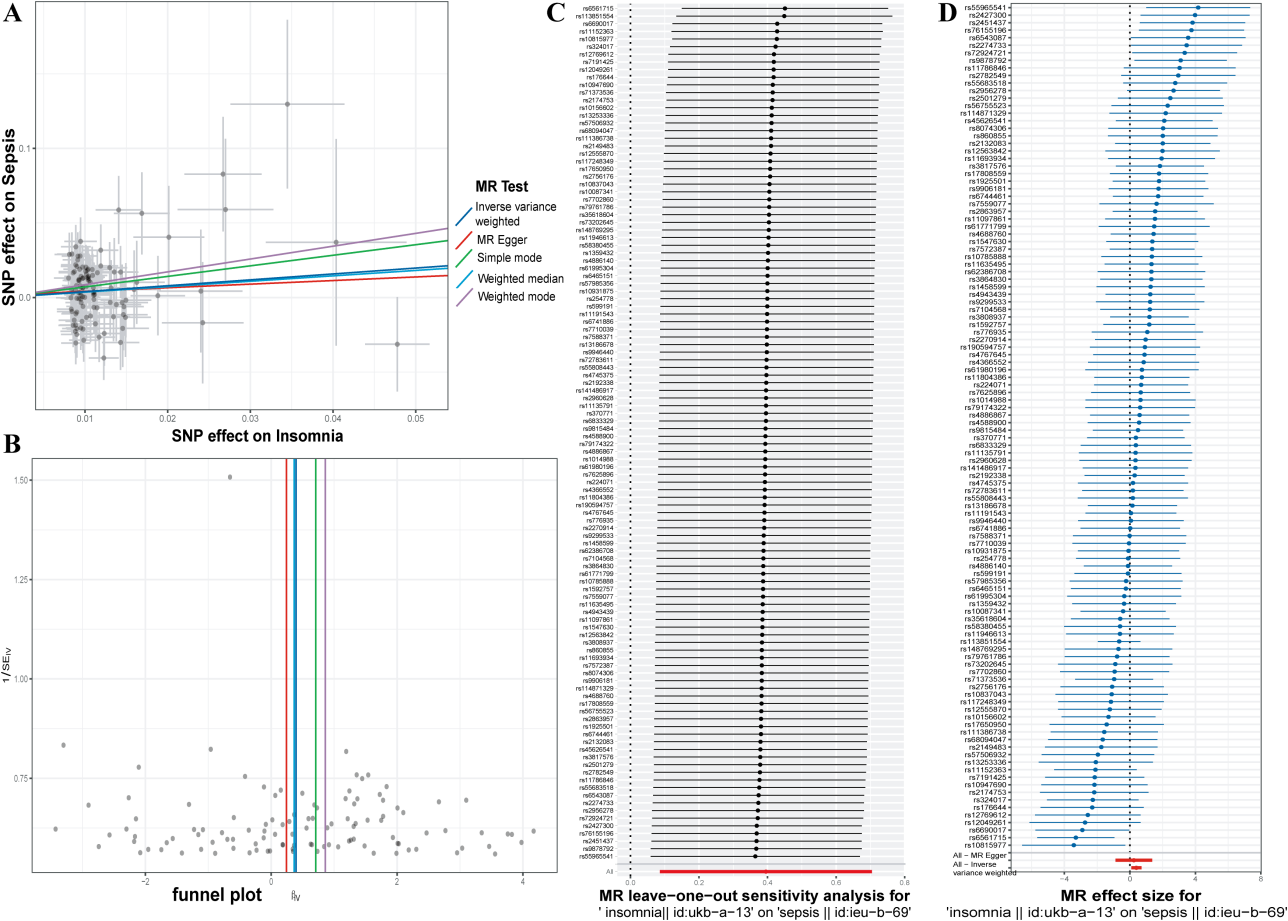
Results of Mendelian randomization analysis. **(A)** Scatter plot of genetic variant-outcome associations. **(B)** Funnel plot assessing potential pleiotropy. **(C)** Leave-one-out sensitivity analysis for robustness evaluation. **(D)** Forest plot displaying effect estimates of individual instrumental variables.

After extensive sensitivity analyses addressing heterogeneity and horizontal pleiotropy, we established insomnia as a genetic risk factor for sepsis (**Figure 2**; **Supplementary Tables 1**, **2**). Leveraging the most conservative MR estimator—fixed-effect inverse-variance weighted (IVW) regression—across 112 rigorously selected instrumental SNPs, genetically predicted insomnia conferred a markedly elevated risk of sepsis (β = 0.395, SE = 0.158, P = 0.012). Sepsis induced acute lung injury (SALI) is the most frequent and deadly consequence (11–14).

These findings support insomnia as a risk factor for sepsis However, the inferential scope of these findings requires careful clarification. Our MR analysis establishes a causal relationship between insomnia and sepsis risk, not directly with SALI. SALI represents a major complication and downstream manifestation of sepsis rather than an independent outcome in our genetic framework. Therefore, the extension of our MR findings to SALI interpreted as follows: insomnia increases the risk of sepsis, and given that SALI develops in a substantial proportion of sepsis patients, insomnia may indirectly contribute to SALI susceptibility through its effect on sepsis risk. We subsequently conducted experimental validation using sleep deprivation and LPS-induced lung injury models to specifically address the insomnia-SALI link.

### 3.2 Construction of WGCNA and gene modules screening

**Figure 3:**
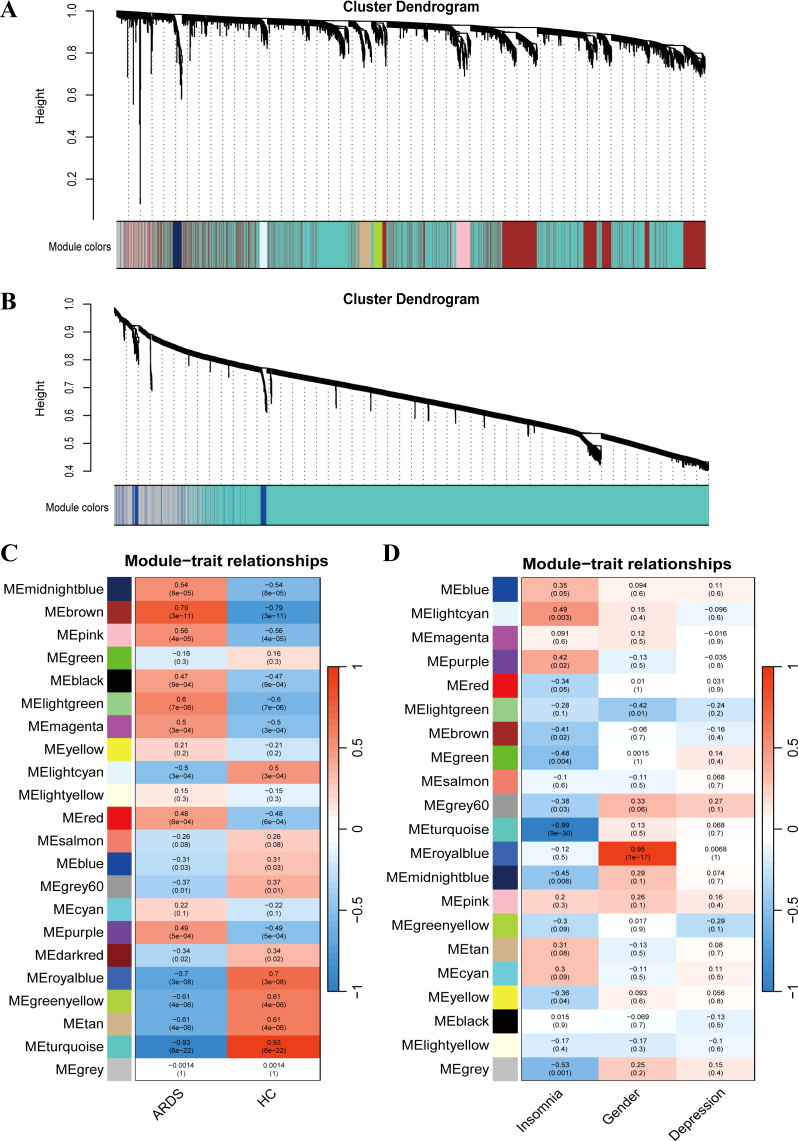
WGCNA results for insomnia and SALI. **(A, B)** Module clustering dendrograms constructed from gene expression profiles, where distinct colors represent independent co-expression gene modules, reflecting functional cooperative relationships in transcriptional regulation. **(C, D)** Module-trait relationship heatmaps display correlations between modules and target clinical characteristics. The upper numerical values indicate Pearson correlation coefficients (blue: negative correlation; red: positive correlation), while the lower values represent corresponding significance p-values.

We performed an integrated, cross-cohort analysis of two publicly available GEO datasets: the insomnia-related transcriptome (GSE208668) and the SALI dataset (GSE243066). WGCNA partitioned the GSE243066 cohort into 22 discrete module eigengenes (MEs) and the GSE208668 cohort into 21 (**Figures 3A, B**; **Supplementary Figure 1**). Phenotype–module relationships were interrogated using module membership (MM) coefficients; only those modules meeting a stringent |MM| threshold of > 0.6 and simultaneous statistical significance (*P < 0.05*) were advanced for downstream analyses. Quantifiable associations with SALI pathogenesis identified six significant modules: “brown” (MM = 0.79, p=3×10^−11^), “greenyellow” (MM=-0.61, p=4×10^−6^), “lightgreen” (MM = 0.6, p=7×10^−6^), “royalblue” (MM=-0.7, p=3×10^−8^), “tan” (MM=-0.61, p=4×10^−6^), and “turquoise” (MM=-0.93, p=6×10^−22^). Contrastingly, insomnia-related scrutiny exclusively yielded the “turquoise” module (MM=-0.99, p=9×10^−30^) as significant (**Figures 3C, D**; **Supplementary Table 3**). Cumulatively, this study delineated 4,690 insomnia-associated genes and 6,621 SALI-related genes, which are posited to mediate their respective disease pathogenesis, thereby furnishing prospective molecular targets for subsequent investigation.

### 3.3 Analysis of the shared genes and functional enrichment

**Figure 4:**
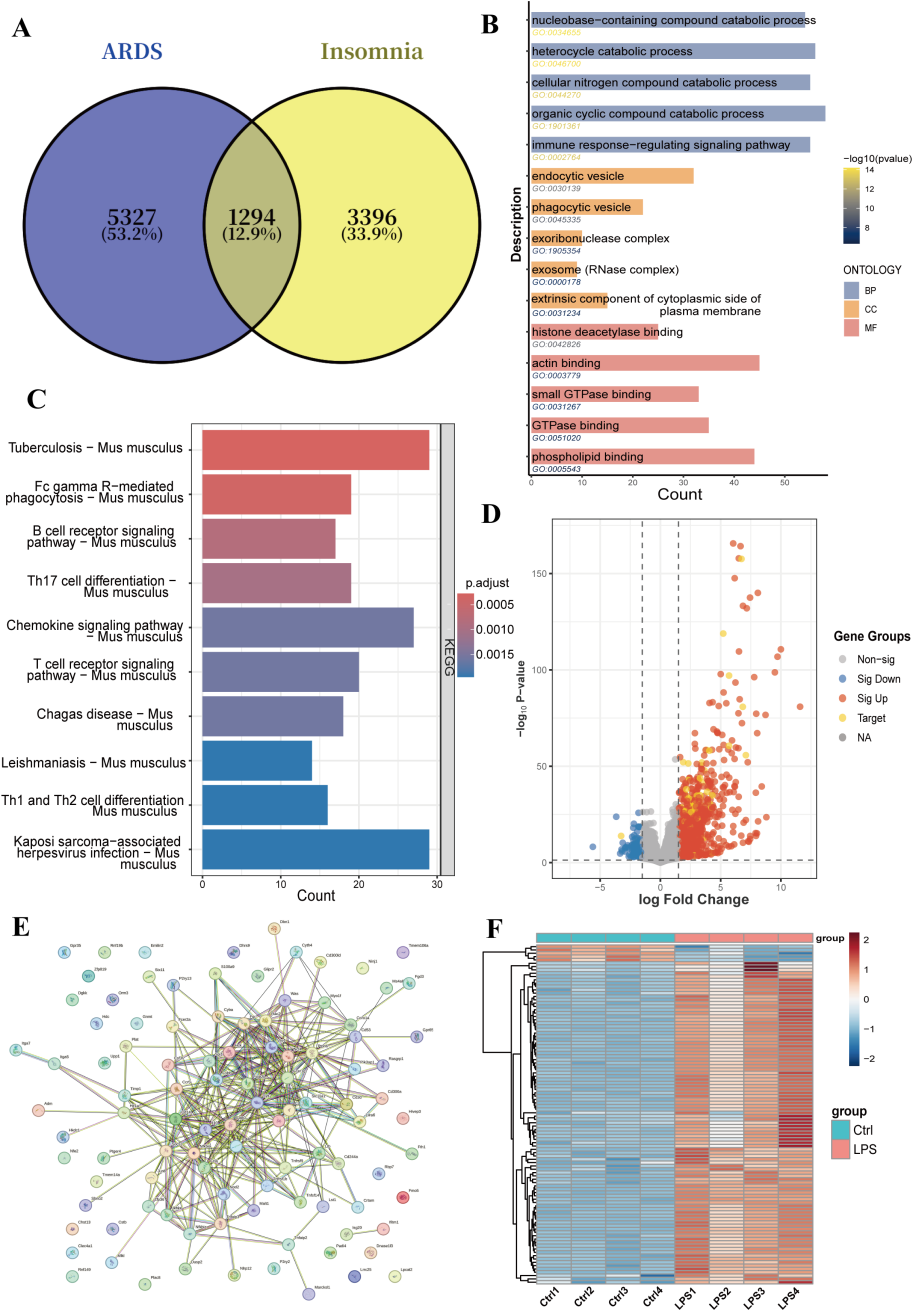
Differential expression, pathway enrichment, and PPI analysis of shared genes. **(A)** Venn diagram illustrating intersecting gene sets (1,294 shared genes) between insomnia and SALI. **(B)** GO enrichment results of shared genes (blue bars: biological processes; yellow bars: cellular components; red bars: molecular functions; GO terms beneath bars are color-graded based on statistical significance, with increasing yellow intensity denoting smaller p-values). **(C)** KEGG enrichment results of shared genes (bar color intensity reflects significance, with deeper red indicating greater enrichment). **(D)** DEG analysis from RNA-seq data (x-axis: logFC; y-axis: p-value; gray dots: genes with p>0.05 and |logFC|<1.5; blue dots: p<0.05 and logFC<-1.5; red dots: p<0.05 and logFC>1.5). **(E)** PPI network of 112 DEGs. **(F)** Heatmap of DEG expression profiles (tiles indicate relative expression, with red denoting upregulation and blue downregulation).

Through WGCNA analysis, we identified 6,621 SALI-associated genes and 4,690 insomnia-related genes, with subsequent intersection revealing 1,294 shared genes (**Figure 4A**). Anticipating *in vivo* validation, orthologous conversion of these human peripheral blood-derived shared genes was performed using g:Profiler(https://biit.cs.ut.ee/gprofiler/orth), yielding 1,163 reliable murine orthologs.

Functional characterization of insomnia-SALI shared genes demonstrated significant enrichment across 1,506 GO terms. Biological processes predominated (BP, 85.79%), followed by cellular components (CC, 5.98%) and molecular functions (MF, 8.23%), principally encompassing immune response, phagocytic vesicle formation, and histone deacetylase binding activities (**Figure 4B**; **Supplementary Table 4**). These genes concurrently enriched 331 KEGG pathways, of which 41 achieved statistical significance (p.adjust < 0.05). Principal pathways included Tuberculosis, Fc gamma R-mediated phagocytosis, B cell receptor signaling pathway, and Chemokine signaling pathway (**Figure 4C**; **Supplementary Table 5**).

In LPS-induced SALI modeling, 102 shared genes exhibited significant differential expression (p-value < 0.05 and |logFC| > 1.5) relative to controls (**Figure 4D**). Subsequent PPI network analysis revealed pronounced functional connectivity (**Figure 4E**), while differential expression heatmaps delineated contextual expression patterns (**Figure 4F**). These findings substantiate significant molecular convergence between insomnia and SALI within immunoregulatory and inflammatory cascades, providing compelling molecular corroboration of their pathophysiological interplay and nominating actionable therapeutic targets.

### 3.4 Identify potential shared diagnostic genes based on machine learning algorithms

**Figure 5:**
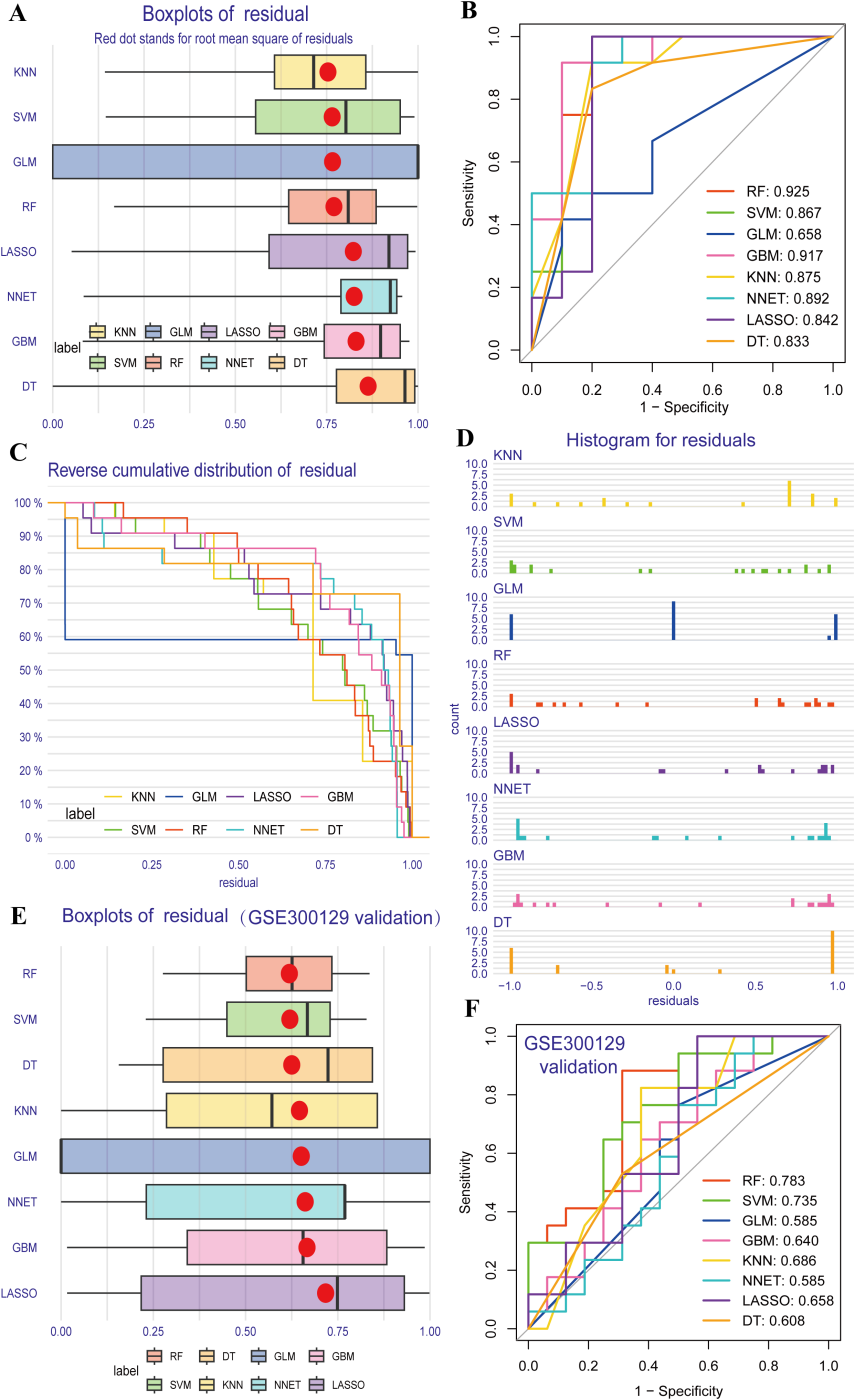
Machine learning model construction and evaluation for eight typical algorithms. **(A)** Boxplots of residuals across all eight machine learning models. RMSEs are denoted by red dots, where proximity to zero indicates reduced systematic bias; narrower boxplot ranges reflect greater predictive stability. **(B)** ROC curves for all models, plotting sensitivity (y-axis) against 1-specificity (x-axis); AUC values quantify the area under each ROC curve. **(C)** Cumulative residual distributions across models (x-axis: residual magnitude). **(D)** Residual histograms, where symmetrical distribution around zero with reduced peak height signifies optimal model fit with minimized systematic bias. **(E, F)** Residual boxplots and ROC curves of feature genes in GSE300129 validation.

To systematically screen diagnostically valuable core genes among DEGs, we integrated GSE32707 and GSE66890 databases, conducting comprehensive evaluation of diagnostic performance across eight machine learning algorithms including RF, SVM, and LASSO. SVM, KNN, and RF models showed superior performance. They had minimal residuals without systematic bias and enhanced predictive stability (reflected in compact interquartile ranges) (**Figure 5A**). ROC curve analysis demonstrated the RF algorithm achieved optimal diagnostic discrimination (AUC = 0.925), surpassing SVM (0.867), GBM (0.917), KNN (0.875), and NEET (0.892) (**Figure 5B**). Cumulative residual distributions and residual histograms further validated the exceptional fitting efficacy of KNN, SVM, and RF models via minimal aggregate residuals and approximately normal distributions (characterized by bell-shaped curves and diminished histogram peak values), confirming absence of significant systematic deviations (**Figures 5C, D**).The observed performance attenuation in external validation (GSE300129: RF AUC = 0.783 vs. discovery AUC = 0.925) is consistent with well-documented phenomena of overfitting in high-dimensional genomic data and dataset-specific batch effects. Importantly, the relative ranking of algorithms remained stable, with RF and SVM consistently outperforming other methods, supporting their selection as optimal classifiers. The external validation AUC of 0.783 still represents clinically meaningful discrimination, comparable to or exceeding established biomarker signatures in critical care settings (**Figures 5E, F**).

**Figure 6:**
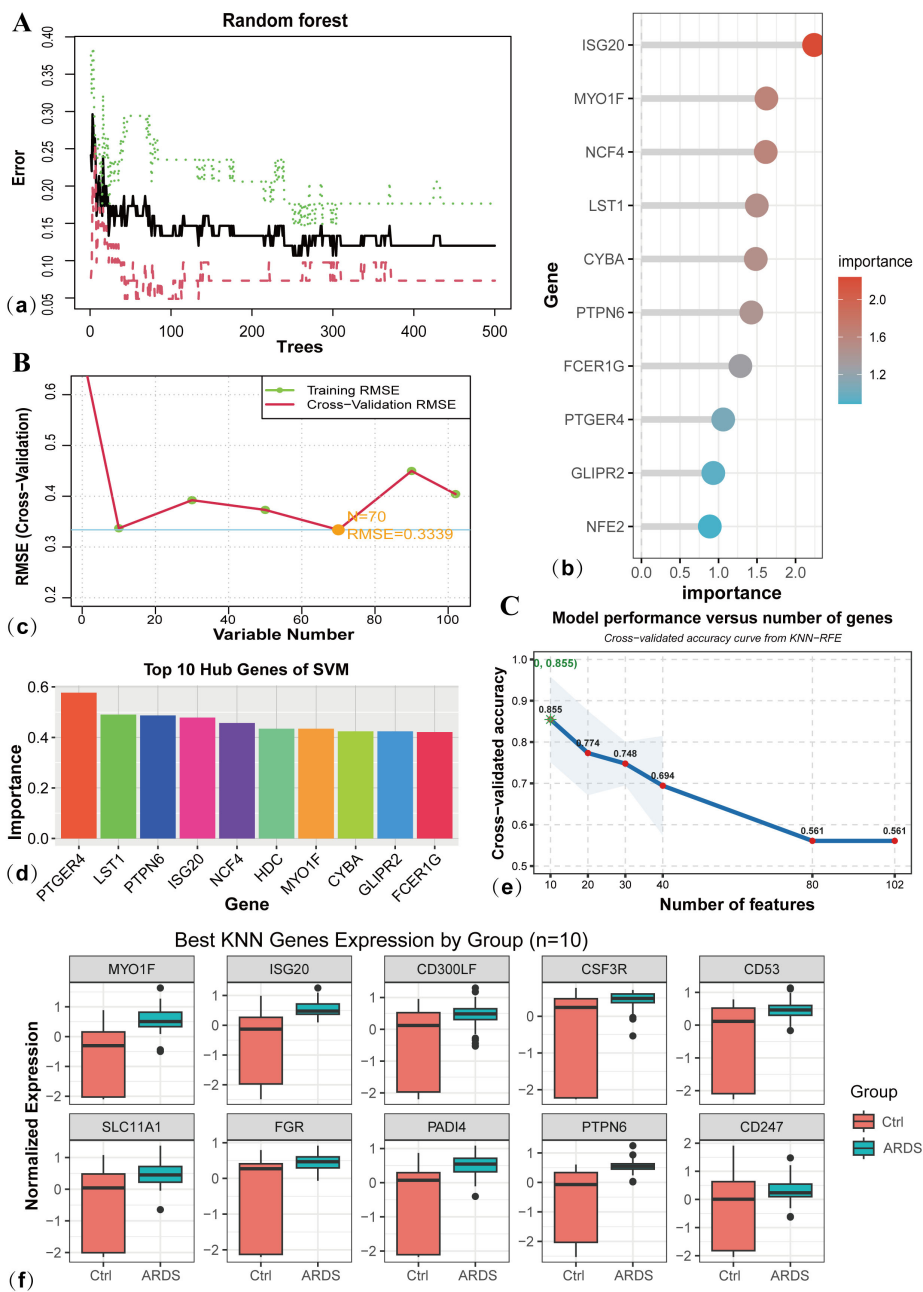
Top 10 feature genes selected by RF, SVM, and KNN models. **(A)** RF model: **(a)** Schematic representation of the 500 decision tree ensemble; **(b)** the top 10 most important feature genes identified by feature importance scoring. **(B)** SVM algorithm: **(c)** Stepwise feature subset selection via RFE, with mean squared error on the y-axis reflecting prediction accuracy relative to ground truth labels; **(d)** the top 10 biomarker genes prioritized by SVM-RFE. **(C)** KNN model: **(e)** Performance characteristic curve demonstrating non-linear accuracy progression with incremental feature inclusion, where the optimal feature subset is automatically determined by algorithmic optimization; **(f)** Expression profile boxplots of the top 10 core genes across molecular subtypes, highlighting distinct distribution patterns.

Based on these comparative findings, RF, SVM, and KNN were designated optimal algorithms for core gene selection. Intersection analysis of each model’s top-ten ranked feature genes yielded three consensus biomarkers: *ISG20*, *MYO1F*, and *PTPN6*. Specifically, the RF model identified *ISG20*, *MYO1F*, *NCF4*, *LST1*, *CYBA*, *PTPN6*, *FCER1G*, *PTGER4*, *GLIPR2*, and *NFE2* through 500 decision trees (**Figure 6A**). SVM’s top features included *PTGER4*, *LST1*, *PTPN6*, *ISG20*, *NCF4*, *HDC*, *MYO1F*, *CYBA*, *GLIPR2*, and *FCER1G* (**Figure 6B**), while KNN encompassed *MYO1F*, *ISG20*, *CD300LF*, *CSF3R*, *CD53*, *SLC11A1*, *FGR*, *PADI4*, *PTPN6*, and *CD247* (**Figure 6C**; **Supplementary Table 6**). These findings constitute rigorously validated molecular candidates for clinical diagnostics, with the convergent biomarkers *ISG20*, *MYO1F*, and *PTPN6* demonstrating salient diagnostic utility and translational potential.

### 3.5 Model interpretability of hub genes for disease prognostic prediction

**Figure 7:**
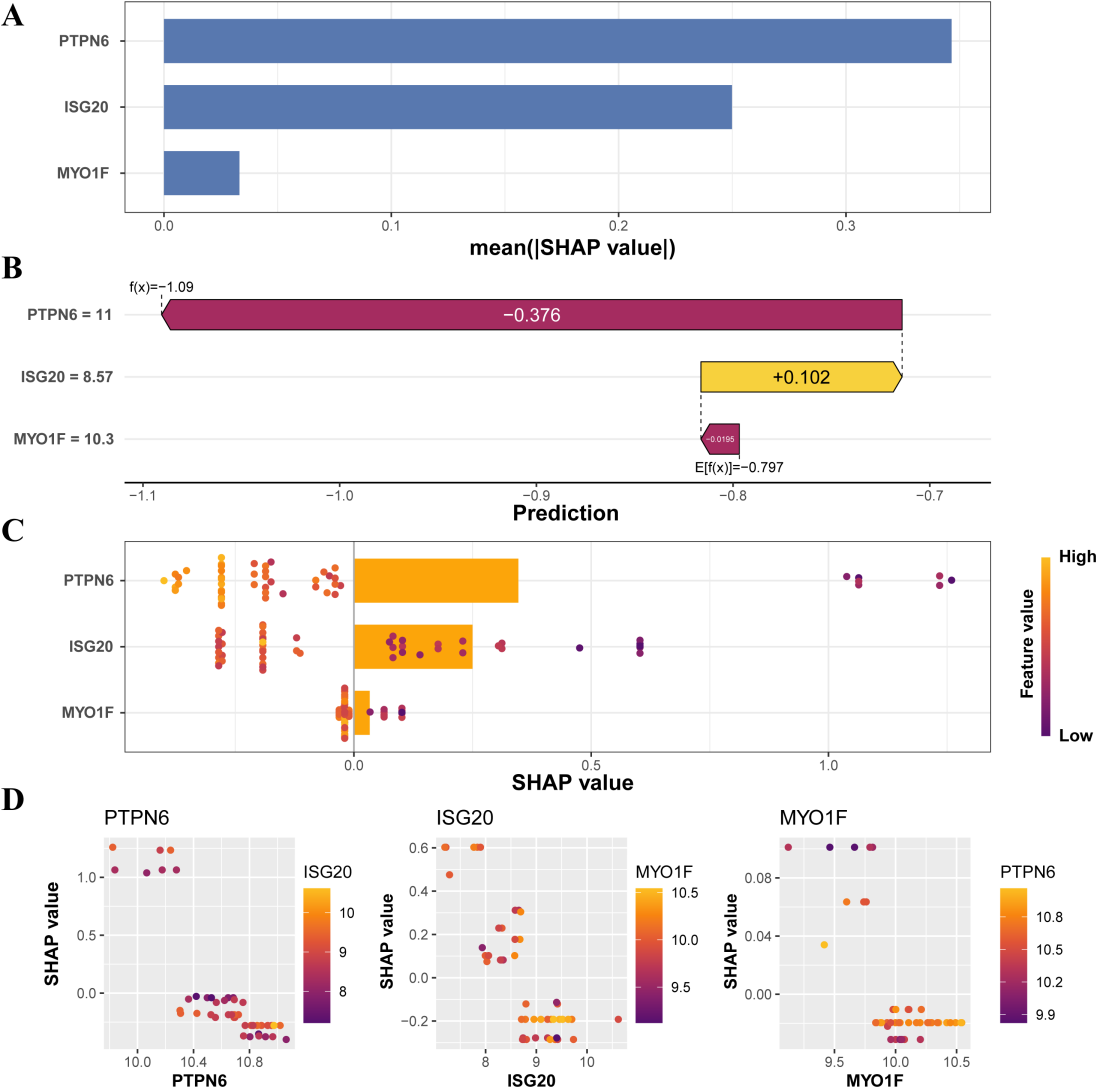
Interpretation of the SALI prognostic model through SHAP explainable. **(A)** Global feature importance bar plot displaying the mean absolute SHAP values for each biomarker, quantifying the relative contribution of hub genes to model predictions. **(B)** Individual biomarker SHAP waterfall plot illustrating how specific gene expression levels influence the predicted outcome for a representative sample (x-axis: predicted risk score; y-axis: feature value impact on prediction probability). **(C)** SHAP value beeswarm plot where each point represents a sample, with color intensity encoding feature magnitude (dark = high expression, light = low expression) and horizontal position indicating effect direction and magnitude. **(D)** SHAP dependence plots revealing non-linear relationships between biomarker values and their corresponding SHAP values, while visualizing potential feature interactions through secondary variable stratification.

To establish a reliable prognostic prediction model for sepsis, we implemented an XGBoost-based framework incorporating SHAP interpretability for evaluating training set prediction contributions. SHAP values quantitatively delineate feature genes’ directional influence on outcomes: positive values elevate mortality likelihood, whereas negative values enhance survival probability. It is important to note that SHAP values indicate predictive attribution within the model and do not establish biological causality. This integrated model incorporated 28-day prognostic data from general septic patients without SALI or lung injury ascertainment (GSE95233), specifically interrogating associations between clinical outcomes and three key biomarkers—*PTPN6*, *ISG20*, and *MYO1F*. While these patients represent a broader sepsis population rather than SALI-specific cohorts, we posit that the identified prognostic signatures may extend to sepsis-associated lung injury given the shared pathophysiological mechanisms and immune dysregulation. SHAP summary analysis identified *PTPN6* as the most consequential predictive feature (highest mean |SHAP|), followed by *ISG20* and *MYO1F*s (**Figure 7A**). Univariate SHAP interpretation revealed elevated *PTPN6* (SHAP=-0.376) and *MYO1F*(SHAP=-0.0195) expression substantially augmented survival probability, whereas heightened expression of *ISG20* (SHAP = 0.102) mildly elevated mortality risk (**Figures 7B, C**). This suggests PTPN6 as a potential protective indicator, with its SHAP contributions exhibiting context-dependent modulation by *ISG20* levels (**Figure 7D**).

**Figure 8:**
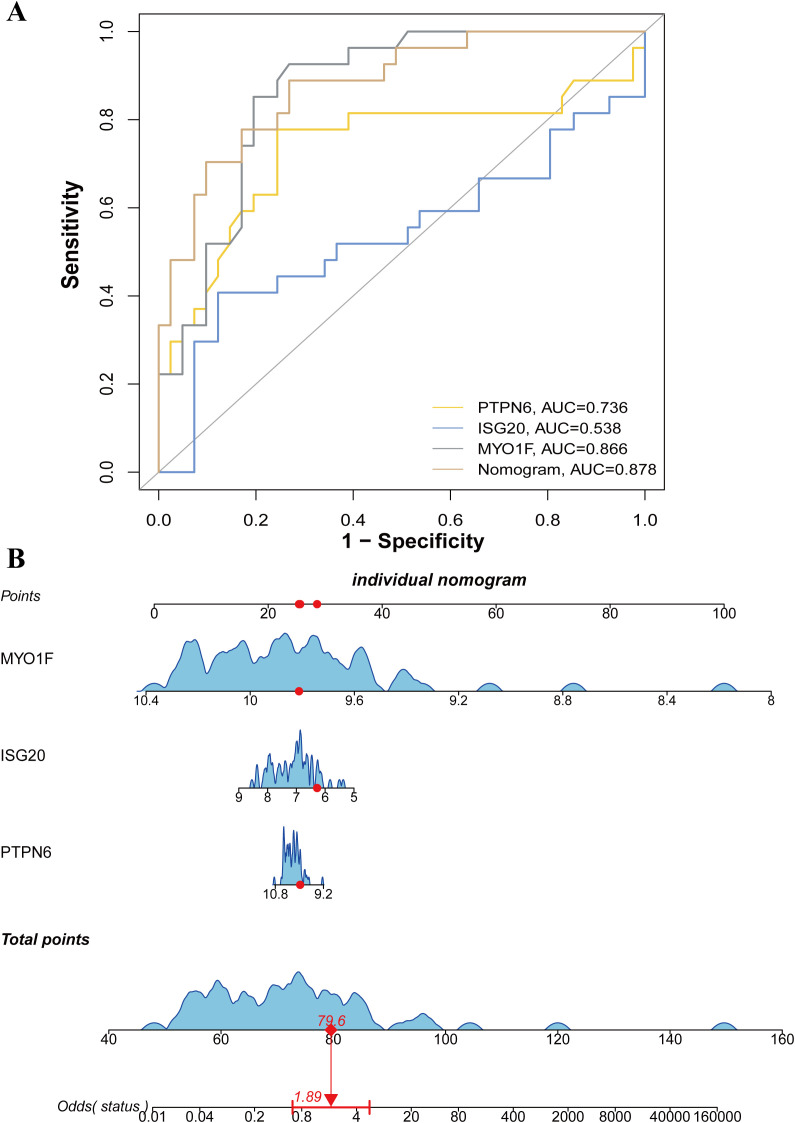
Validation of hub genes’ diagnostic performance. **(A)** ROC analysis plotting sensitivity (y-axis) against 1-specificity (x-axis), with area under the curve (AUC) values quantifying discriminative power. **(B)** Prognostic nomogram for predicting severe SALI risk: vertical lines extended from predictor-specific points on the “Points” scale are summed to determine a “Total Points” score, which is then projected to the “Probability” axis to estimate individual risk stratification.

Leveraging disease severity stratification data from general sepsis patients (GSE69063), rather than SALI-specific cohorts, we evaluated diagnostic efficacy of hub genes across clinical classifications. Given the absence of dedicated SALI severity datasets, we utilized this general sepsis cohort as a surrogate to assess the potential discriminative capacity of our hub genes in sepsis severity stratification, acknowledging that direct extrapolation to SALI prognosis requires further validation. ROC analysis demonstrated robust severity-predictive capacity for *MYO1F* (AUC = 0.866) and *PTPN6* (AUC = 0.736), both exceeding the diagnostic threshold (AUC>0.7). Conversely, *ISG20* exhibited limited discriminatory accuracy (AUC = 0.538), underscoring the concurrent dual-purpose utility of *PTPN6* and *MYO1F* in sepsis prognosis forecasting and disease grading (**Figure 8A**). The resultant diagnostic nomogram visually quantified clinical translatability of these hub genes for sepsis exacerbation risk stratification—revealing that patients with a cumulative gene score of 79.6 exhibit a 1.89-fold higher propensity for severe sepsis progression relative to their non-severe counterparts. We caution that this nomogram reflects general sepsis severity rather than SALI-specific outcomes. This polygenic integration strategy constitutes a pivotal decision-support framework for early identification of high-risk cohorts (**Figure 8B**).

### 3.6 Immune infiltration analysis of hub biomarkers

**Figure 9:**
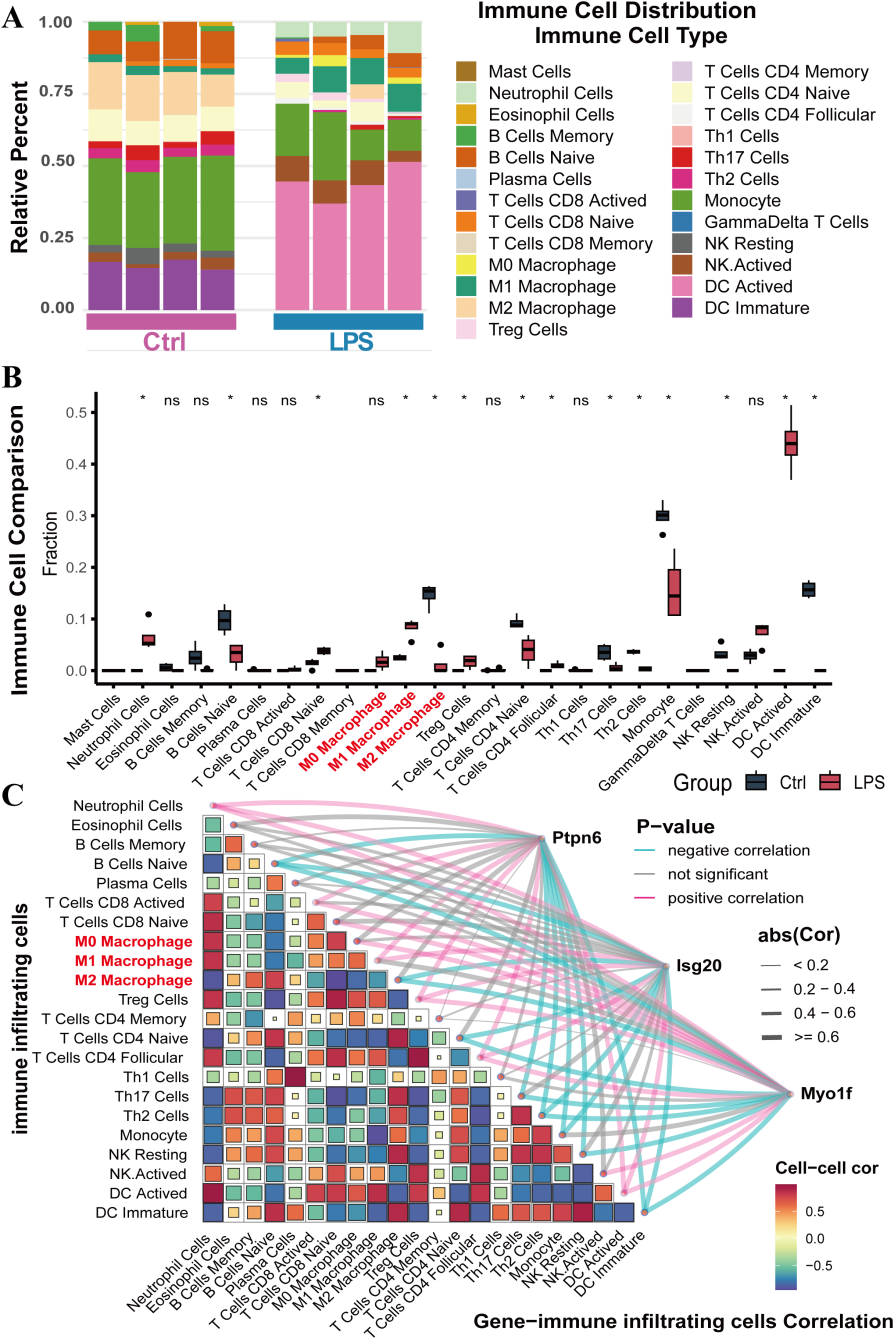
Immune infiltration analysis of murine lung tissue RNA-seq data. **(A)** Comparative profiling of immune cell population percentages between Ctrl and LPS groups. **(B)** Differential analysis of 22 immune cell subtypes across experimental conditions. **(C)** Correlation network demonstrating associations between hub genes (*PTPN6*, *ISG20*, *MYO1F*) and immune cells, with edge thickness reflecting correlation strength and color-coded connectors (segments/squares) indicating significance magnitude and directional relationships.

Employing the CIBERSORT algorithm coupled with murine immune signature matrices, we identified profound immune microenvironment restructuring in LPS-induced SALI murine models. Comparative analysis revealed significant enrichment of neutrophils, memory B cells, M1-polarized macrophages, and activated dendritic cells in pulmonary tissues of experimental versus Ctrl cohorts (**Figures 9A, B**; **Supplementary Table 7**). Higher-resolution immune correlation profiling delineated intricate molecular networks between core biomarkers and specific immune subsets: *PTPN6* exhibited robust positive correlations with neutrophils (r=0.89), M1 macrophages (r=0.81), and activated DCs (r=0.91); *ISG20* demonstrated synergistic positive regulatory associations with neutrophils (r=0.86), M1 macrophages (r=0.90), and mononuclear phagocytes (r=0.95); whereas *MYO1F* manifested not only positive associations with neutrophils (r=0.91) and regulatory T cells (r=0.86), but paradoxically exerted a potent antagonistic effect on M2 macrophages (r=-0.90) *(p<0.05)* (**Figure 9C**; **Supplementary Table 8**). These findings suggest inflammatory cell infiltration, macrophage polarization, and immunoregulatory cell activation as a potential regulatory axis in SALI pathogenesis. Further exploration of immune microenvironment dynamics suggests that candidate biomarkers—particularly *ISG20*, *MYO1F*, and *PTPN6*—may potentiate SALI pathogenesis in insomnia contexts through modulation of pro-inflammatory effector functions and immunoregulatory circuitry.

### 3.7 Hub gene correlation and single-gene GSEA analysis

**Figure 10:**
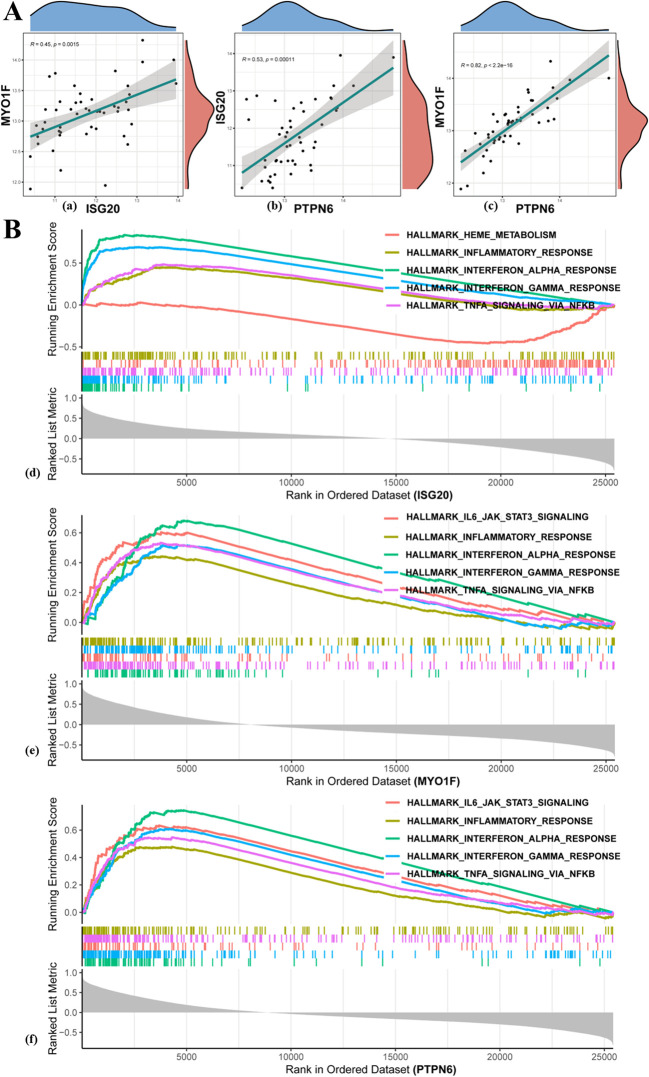
Correlation and GSEA analysis of hub genes. **(A)** Scatter plots demonstrating transcriptional correlation between **(a)** *ISG20* and *MYO1F*, **(b)** *PTPN6* and *ISG20*, and **(c)** *PTPN6* and *MYO1F*. **(B)** GSEA enrichment profiles revealing functional pathways associated with hub gene signatures.

Comprehensive investigation of intergenic expression relationships and functional characteristics revealed significant pairwise correlations: *ISG20*-*MYO1F* (R = 0.45, *p=0.0015*), *PTPN6*-*ISG20* (R = 0.53, *p=0.00011*), and *PTPN6*-*MYO1F* (R = 0.82, *p=2.2e-16*) all demonstrated robust positive co-expression, with *PTPN6*-*MYO1F* exhibiting the most pronounced synergistic expression pattern (**Figure 10A**). Guided by these strong associations, GSEA pathway enrichment delineated distinct functional signatures: *ISG20* predominantly orchestrates interferon pathways (IFN-α/γ), TNF-α signaling, heme metabolism, and inflammatory response cascades. In contrast, *MYO1F* and *PTPN6* coregulate the IL-6-JAK-STAT3 signaling axis while concurrently engaging interferon responses (IFN-α/γ), TNF-α pathways, and inflammatory mechanisms *(<0.05)* (**Figure 10B**; **Supplementary Table 9**). These findings suggest coordinated coregulatory involvement in immunological modulation through cytokine network regulation, thereby providing novel mechanistic insights into the concerted immunomodulatory functions of these hub genes at molecular interaction and pathway levels.

### 3.8 Single-cell analysis of hub gene expression distribution

**Figure 11:**
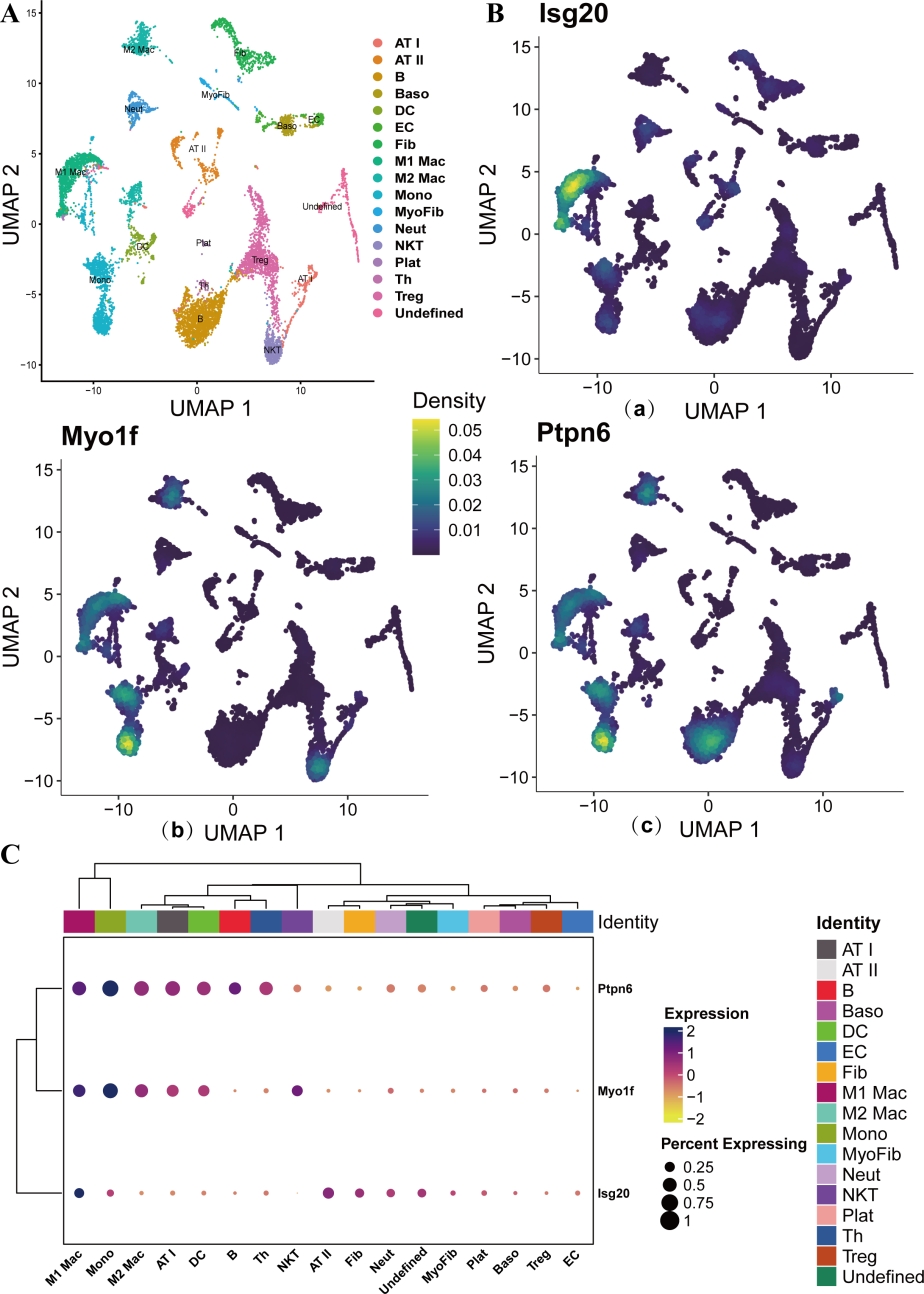
Distribution patterns of hub genes in human peripheral blood single-cell atlas. **(A)** UMAP clustering visualization of annotated single-cell transcriptomes from GSE276682 dataset. **(B)** Density distribution of hub genes, where gradient coloration transitions from deep blue (low expression) to vibrant green (high expression) across cell populations. **(C)** Dot plot representation of hub gene distribution: circle diameters proportional to expression prevalence within cell types (X-axis), while chromatic intensity scales with mean expression levels (cooler hues indicating higher abundance).

To elucidate the cellular distribution of hub genes within pulmonary microenvironments, we conducted systematic interrogation of single-cell transcriptomic data from the GSE276682 repository. Precise annotation of pulmonary cellular architectures into sixteen principal subtypes was achieved through canonical marker expression profiling (**Figure 11A**). Resolution at single-cell granularity delineated distinct spatial distribution patterns: *ISG20* exhibited characteristic compartmentalization within M1-polarized macrophages and alveolar type II epithelial cells (AT II); whereas *MYO1F* and *PTPN6* demonstrated significant enrichment across macrophage polarization continuum (M1/M2) and mononuclear phagocyte subsets (**Figures 11B, C**; **Supplementary Figure 2**). This preferential cellular distribution supports the mechanistic involvement of candidate hub genes in SALI pathogenesis through modulation of macrophage-mediated inflammatory cascades and immunoregulatory processes.

### 3.9 *In vivo* experimental results

**Figure 12:**
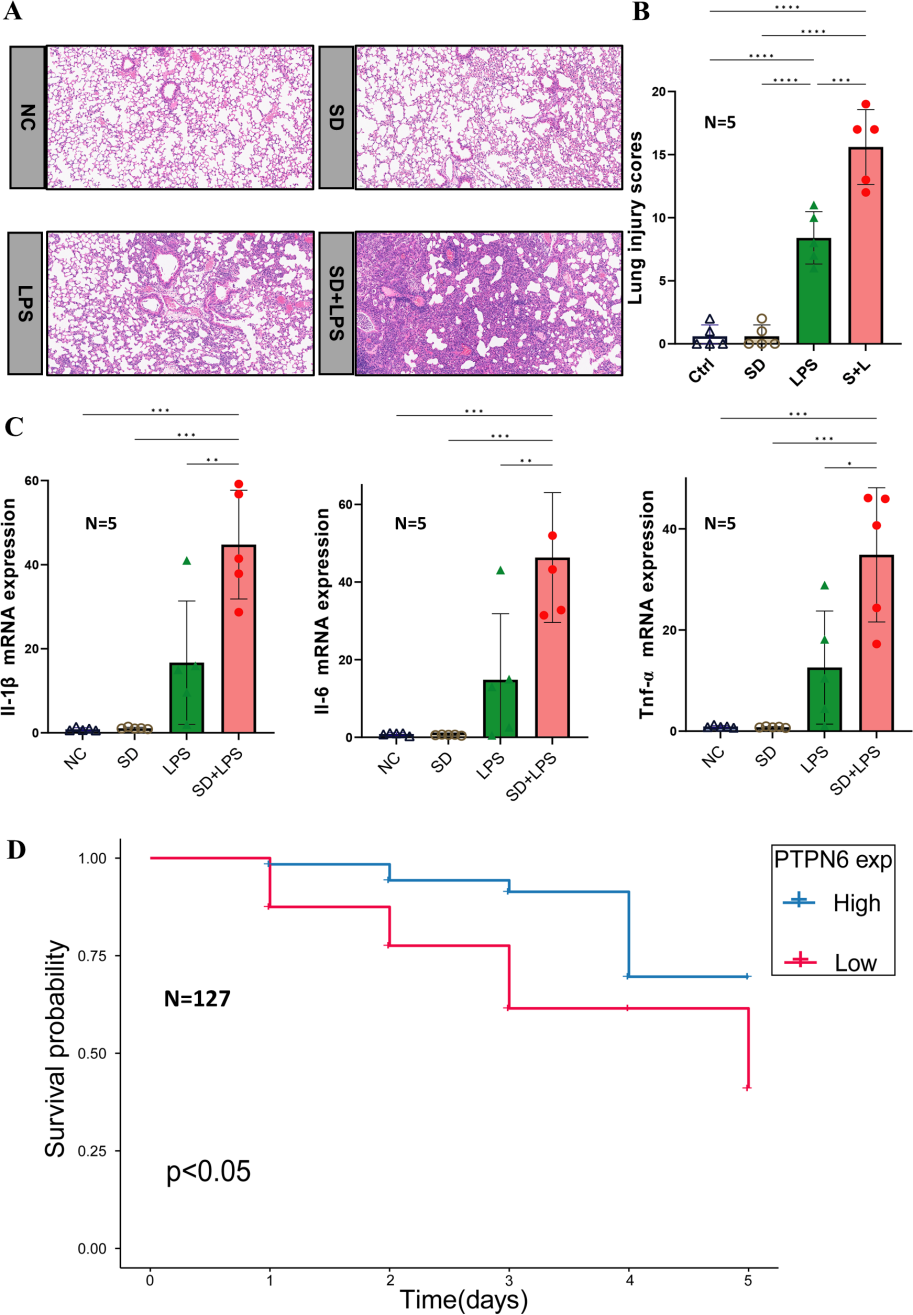
*In vivo* experimental results across NC, SD, LPS, and SD+LPS Groups. **(A)** Representative H&E-stained lung tissue sections showing histopathological alterations. **(B)** Quantitative assessment of lung injury severity scores based on H&E analysis. **(C)** mRNA expression levels of pro-inflammatory cytokines (Il-1β, Il-6, and Tnf-α) in lung tissues. **(D)** Kaplan-Meier survival analysis based on *PTPN6* expression status in the GSE54514 cohort. (*P < 0.05, **P < 0.01, ***P < 0.001, ****P < 0.0001).

We used a semi-quantitative scoring system for lung injury, including alveolar structure, inflammatory infiltration, intra-alveolar exudate, septal thickening, edema, vascular lesions, and fibrosis. Each paremeter was scored on a scale of 0 (normal) to 3 (severe), and the cumulative scores was categorized as mild (0~3), moderate (4~6), severe (7~9), or extremely severe (10~18) injury. Histopathological examination (**Figure 12A**; **Supplementary Figure 3**) revealed that NC and SD groups maintained normal pulmonary architecture without detectable inflammation, whereas the LPS group exhibited distinct pathologycal alteration, including disrupted alveolar structure, mild septal thickening, modest inflammatory infiltration, and slight exudate formation. The SD+LPS group demonstrated pronounced structural damage, including severe alveolar distortion, extensive septal thickening, dense inflammatory cell accumulation, and abundant intra-alveolar exudate. Consistent with these morphological changes, lung injury scoring (**Figure 12B**; **Supplementary Table 10**) showed comparable low severity in NC and SD groups, significantly elevated damage in LPS-treated animals (*p < 0.0001*), and maximally aggravated pathology in the SD+LPS cohort (*p < 0.001*). This progressive injury pattern correlated with inflammatory mediator levels (**Figure 12C**), where SD+LPS treatment specifically amplified Il-1β, Il-6, and Tnf-α production beyond measurements in LPS-challenged (*p < 0.05*) or NC (*p < 0.01*) groups, confirming synergistic pathological exacerbation by SD pretreatment. In the GSE54514 dataset, patients were stratified into low- and high-expression groups based on median *PTPN6* expression levels. Notably, the high-expression group demonstrated significantly superior overall survival compared to the low-expression group (*p=0.0073<0.05*) (**Figure 12D**).

### 3.10 PTPN6 overexpression suppresses LPS-induced macrophage activation *in vitro*

**Figure 13:**
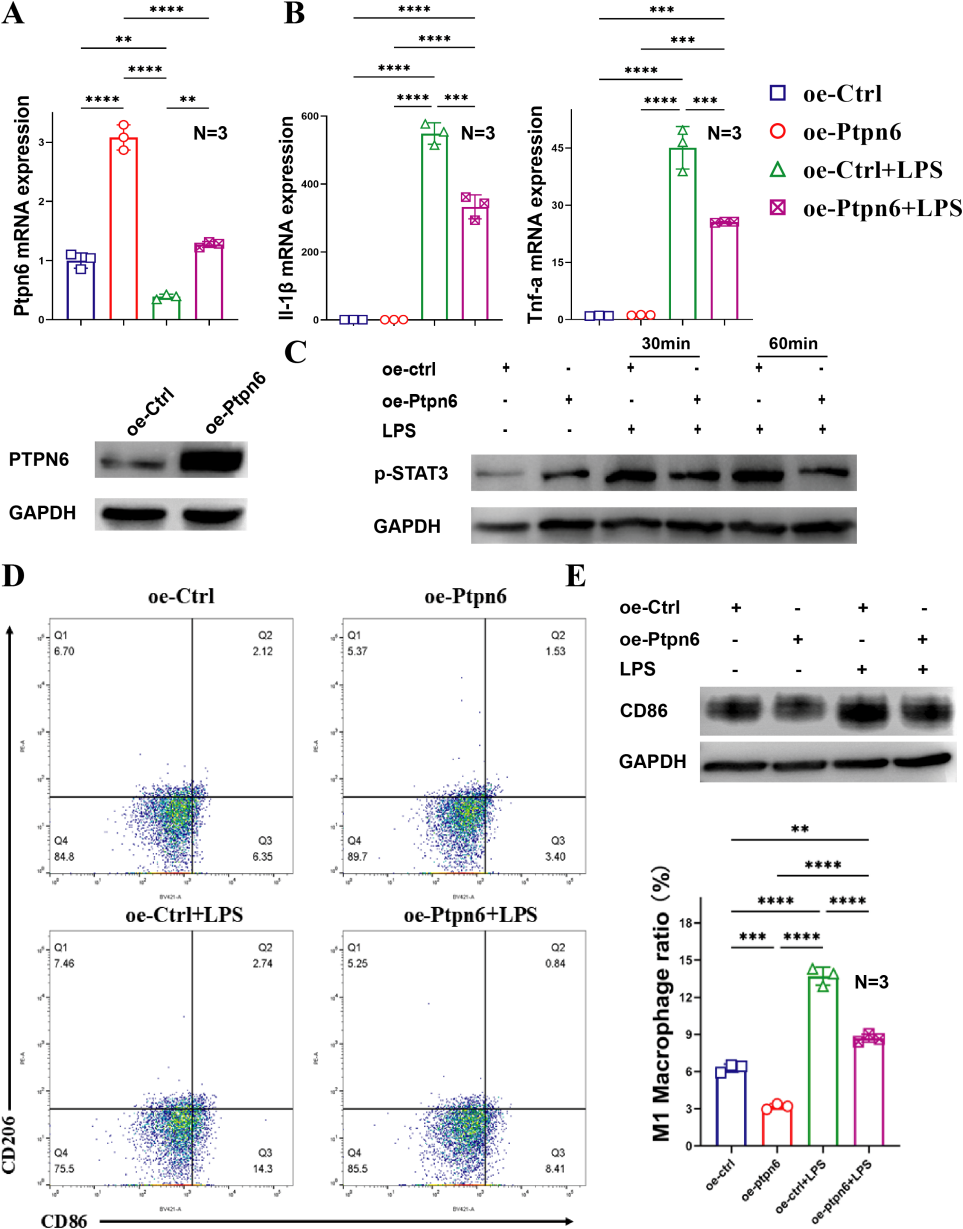
Functional validation of PTPN6 overexpression in RAW264.7 macrophages. **(A)** Validation of PTPN6 overexpression efficiency at both mRNA and protein levels in oe-Ptpn6 and oe-Ctrl cell lines. **(B)** Quantitative analysis of pro-inflammatory cytokine mRNA expression (Il-1β and Tnf-α) following LPS stimulation. **(C)** Western blot analysis of phosphorylated STAT3 (p-STAT3) protein abundance at 30 min and 60 min post-LPS stimulation. **(D)** Flow cytometric quantification of M1 macrophage (CD86^+^) subsets following LPS treatment (1 μg/ml, 12 h) in oe-Ctrl versus oe-Ptpn6 cells. **(E)** Representative Western blot of CD86 expression at 12 h post-LPS (1 μg/ml) stimulation. (*P < 0.05, **P < 0.01, ***P < 0.001, ****P < 0.0001).

To establish the causal role of PTPN6 in modulating macrophage inflammatory responses, we performed gain-of-function experiments in RAW264.7 macrophages. Lentiviral-mediated overexpression of Ptpn6 (oe-Ptpn6) achieved ~3-fold elevation in mRNA expression and robust protein induction compared to empty vector controls (oe-Ctrl) (**Figure 13A**). Upon LPS stimulation (1 μg/ml), oe-Ptpn6 cells exhibited markedly attenuated pro-inflammatory cytokine production, with significant reductions in Il-1b and Tnf-α mRNA levels compared to oe-Ctrl (**Figure 13B**). These findings demonstrate that elevated PTPN6 expression confers anti-inflammatory effects in activated macrophages. Given the predicted involvement of STAT3 signaling in PTPN6-mediated immune regulation, we examined STAT3 phosphorylation dynamics. Western blot analysis revealed that PTPN6 overexpression substantially suppressed LPS-induced p-STAT3 accumulation at both 30 and 60 minutes (**Figure 13C**), consistent with PTPN6’s established function as a negative regulator of this pathway.

To assess functional consequences on macrophage polarization, we quantified M1 (CD86^+^/CD206^-^) subset proportions by flow cytometry. Following 12-hour LPS exposure, oe-Ptpn6 cells showed significantly reduced M1 macrophage frequencies compared to oe-Ctrl (**Figure 13D**). This observation was corroborated by Western blot, demonstrating diminished CD86 protein expression in PTPN6-overexpressing cells (**Figure 13E**). Collectively, these data establish PTPN6 as a critical negative regulator of macrophage activation that operates through inhibition of STAT3-dependent M1 polarization and the suppression of pro-inflammatory cytokine transcription. These functional findings support a model wherein PTPN6 contributes to immune homeostasis in macrophages, with its downregulation potentially disrupting regulatory checkpoints implicated in SALI pathogenesis. Definitive demonstration of PTPN6-mediated protection in sleep-deprived animals awaits *in vivo* rescue studies.

## 4 Discussion

Sleep disturbance is increasingly recognized as a significant risk factor for systemic inflammation, with clinical and epidemiological studies consistently linking disordered sleep to elevated circulating pro-inflammatory cytokines that contribute to chronic inflammatory diseases (32, 33). At the molecular level, sleep deprivation amplifies cytokine secretion in monocyte-macrophage lineages and promotes constitutive activation of key signaling hubs, particularly STAT and NF-κB pathways. The identification of biomarkers that integrate insomnia and inflammatory responses holds clinical value, as such signatures may not only predict susceptibility to depression but also represent actionable therapeutic targets. Indeed, anti-inflammatory modulation of insomnia-associated pathways has been shown to attenuate depression incidence (34).

In critical care medicine, precise diagnosis relies on the ability to stratify patients based on pathophysiological characteristics, prognostic trajectories, and therapeutic responses (35). ARDS, the most severe form of ALI, continues to carry substantial mortality. This poor prognosis is compounded by significant heterogeneity within the current Berlin definition, which remains a limitation in both clinical practice and research (36). The syndromic nature of this classification increasingly appears misaligned with precision medicine objectives, highlighting the need for biomarker-driven, mechanism-based sub-phenotyping (37). Such biomarkers extend beyond traditional diagnostic categories by revealing actionable biological pathways and, their integration with multimodal data facilitates enhanced diagnostic precision (38).

In this study, we integrated evidence from MR analysis, histopathological assessment, and inflammatory cytokine profiling to demonstrate that insomnia exacerbates the pathological cascade of SALI. Despite this association, the mechanistic basis through which sleep disturbance predisposes to SALI remains inadequately investigated, and predictive biomarkers reflecting insomnia-associated susceptibility are lacking. Through integrated analysis of insomnia and SALI transcriptomes, we identified candidate diagnostic biomarkers and developed risk stratification protocols for SALI-prone populations with insomnia.

Methodologically, machine learning offers powerful capabilities for high-dimensional data processing and has demonstrated considerable potential in biomedical research (39). Supervised machine learning has been successfully implemented for constructing disease diagnostic frameworks, enabling effective identification of clinically relevant biomarkers (40, 41). Within machine learning algorithms integrated with electronic health record systems show promise for real-time ALI phenotyping (42, 43) and prior application of elastic net and support vector machine algorithms have identified diagnostic biomarkers with good discriminatory power (AUC 0.725–0.833) (44), opportunities remain to further enhance predictive accuracy.

Using WGCNA and machine learning, we identified *ISG20*, *MYO1F*, and *PTPN6* as hub genes potentially linking insomnia and SALI. Interpretable SHAP modeling, complemented by stratified analyses of SALI cohorts, clarified the protective role of *PTPN6* and reinforced its combined diagnostic utility with *MYO1F*. These findings provide a molecular basis for proactive risk stratification in patients with insomnia and suggest novel targets for precision medicine in SALI.

Immune dysregulation represents a central pathogenic mechanism in ALI, with substantial evidence indicating that cytokine storm constitutes a key driver of progression from sepsis to ALI (45, 46). Recent therapeutic advances have established immunomodulatory interventions, including glucocorticoids and IL-6 monoclonal antibodies, as cornerstone strategies for managing ALI (47). Our immune infiltration profiling confirms that maladaptive immune signaling operates as a critical determinant in SALI progression, wherein aberrant infiltration of macrophages, neutrophils, and B-lineage lymphocytes mechanistically associates with exacerbated lung injury.

Macrophages serve as pivotal orchestrators in ALI and pulmonary infections, governing inflammation initiation, progression, and resolution (48).However, substantial heterogeneity among macrophage subpopulations and their context-dependent functional differences across disease phases have complicated the elucidation of precise regulatory networks in ALI pathogenesis (49, 50). Previous studies have demonstrated that macrophage-intrinsic factors can significantly influence ALI progression through modulation of autophagic flux and polarization states (51), while therapeutic interventions targeting macrophage reprogramming show promise for attenuating pulmonary inflammation (52).

Among the biomarkers identified in our study, *PTPN6* emerged as a particularly significant diagnostic and prognostic determinant. Substantial evidence underscores the non-redundant role of *PTPN6* in restraining immune activation. Genetic ablation studies have established a causal link between *PTPN6* deficiency and severe inflammatory (53, 54). Moreover, cell-specific deletions in microglia and neutrophils have further revealed the critical involvement of Syk and MAPK signaling pathway (55, 56). Corroborating this, *PTPN6* overexpression ameliorates pulmonary inflammation (57), while its neutrophil-specific deletion exacerbates ALI (58). Beyond pulmonary contexts, *PTPN6* inhibition in acute myeloid leukemia (AML) models disrupts two pivotal oncogenic cascades (JAK/STAT and PI3K/AKT), reducing cell viability and promoting differentiation (59).

Our study extends these findings to insomnia-associated SALI, with PTPN6 as a candidate diagnostic biomarker. ScRNA-seq showed its predominant macrophage expression, suggesting that *PTPN6* may confer pulmonary protection by mitigating macrophage-driven hyperinflammation and oxidative stress. Integrating these findings with our GSEA results, we hypothesize that *PTPN6* may modulate macrophage polarization via JAK/STAT pathway regulation in SALI. Elevated *PTPN6* may recalibrate macrophage phenotypic toward a protective state, whereas sleep disturbance-induced *PTPN6* downregulation could disrupt this balance, aggravating SALI. The functional validation experiments demonstrate that PTPN6 overexpression in RAW264.7 macrophages significantly attenuates LPS-induced pro-inflammatory cytokine production (Il-1β, Tnf-α), suppresses STAT3 phosphorylation, and inhibits M1 macrophage polarization (**Figure 13**). These findings indicate PTPN6 as a cell-autonomous negative regulator of macrophage-mediated inflammation and provide direct experimental evidence supporting its protective role in SALI. This insight provides a novel theoretical framework for understanding sleep deprivation-associated SALI pathogenesis.

## 5 Study limitations and methodological considerations

We acknowledge important constraints in integrating PBMC-derived insomnia signatures with lung-focused SALI datasets. First, PBMCs represent the most accessible peripheral immune surrogate for systemic inflammatory states. Insomnia-associated immune dysregulation (e.g., pro-inflammatory cytokine signaling, HPA axis perturbation) is reliably detectable in circulating cells and reflects systemic priming that predisposes to organ-specific vulnerability. While direct lung datasets are unavailable, PBMC signatures serve as a clinically relevant bridge between sleep disturbance and pulmonary susceptibility.

Second, ortholog mapping employed stringent g:Profiler filtering, retaining only high-confidence one-to-one orthologs (1,163 of 1,294 genes; 89.9% conversion rate). PTPN6, ISG20, and MYO1F all maintained unambiguous orthologs (Ptpn6, Isg20, Myo1f), with 131 ambiguous mappings excluded.

Third, PTPN6 robustness is supported by multiple validation layers independent of species translation: (i) consistent emergence across RF, SVM, and KNN algorithms in human SALI datasets with stable ROC performance; (ii) macrophage enrichment confirmed in human PBMC scRNA-seq (GSE276682), aligning with murine lung profiling; and (iii) conserved JAK/STAT3 regulatory function across species (59, 60). These convergent lines mitigate concerns regarding mapping sensitivity or species-specific artifacts.

## 6 Conclusion

This research combines multi-omics analysis to clarify the mechanism via which insomnia intensifies SALI and to find possible biomarkers. WGCNA identified 1,294 genes co-perturbed under both settings, which are enriched in immunological control, phagocytosis, and epigenetic modification. Pathway analysis further indicated the involvement of Fcr receptor-mediated phagocytosis, B cell receptor signaling, and chemokine activity. Machine learning techniques (RF, LASSO, and SVM) applied to 102 differentially expressed genes identified three hub genes (*ISG20*, *MYO1F*, *PTPN6*), with *PTPN6* exhibiting greater diagnostic efficacy. Immunoinfiltration and single-cell RNA sequencing revealed that *PTPN6* regulates macrophage polarization via the JAK-STAT pathway, and its downregulation resulting from sleep deprivation disturbs pulmonary immune homeostasis. Gain-of-function experiments demonstrated that elevated PTPN6 attenuates LPS-induced inflammatory responses and M1 macrophage polarization, establishing causality between PTPN6 expression and macrophage inflammatory output. Despite the constraints of sample size, our results identify *PTPN6* as a crucial regulator in insomnia-related SALI and provide the groundwork for future diagnostic and therapeutic approaches.

## Data Availability

The datasets presented in this study can be found in online repositories. The names of the repository/repositories and accession number(s) can be found in the article/**Supplementary Material**.

## Supplementary material

Supplementary Figure 1 WGCNA of SALI and insomnia datasets. **(A, B)** Soft-thresholding selection and cluster tendency analysis for the SALI dataset. **(C, D)** Soft-thresholding selection and cluster tendency analysis for the insomnia dataset.

Supplementary Figure 2 Expression profiles of hub genes (*PTPN6*, *ISG20*, and *MYO1F*) across distinct cell types in single-cell RNA sequencing data.

Supplementary Figure 3 Representative H&E-stained lung tissue sections from experimental mouse groups: NC, SD, LPS, and SD+LPS.

Supplementary Figure 4 Dot plot of marker genes used for annotating cell types in the single-cell dataset.

Supplementary Table 1 Effect sizes of instrumental SNPs used in MR analysis for exposure-outcome associations.

Supplementary Table 2 Primary results of the MR analysis.

Supplementary Table 3 Significantly co-expressed gene modules identified in WGCNA, with correlation heatmaps indicating key interactions.

Supplementary Table 4 GO enrichment analysis results.

Supplementary Table 5 KEGG pathway enrichment results.

Supplementary Table 6 Feature importance rankings of hub genes in machine learning models (KNN, SVM, and Random Forest).

Supplementary Table 7 Proportions of immune cell subtypes estimated via deconvolution analysis.

Supplementary Table 8 Correlation analysis between immune cell infiltration levels and hub gene expression.

Supplementary Table 9 GSEA results for hub genes.

Supplementary Table 10 Histopathological lung injury scoring.

Supplementary Table 11 A mouse-adapted immune signature matrix was constructed based on murine immune transcriptional profiles.

Supplementary Table 12 Marker gene information for each annotated cell type in the single-cell dataset.

Supplementary Table 13 AUC 95% confidence intervals.

## Glossary

SALI: sepsis induced acute lung injury
MR: Mendelian randomization
WGCNA: Weighted gene co-expression network analysis
RF: Random Forest
SVM: Support Vector Machine
KNN: K-Nearest Neighbors
MODS: multiple organ dysfunction syndrome
PICU: pediatric intensive care unit
ALI: Acute lung injury
ARDS: acute respiratory distress syndrome
SNS: sympathetic nervous system
HPA: hypothalamic-pituitary-adrenal
CRP: C-reactive protein
GWAS: Genome-wide association study
SNPs: single nucleotide polymorphisms
IVW: Inverse variance weighted
PBMCs: Peripheral blood mononuclear cells
scRNA-seq: single-cell RNA sequencing
kNN: k-nearest neighbors
PCA: Principal component analysis
TOM: topological overlap matrix
GO: Gene ontology
BP: biological process
CC: cell component
MF: molecular function
KEGG: Kyoto Encyclopedia of Genes and Genomes
DEGs: Differentially expressed genes
PPI: protein-protein interaction
GLM: Generalized linear model
GBM: Gradient boosting machine
NNET: Neural Network with Single Hidden Layer
DT: Decision tree
ROC: Receiver operating characteristic
PRC: Precision-recall curves
OOB error: Out-of-Bag error
RMSE: Root Mean Squared Error
RFE: Recursive feature elimination
XGBoost: eXtreme Gradient Boosting
GSEA: Gene Set Enrichment Analysis
IACUC: Institutional Animal Care and Use Committee
LPS: Lipopolysaccharide
H&E: hematoxylin and eosin
FDR: False discovery rate
MEs: module eigengenes
MM: Module membership
AUC: Area under the curve
AT II: Alveolar type II epithelial cells
CTD: Comparative toxicogenomics database
FRPQ: Fei Re Pu Qing Powder
PTKs: Protein tyrosine kinases
PTPs: Protein tyrosine phosphatases
ITIMs: immunoreceptor tyrosine-based inhibitory motifs
NET: Neutrophil extracellular trap
AML: acute myeloid leukemia;
