# Supplementary material for: A machine learning integrated multi-omics framework for risk prediction and target discovery in insomnia aggravated sepsis induced acute lung injury
Source: Front Immunol. 2026 Jun 1;17:1721749. doi: 10.3389/fimmu.2026.1721749 (PMC13265312; doi:10.3389/fimmu.2026.1721749)
Supplement: Supplementary Figure 1 — WGCNA of SALI and insomnia datasets. (A, B) Soft-thresholding selection and cluster tendency analysis for the SALI dataset. (C, D) Soft-thresholding selection and cluster tendency analysis for the insomnia dataset. [file DataSheet1.pdf]

Supplementary Figure 1

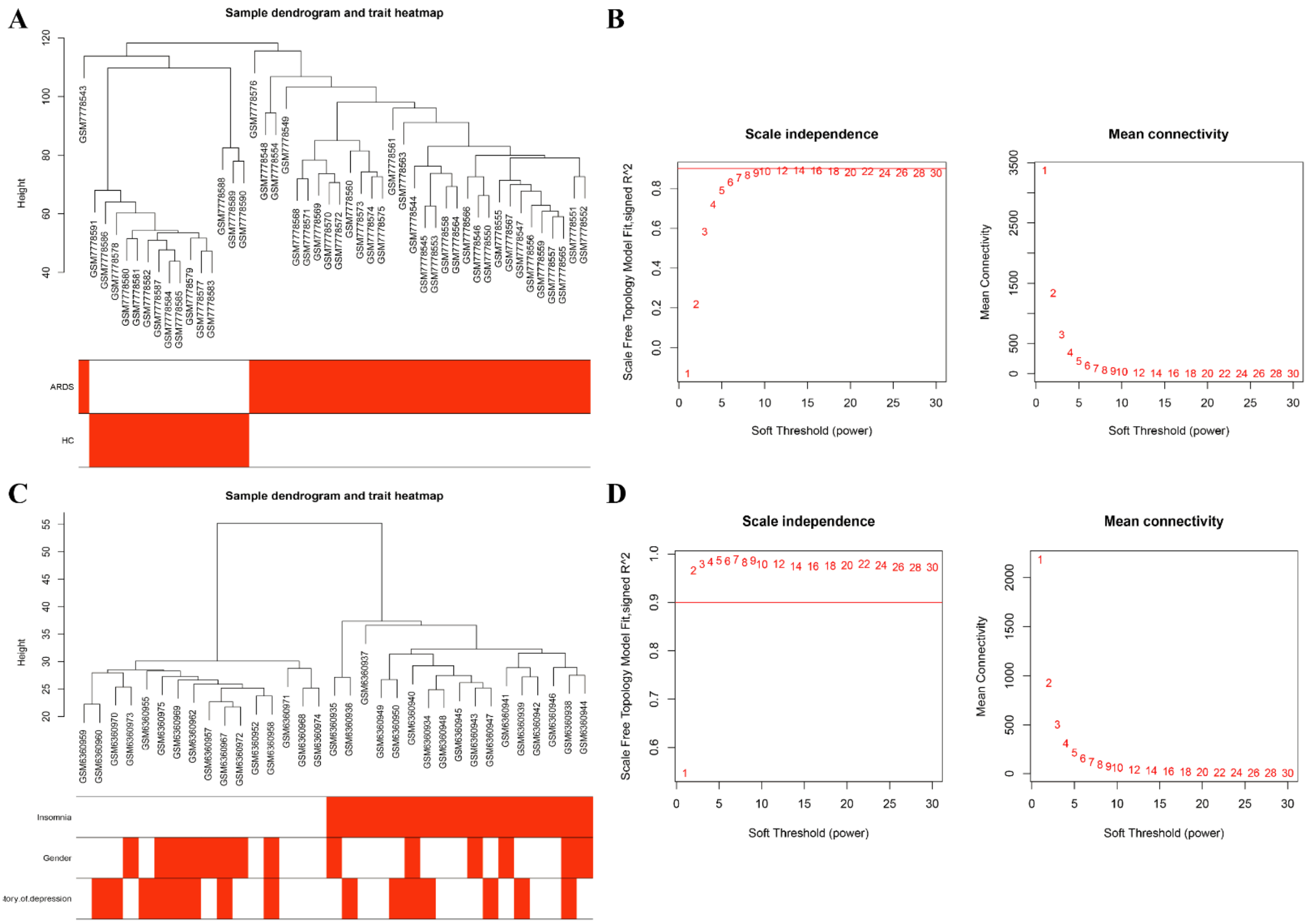

Supplementary Figure 2

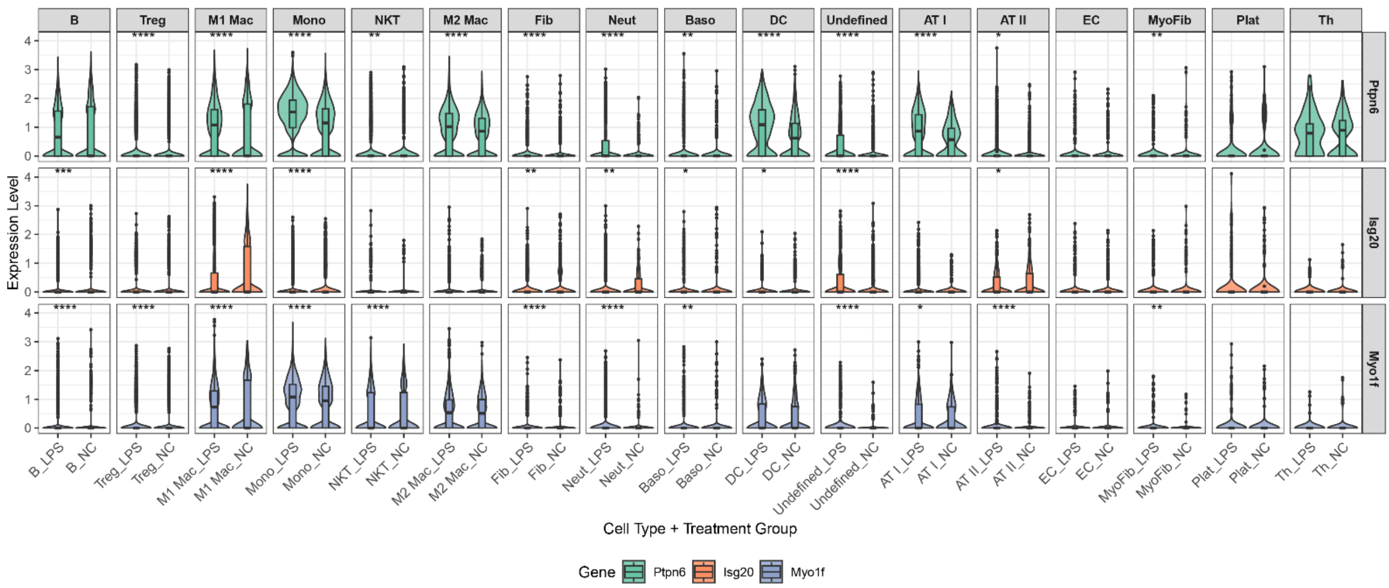

Supplementary Figure 3

NC group

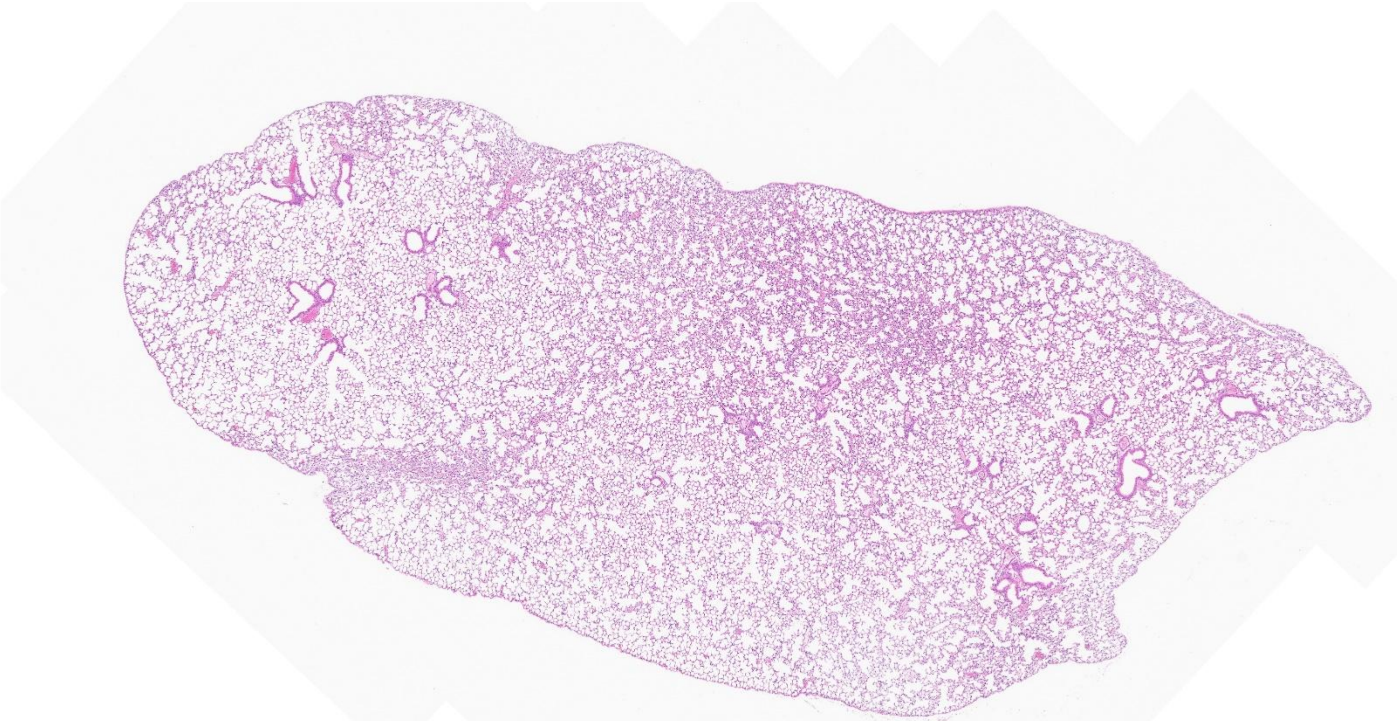

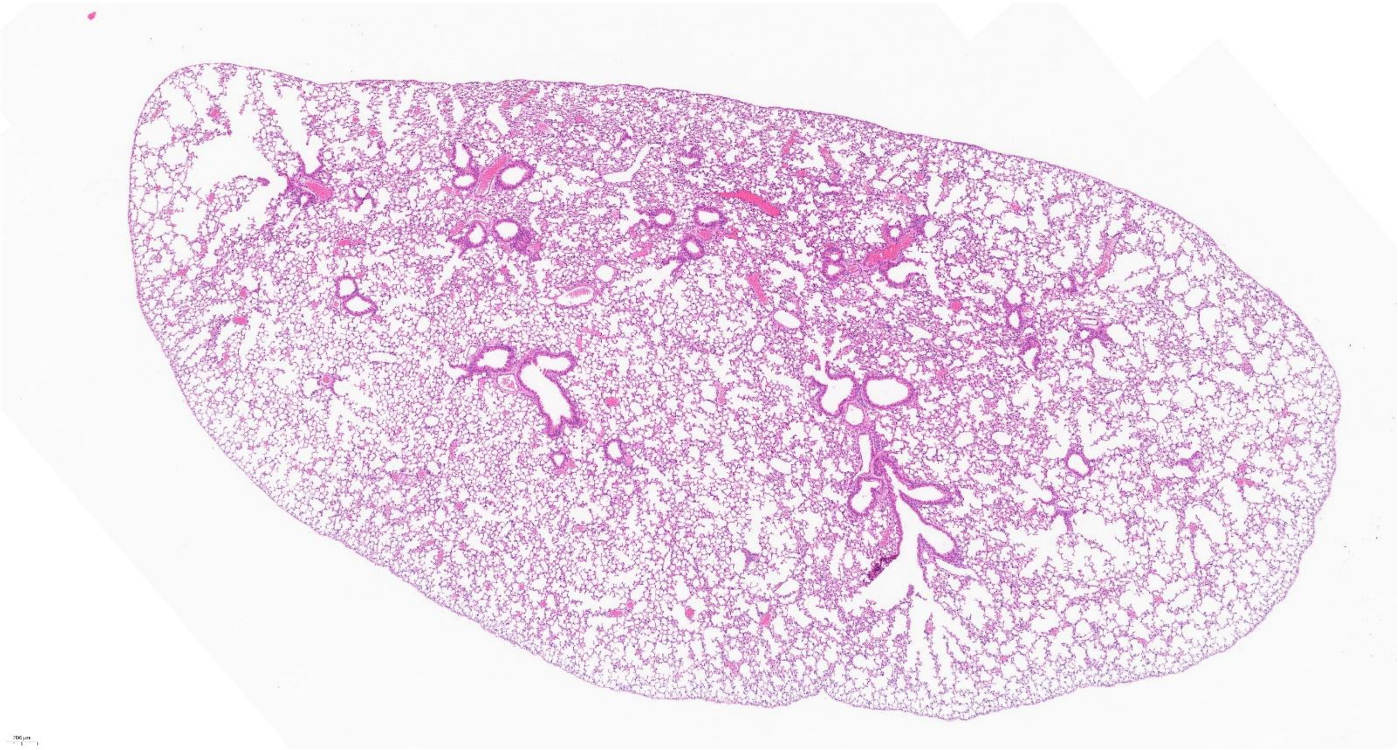

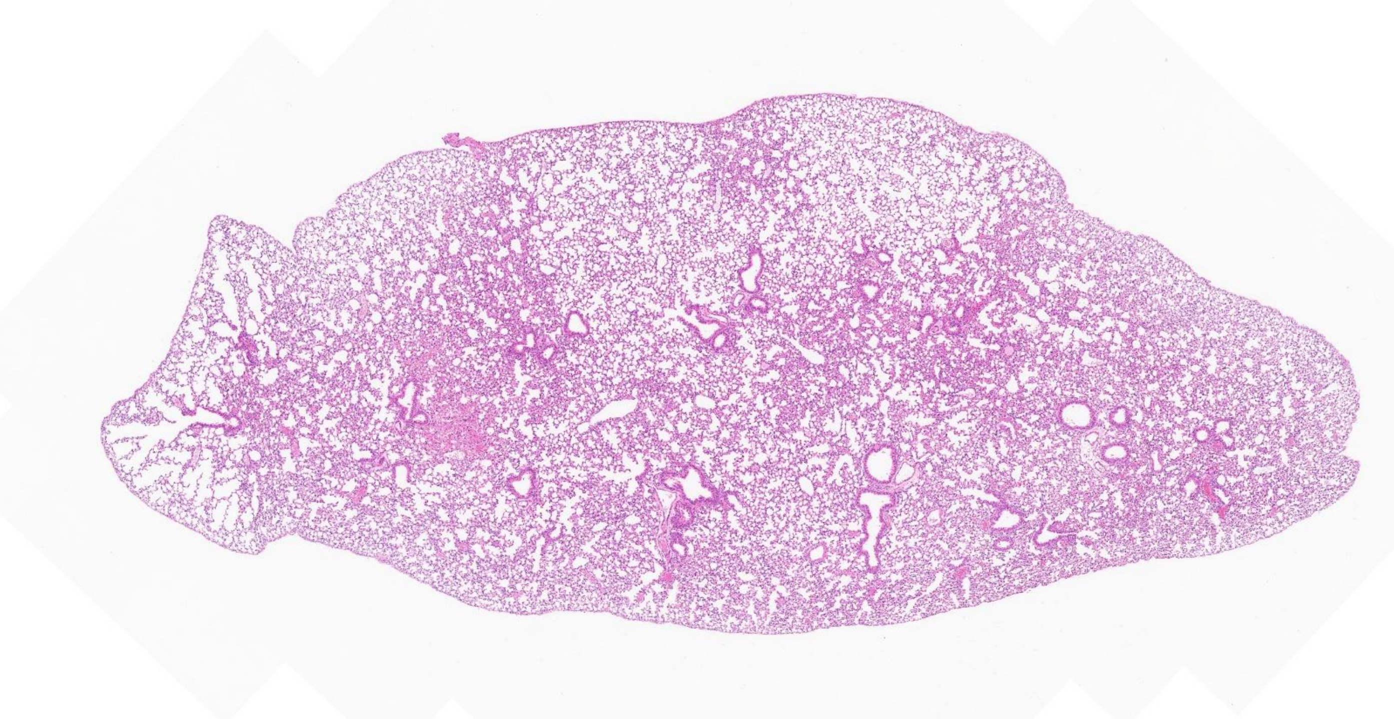

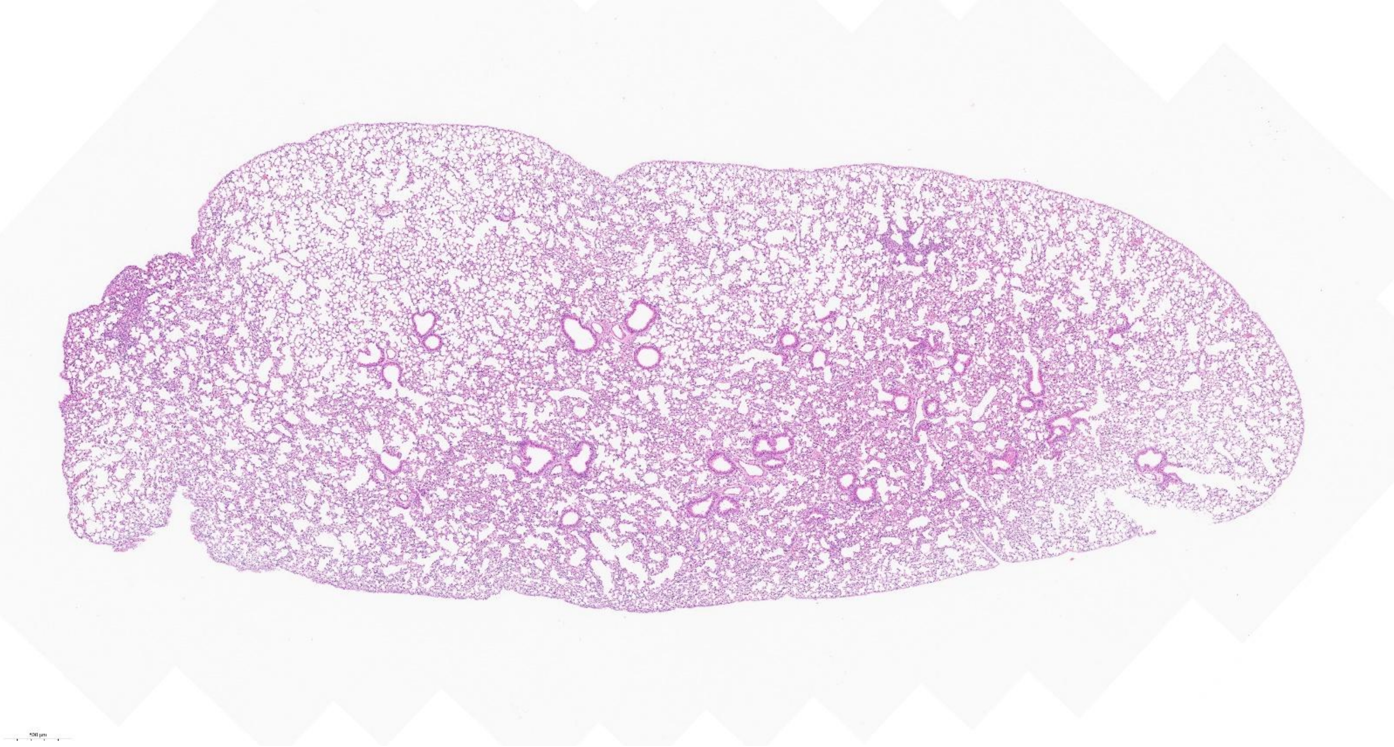

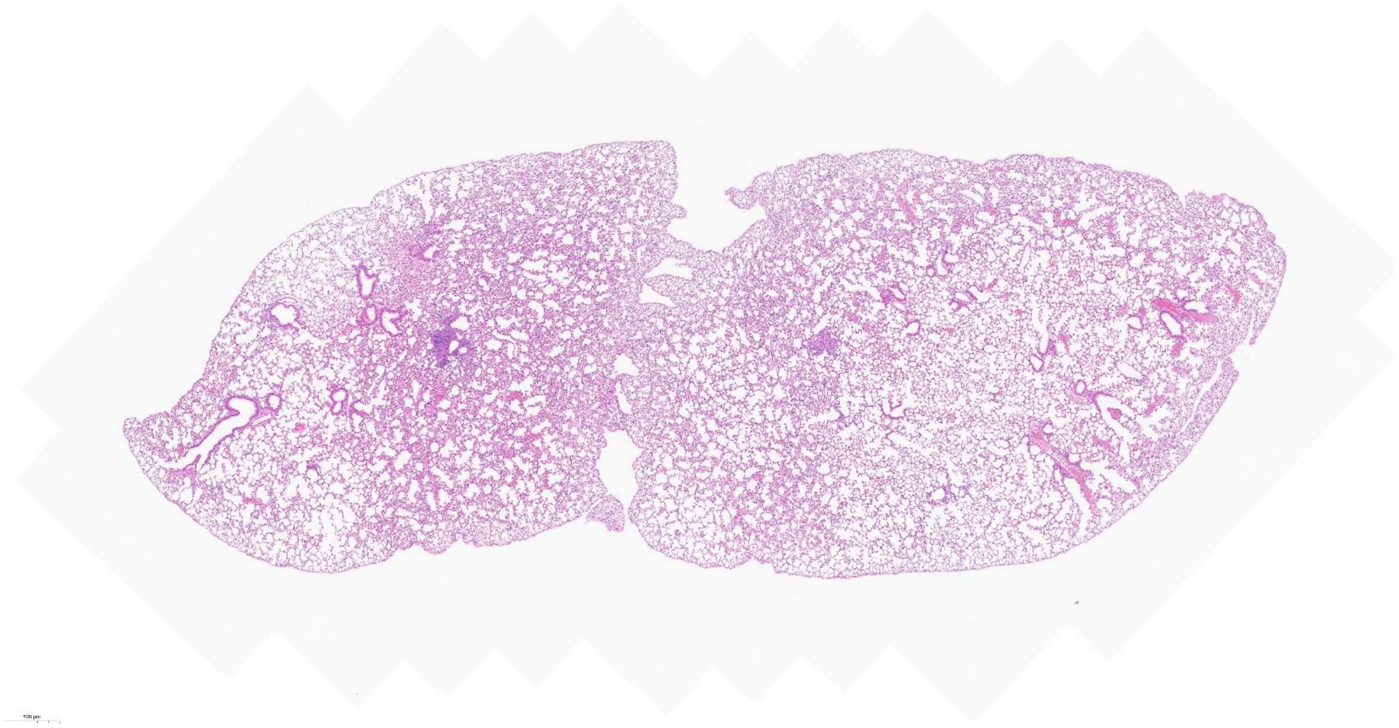

SD group

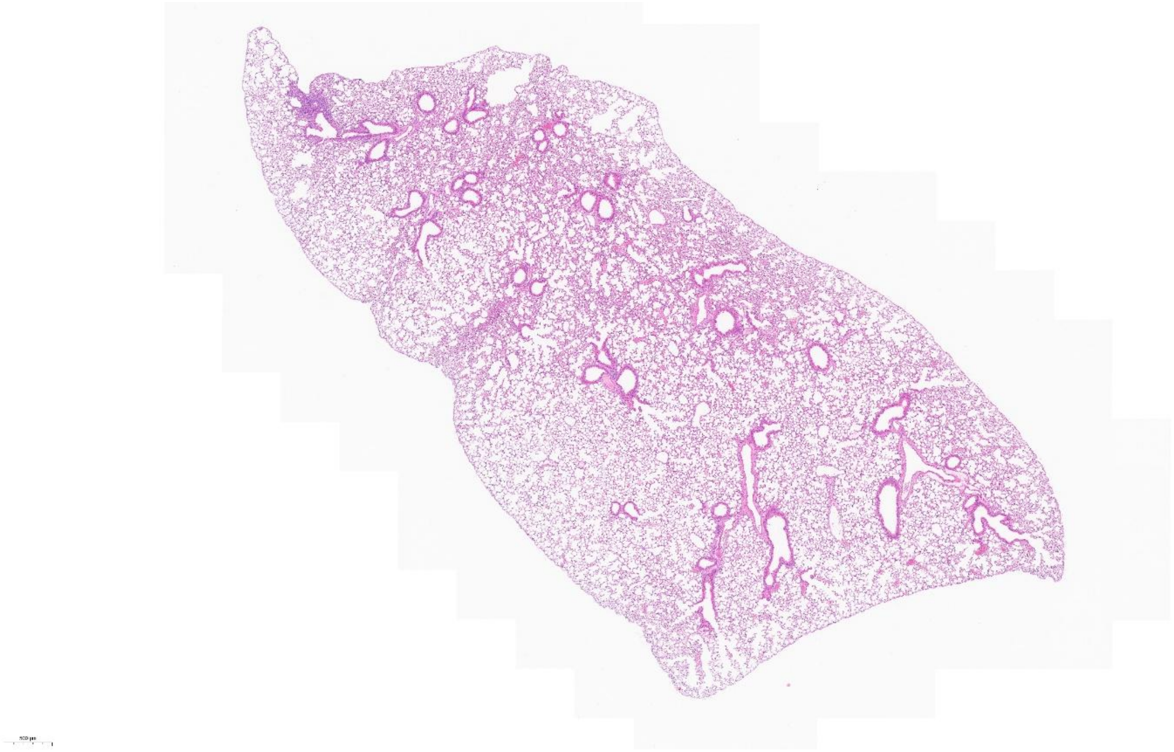

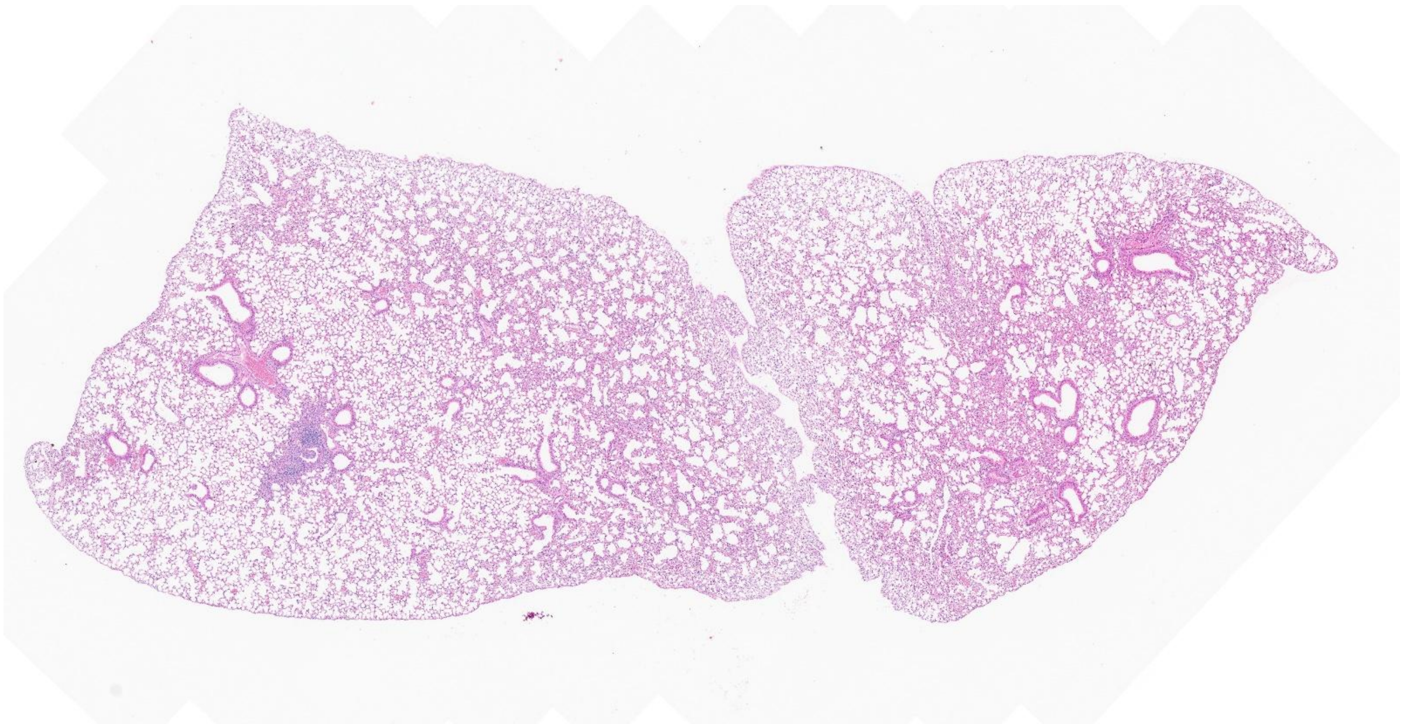
500 µm

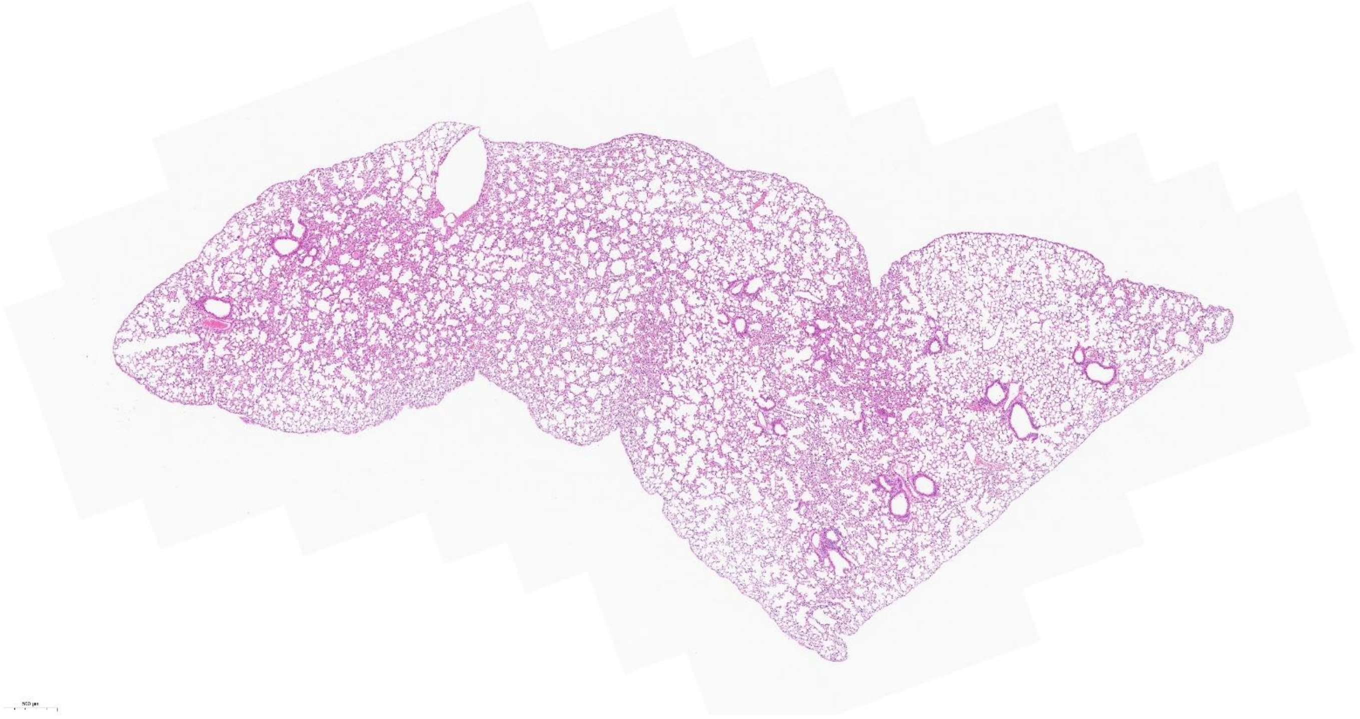
500 µm

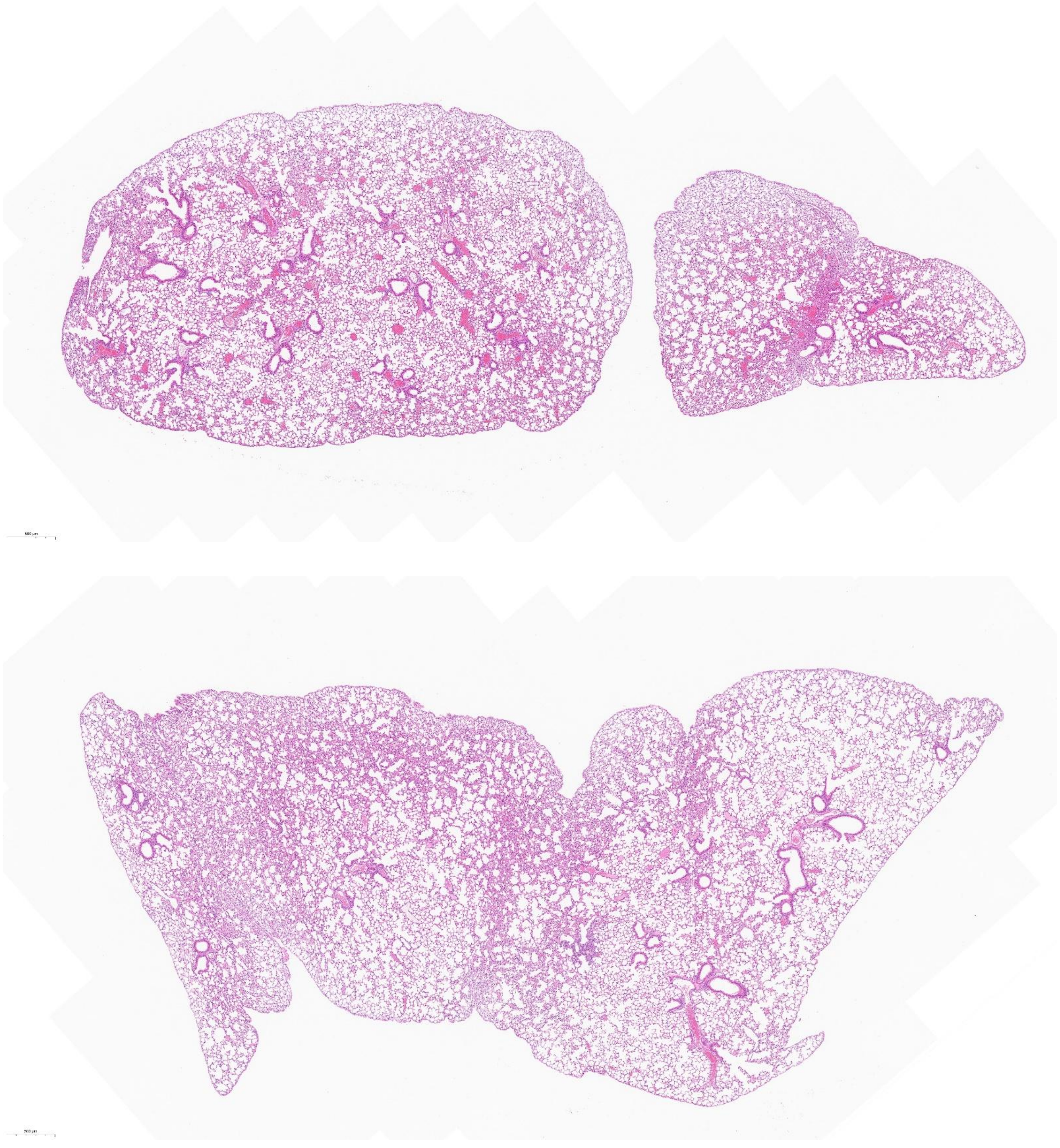

LPS group

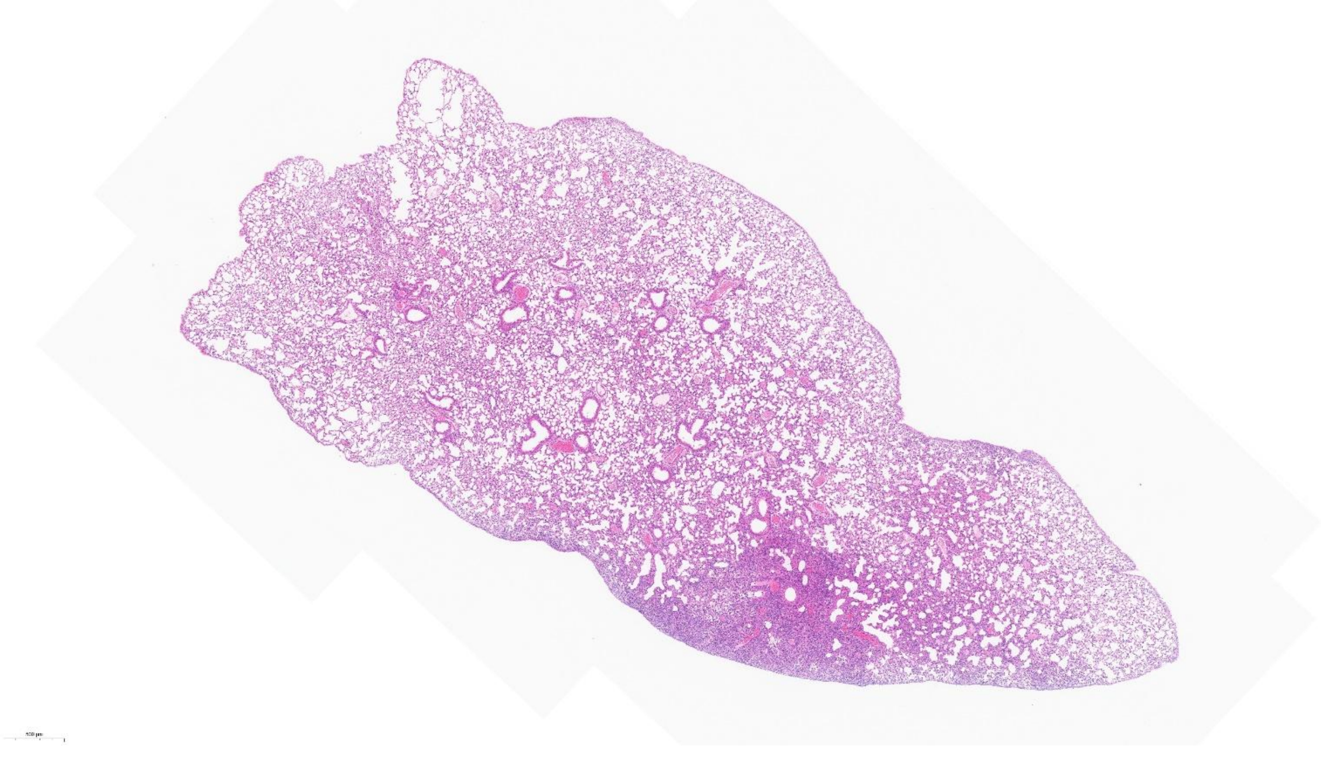

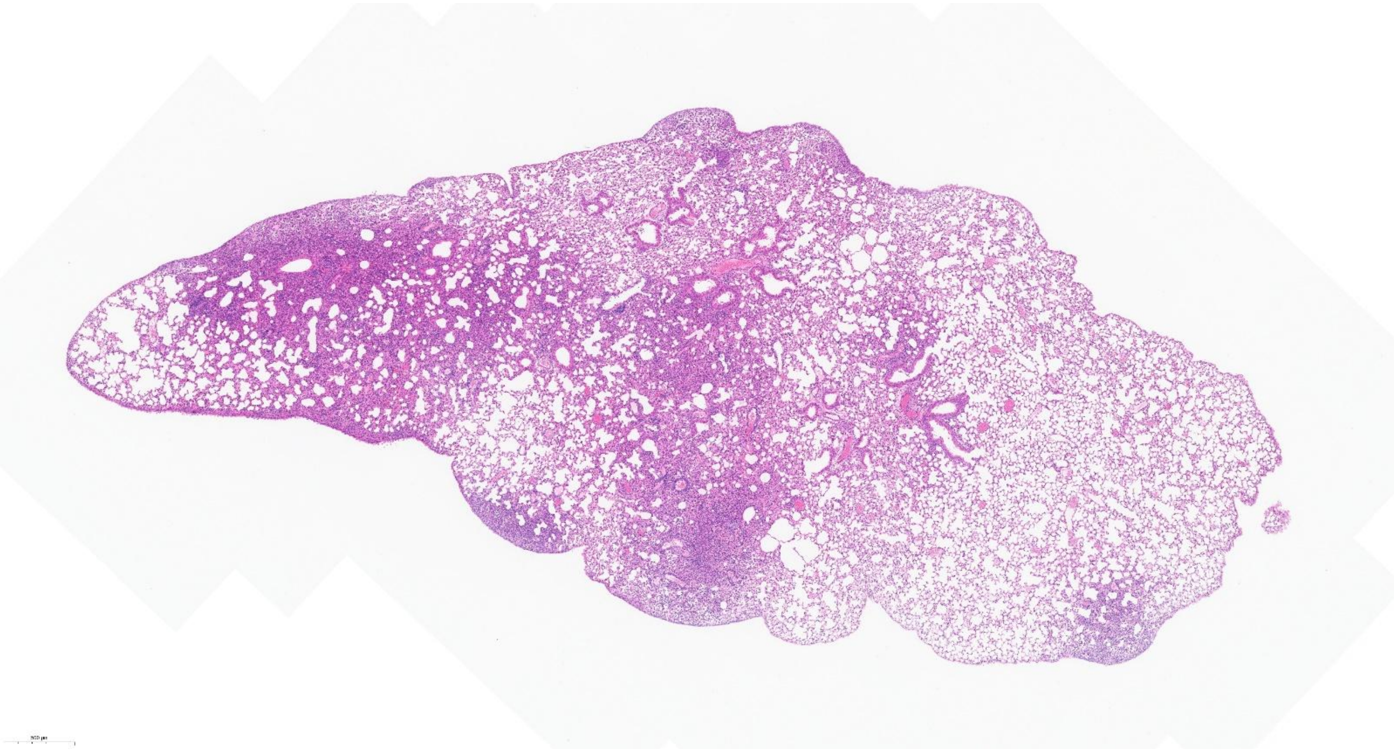

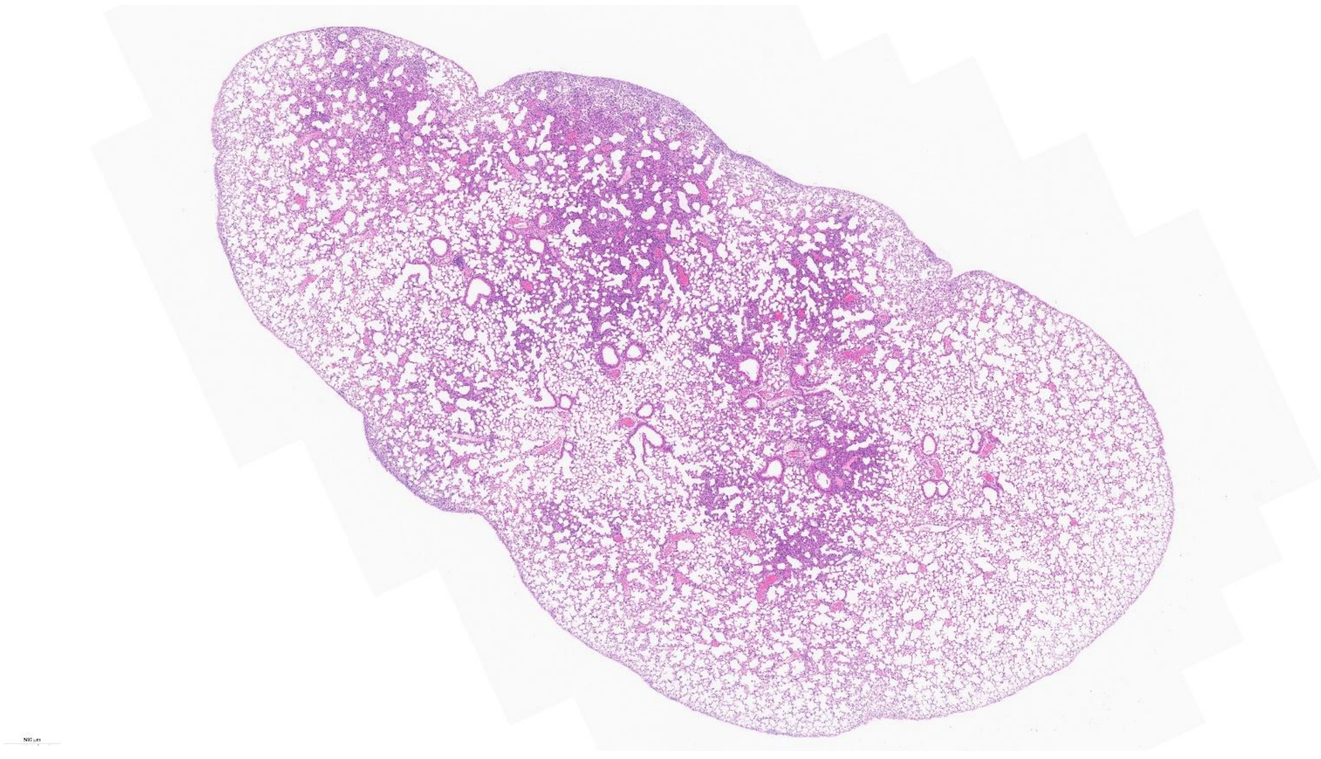
500 µm

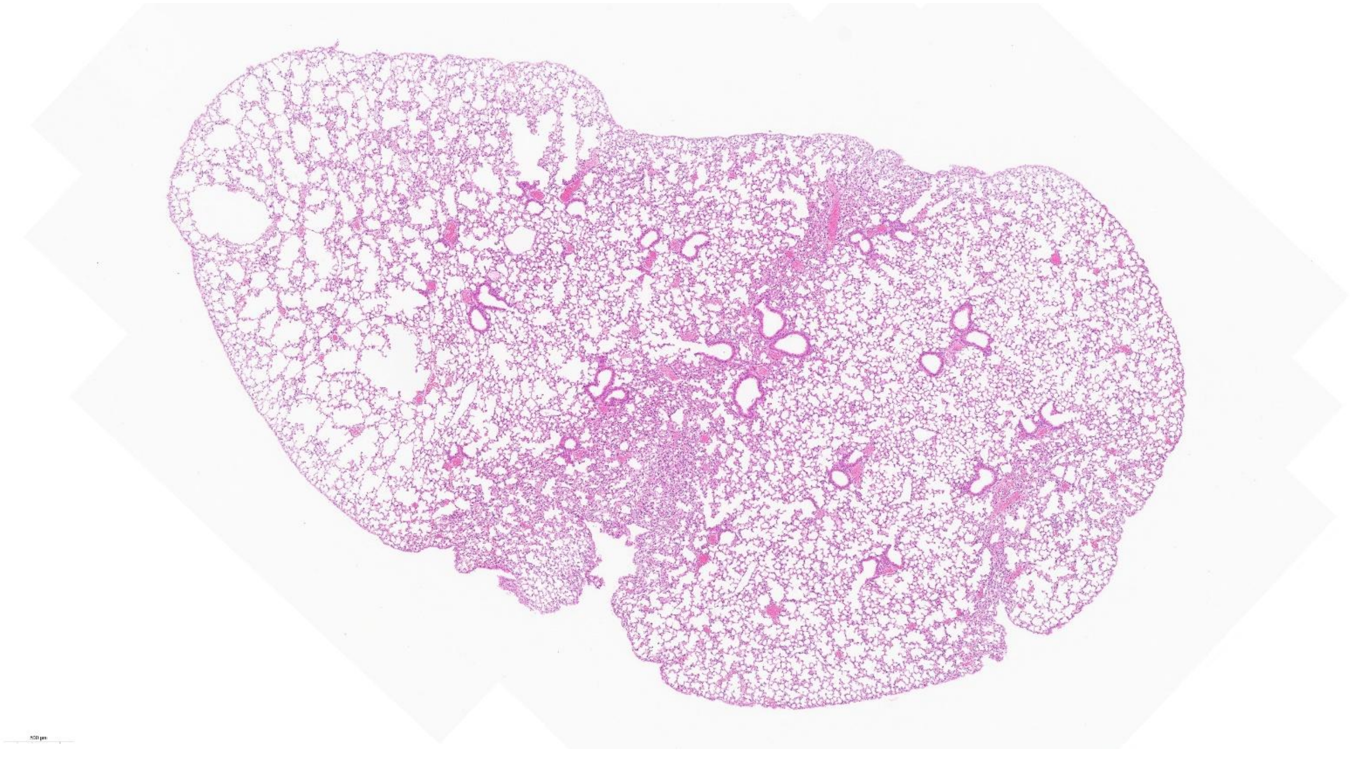

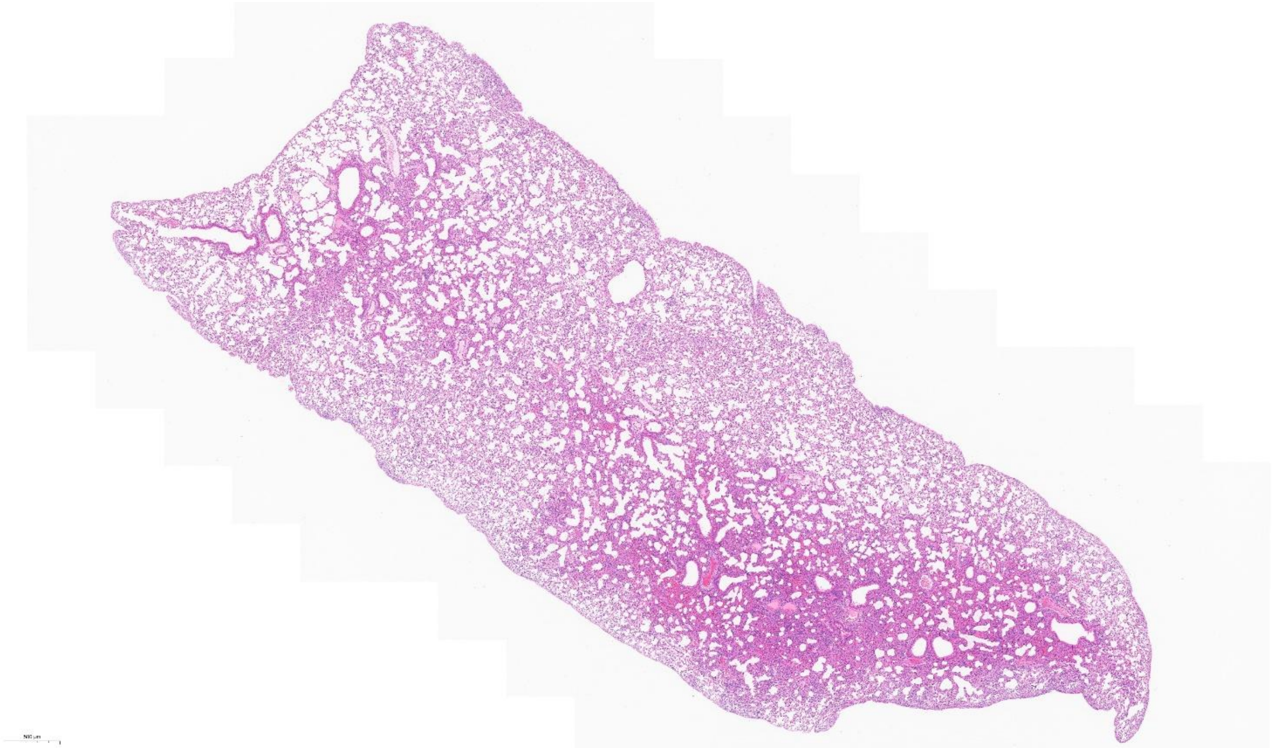

SD+LPS group

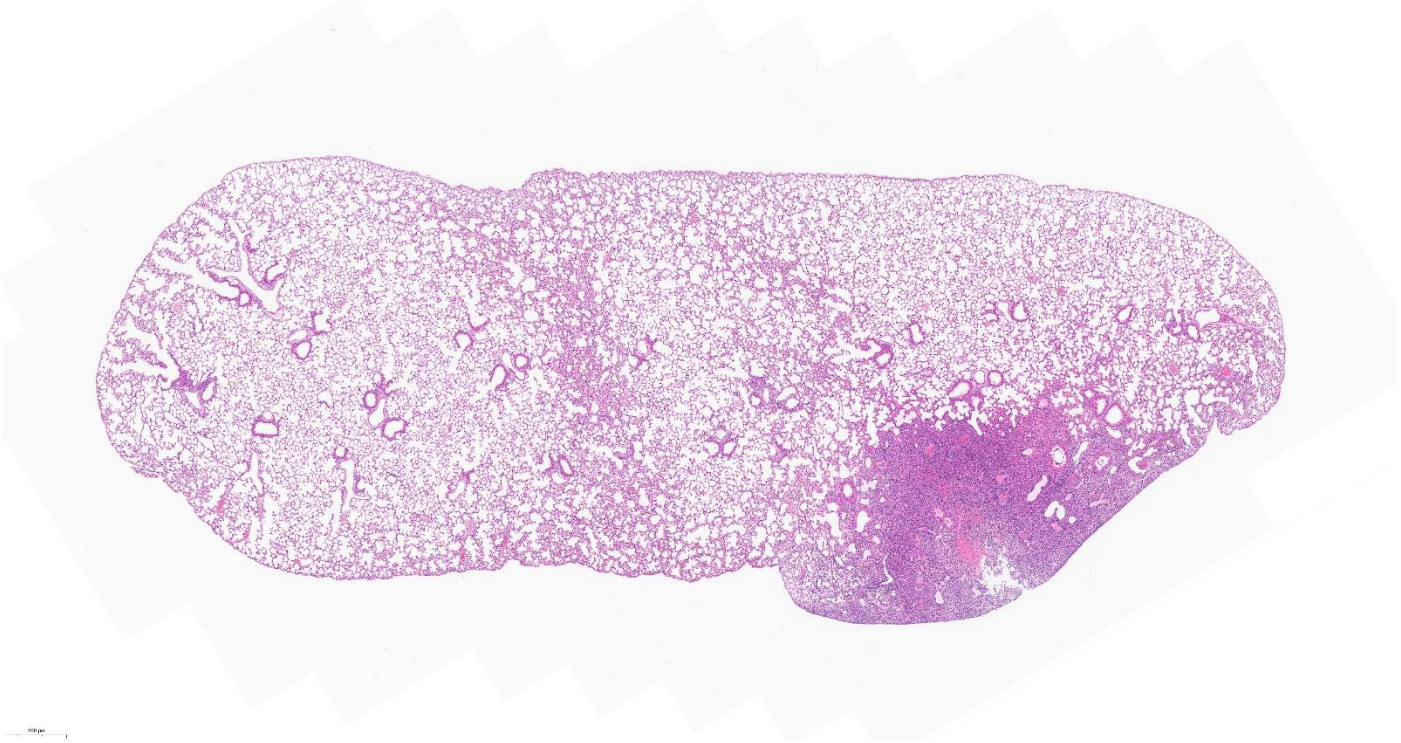

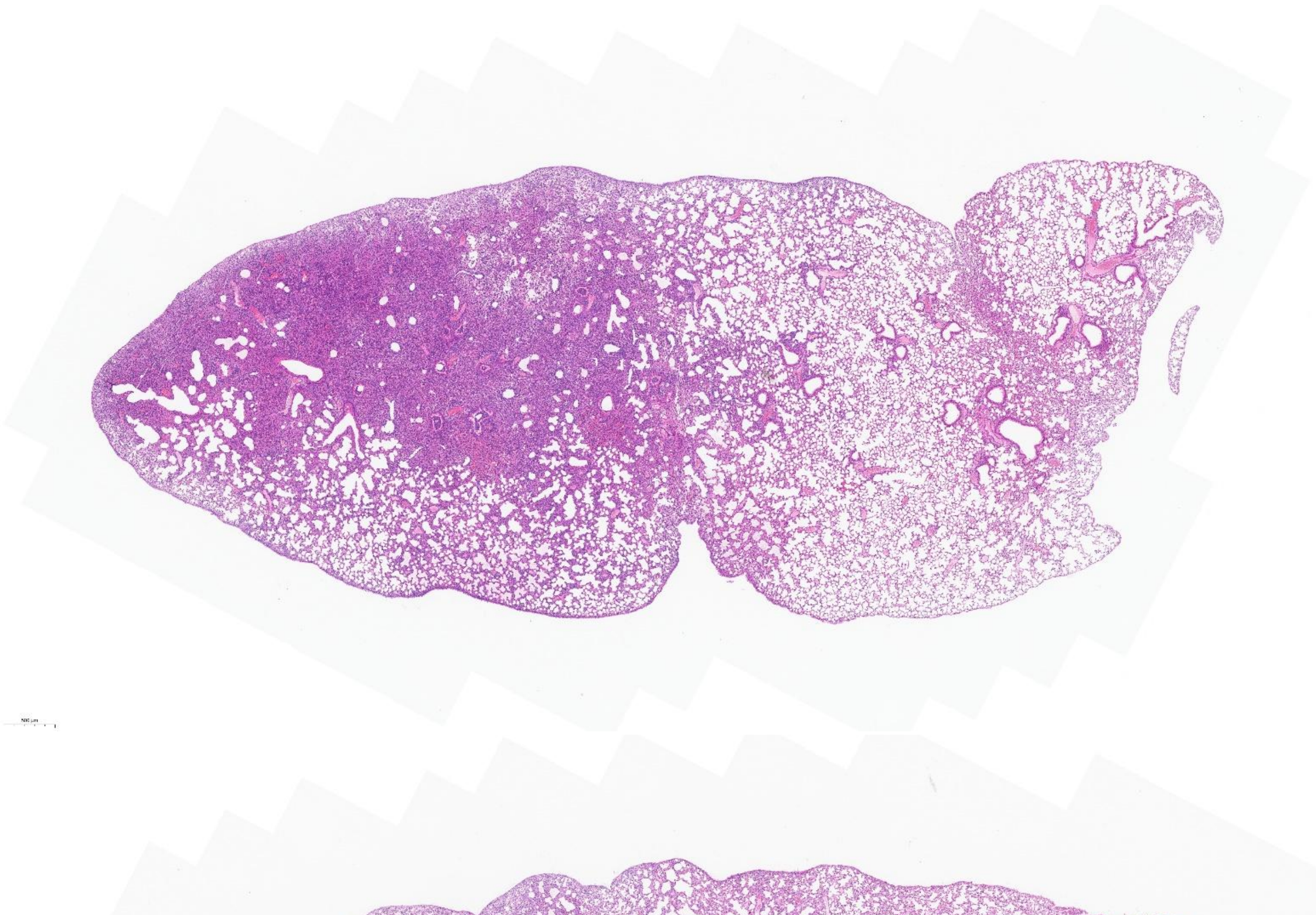

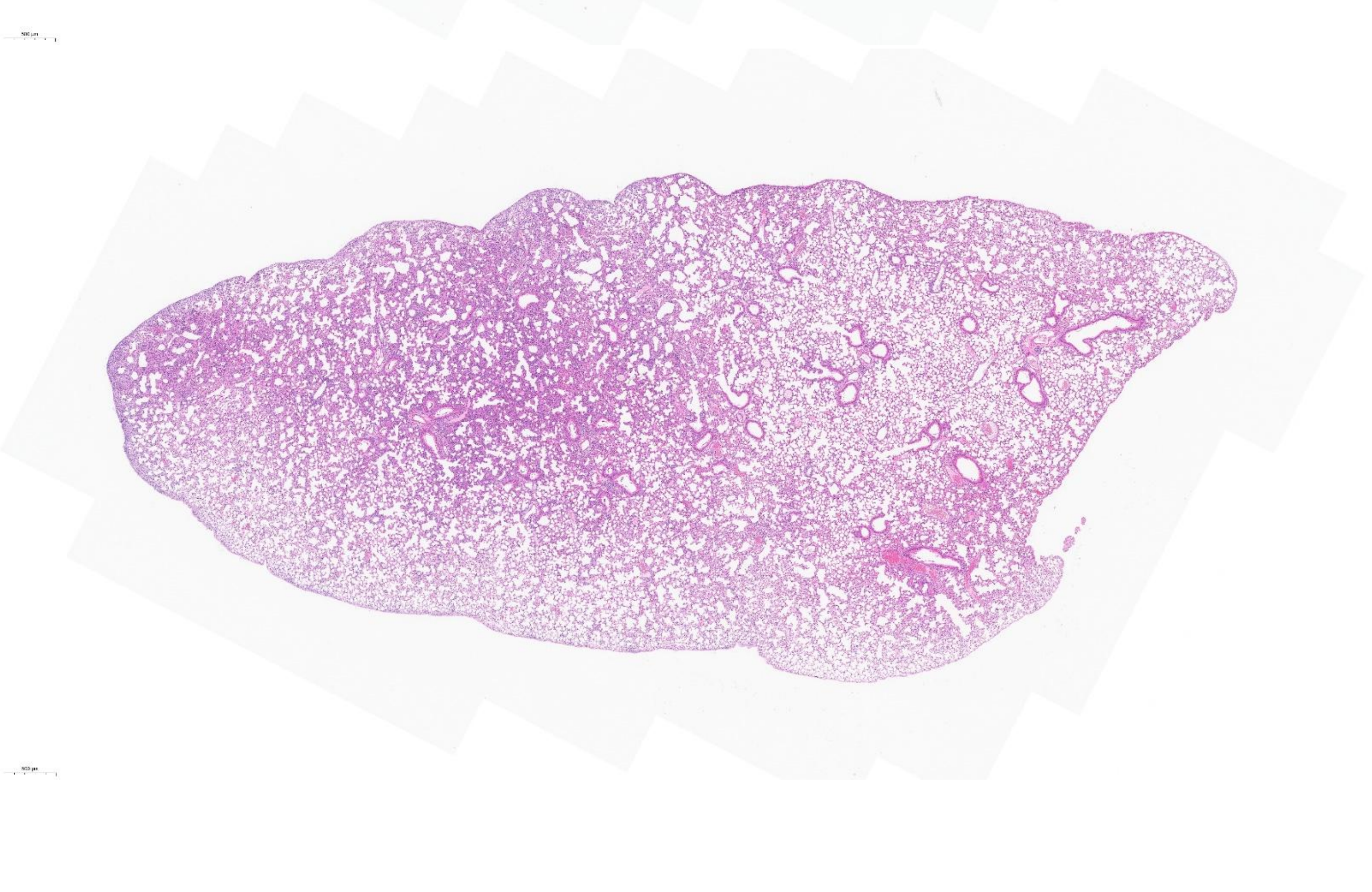

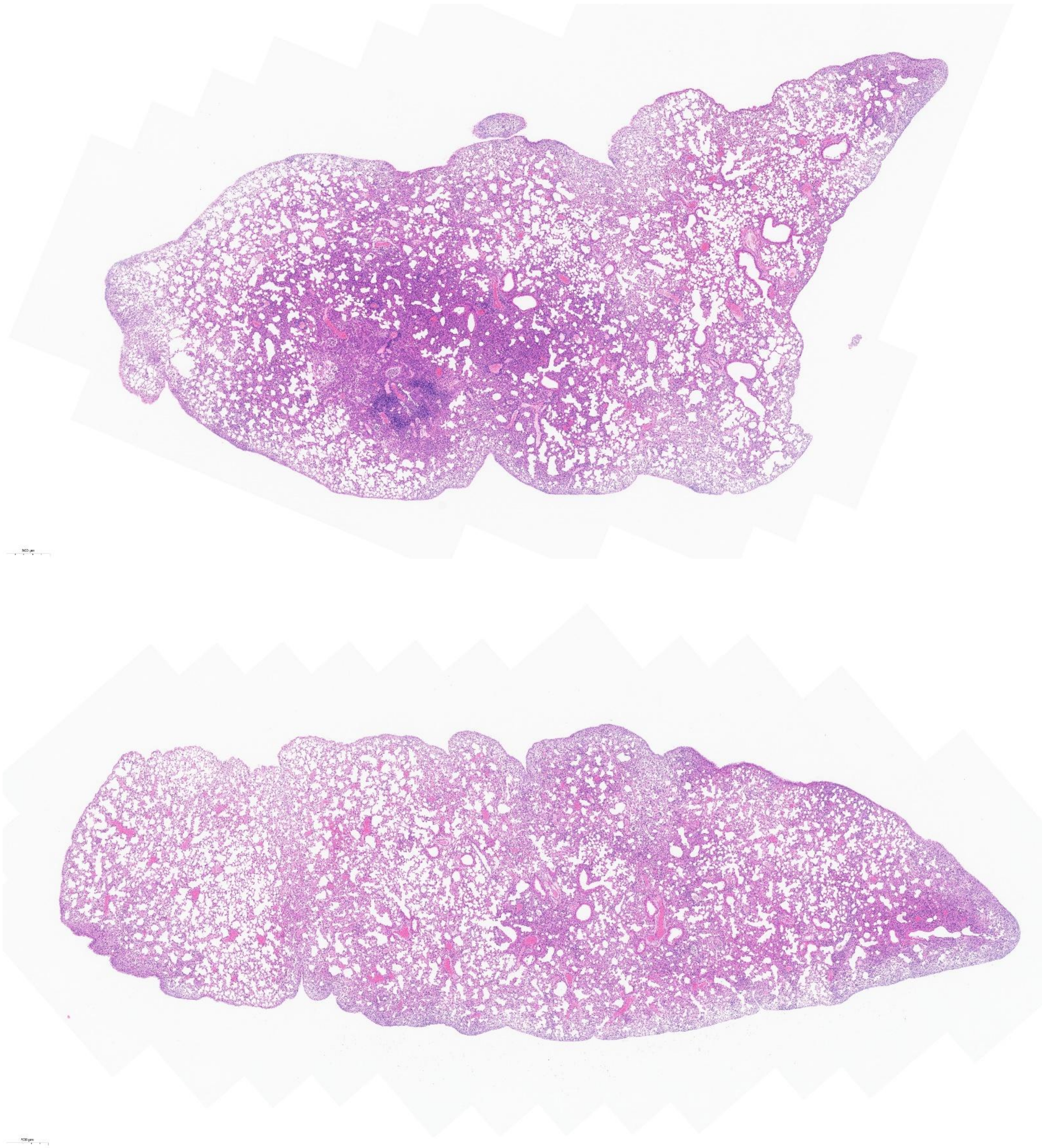

Supplementary Figure 4

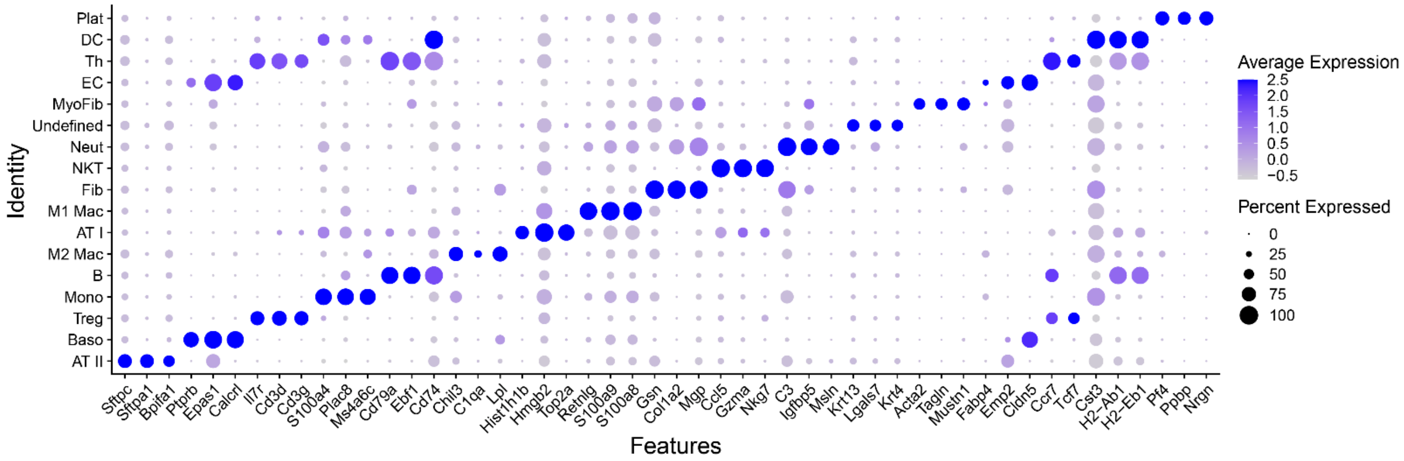

Supplementary Code

Figure 5:

```
##数据读入与整理：
library(GEOquery)
data <- getGEO(filename = "resource/GSE66890_series_matrix.txt",getGPL = F)
exprSet <- exprs(data)
exprSet <- as.data.frame(exprSet)
metadata <- pData(data)
save(exprSet,file = "exprSet.Rdata")
save(metadata,file = "metadata.Rdata")

##数据预处理
load(file = "output/exprSet.Rdata")
ex <- exprSet
qx <- as.numeric(quantile(ex, c(0., 0.25, 0.5, 0.75, 0.99, 1.0), na.rm=T))
LogC <- (qx[5] > 100) ||
  (qx[6]-qx[1] > 50 && qx[2] > 0) ||
  (qx[2] > 0 && qx[2] < 1 && qx[4] > 1 && qx[4] < 2)

if (LogC) {
  #solution 1
  ex[which(ex <= 0)] <- NaN
  ## 取 log2
  exprSet <- log2(ex)

  #solution 2
  # ex[which(ex <= 0)] <- 0
  # exprSet <- log2(ex+0.001)

  print("log2 transform finished")
  }else{
    print("log2 transform not needed")
  }

library(limma)
```

```
boxplot(exprSet,outline=FALSE, notch=T, las=2)
### 该函数默认使用 quntile 矫正差异
exprSet=normalizeBetweenArrays(exprSet)
boxplot(exprSet,outline=FALSE, notch=T, las=2)
## 这步把矩阵转换为数据框很重要
exprSet <- as.data.frame(exprSet)

################################02##################################
library(readxl)
probe2symbol_df <- read_excel("resource/probe_id.xls")
length(unique(probe2symbol_df$probe_id))
length(unique(probe2symbol_df$symbol))
####################################################################
library(dplyr)
library(tibble)
exprSet <- exprSet %>%
  ## 行名转列名,因为只有变成数据框的列,才可以用 inner_join
  rownames_to_column("probe_id") %>%
  ## 合并探针的信息
  inner_join(probe2symbol_df, by = "probe_id") %>%
  ## 去掉多余信息
  select(-probe_id) %>%
  ## 重新排列
  select(symbol, everything()) %>%
  ## rowMeans 求出行的平均数(这边的.代表上面传入的数据)
  ## .[,-1]表示去掉出入数据的第一列，然后求行的平均值
  mutate(rowMean = rowMeans(.[, -1])) %>%
  ## 把表达量的平均值按从大到小排序
  arrange(desc(rowMean)) %>%
  ## 去重，symbol 留下第一个
  distinct(symbol, .keep_all = TRUE) %>%
  ## 反向选择去除 rowMean 这一列
  select(-rowMean) %>%
  ## 检查并去除 symbol 列的缺失值
  filter(!is.na(symbol)) %>%
  ## 列转行名
  column_to_rownames("symbol")  ### 保存数据
save(exprSet,file = "output/exprSet_rmdup.Rdata")
```

###合并 GSE32707 与 GSE66890 数据集

```
library(AnnoProbe)
library(tidyverse)
library(tinyarray)
library(AnnoProbe)
library(sva)
library(FactoMineR)
library(factoextra)
library(limma)
library(dplyr)
```

```
########合并、去批次效应#########
load("resource/exprSet_rmdup2.Rdata")
eset_2 <- exprSet2
load("resource/exprSet_rmdup3.Rdata")
eset_3 <- exprSet

# 将多个表达矩阵的行名放入一个列表中
gene_lists <- list(rownames(eset_2), rownames(eset_3))
# 使用 Reduce 和 intersect 计算多个向量的交集
same_genes <- Reduce(intersect, gene_lists)
# 从合并后的数据
combine_data <- cbind(eset_2[same_genes,],eset_3[same_genes,])#提取共同基因所在的行并进行合并

##绘制去批次前图
#pdf(file = "去批次前所有样本合并图.pdf")
col <- c(rep("#FFD700", 47), rep("#3498DB", 28))#前六列样本显示蓝色，后面 16 列样本显示红色
boxplot(combine_data, col=col, outline=FALSE, notch=FALSE, las=2, xaxt="n")
#dev.off()

# 读取分组数据 ----------------------------------------------------------------
library(openxlsx)
group_2 <- read.xlsx("resource/group2.xlsx")
group_3 <- read.xlsx("resource/group3.xlsx")

#合并分组数据
group <- rbind(group_2,group_3)

# 正式开始去批次 --------------------------------------------------------------
GSE <- c(rep('GSE812',47),rep('GSE163',28))
GSE
group_list <- group$group
group_list
table(group_list,GSE)

data <- combine_data
batch <- c(rep('GSE812',47),rep('GSE163',28))
design <- model.matrix(~group_list)
#用 sva 包的 removeBatchEffect 去除批次效应

#存在缺失值
sum(is.na(data))
library(impute)
# 填补缺失值
data <- impute.knn(as.matrix(data))$data
save(data,file = "output/combine_data.Rdata")

expr_limma <- removeBatchEffect(data,batch = batch,design = design)
# 归一化数据
combine_after_data <- normalizeBetweenArrays(expr_limma)
```

```
save(combine_after_data,file = "output/combine_after_data.Rdata")
```

###8 种机器学习方法：

```
library(caret)
library(DALEX)
library(ggplot2)
library(randomForest)
library(kernlab)
library(xgboost)
library(pROC)
library(fs)
#设置种子，重复分析结果
set.seed(123)

#设置工作目录
# 读取文件
load("output/combine_after_data.Rdata")
expression_matrix<-combine_after_data
core_genes <- read.csv("resource/核心基因集.csv")[, 2]  # 提取第二列的基因名称
sample_classification<-read.csv("resource/样本信息.csv",row.names = 1)

##### 102 个基因
missing_genes <- setdiff(core_genes, rownames(expression_matrix))
print(missing_genes)
valid_genes <- intersect(core_genes, rownames(expression_matrix))
data <- expression_matrix[valid_genes, , drop = FALSE]
#提取核心基因的表达量：
#data <- expression_matrix[core_genes, , drop = FALSE]

# 去除表达为 NA 的值
data <- na.omit(data)
#转置数据
data <- t(data)

#增加分类信息
data <- as.data.frame(data)
data <- cbind(sample_classification[, 1], data)
colnames(data)[1] <- "Type"

#对数据进行分组
#p=0.7 指定了训练集占整个数据集的比例，这里设为 70%。
inTrain<-createDataPartition(y=data$Type, p=0.7, list=F)
train<-data[inTrain,]
test<-data[-inTrain,]

library(caret)
# 检查 Type 列的类别
train$Type <- as.factor(train$Type)
```

```
print(levels(train$Type))
# 检查列名
colnames(train) <- make.names(colnames(train))
# 设置 trainControl
control <- trainControl(
  method = "cv",
  number = 5,
  classProbs = TRUE,
  summaryFunction = twoClassSummary
)
# 检查数据结构
str(train)
train <- na.omit(train)
#########################测试数据###################
# 1. 同步修改列名为合法格式
colnames(test) <- make.names(colnames(test))
# 2. 确保测试数据包含与训练数据相同的列，并按相同顺序排列
test <- test[, colnames(train)]
# 3. 删除缺失值（如果训练数据中删除了缺失值）
test <- na.omit(test)

#RF 随机森林树模型
control=trainControl(method="repeatedcv", number=5, savePredictions=TRUE)
mod_rf = train(Type ~ ., data = train, method='rf',
               trControl = control)

#SVM 机器学习模型
mod_svm=train(Type ~., data = train, method = "svmRadial",
              prob.model=TRUE, trControl=control)

#GLM 模型
mod_glm=train(Type ~., data = train, method = "glm",
              family="binomial", trControl=control)

#GBM 模型
mod_gbm=train(Type ~., data = train, method = "gbm",
              trControl=control)
#KNN 模型
mod_knn=train(Type ~., data = train, method = "knn",
              trControl=control)

#NNET 模型
mod_nnet=train(Type ~., data = train, method = "nnet",
               trControl=control)

#Lsso 模型
mod_lasso=train(Type ~., data = train, method = "glmnet",
                trControl=control)
```

```
#DT 模型
mod_dt=train(Type ~., data = train, method = "rpart",
            trControl=control)

#定义预测函数
p_fun=function(object, newdata){
  predict(object, newdata=newdata, type="prob")[,2]
}
yTest=ifelse(test$Type=="Ctrl", 0, 1)##注意更改自己的分类类型

#RF 随机森林树模型预测结果
explainer_rf=explain(mod_rf, label = "RF",
                    data = test, y = yTest,
                    predict_function = p_fun,
                    verbose = FALSE)
mp_rf=model_performance(explainer_rf)
#SVM 机器学习模型预测结果
explainer_svm=explain(mod_svm, label = "SVM",
                     data = test, y = yTest,
                     predict_function = p_fun,
                     verbose = FALSE)
mp_svm=model_performance(explainer_svm)

#GLM 模型预测结果
explainer_glm=explain(mod_glm, label = "GLM",
                     data = test, y = yTest,
                     predict_function = p_fun,
                     verbose = FALSE)
mp_glm=model_performance(explainer_glm)

###GBM 模型预测结果
explainer_gbm=explain(mod_gbm, label = "GBM",
                     data = test, y = yTest,
                     predict_function = p_fun,
                     verbose = FALSE)
mp_gbm=model_performance(explainer_gbm)

#KKNN 模型预测结果
explainer_knn=explain(mod_knn, label = "KNN",
                      data = test, y = yTest,
                      predict_function = p_fun,
                      verbose = FALSE)
mp_knn=model_performance(explainer_knn)

#nnet 模型预测结果
explainer_nnet=explain(mod_nnet, label = "NNET",
                      data = test, y = yTest,
                      predict_function = p_fun,
                      verbose = FALSE)
```

```
mp_nnet=model_performance(explainer_nnet)

#lasso 模型预测结果
explainer_lasso=explain(mod_lasso, label = "LASSO",
                        data = test, y = yTest,
                        predict_function = p_fun,
                        verbose = FALSE)
mp_lasso=model_performance(explainer_lasso)

#DT 模型预测结果
explainer_dt=explain(mod_dt, label = "DT",
                     data = test, y = yTest,
                     predict_function = p_fun,
                     verbose = FALSE)
mp_dt=model_performance(explainer_dt)

#绘制四种方法的残差反向累计分布图
pdf(file="绝对残差图.pdf", width=6, height=6)
p1 <- plot(mp_rf, mp_svm, mp_glm, mp_gbm, mp_knn, mp_nnet,mp_lasso,mp_dt)
print(p1)
dev.off()

########## 自定义颜色向量（按模型标签命名）
custom_colors <- c(RF = "#FF4500", SVM = "#00FF00",GLM = "#0000FF",GBM = "#FF69B4",KNN = "#FFD700",NNET = "#00CED1",LASSO = "#9400D3",DT = "#FFA500")
# 通过 ggplot2 的 scale_color_manual 强制覆盖颜色
library(ggplot2)
p1_fixed <- p1 +
  scale_color_manual(values = custom_colors) +
  theme(legend.position = "bottom")
# 导出图形
pdf(file="绝对残差图-colour.pdf", width=6, height=6)
print(p1_fixed)
dev.off()

library(scales)
#绘制四种方法的残差箱线图
#pdf(file="箱形图.pdf", width=6, height=6)
p2 <- plot(mp_rf, mp_svm, mp_glm, mp_gbm, mp_knn , mp_nnet, mp_lasso, mp_dt,geom = "boxplot")
#print(p2)
#dev.off()
pdf("箱形图-colour.pdf", width=6, height=6)
p2 +
  scale_color_manual(values = custom_colors) +  # 修改线条/点颜色
  scale_fill_manual(values = alpha(custom_colors, 0.2)) + # 仅箱体填充透明度
  theme(legend.position = "bottom")
dev.off()

p3 <- plot(mp_rf, mp_svm, mp_glm, mp_gbm, mp_knn , mp_nnet, mp_lasso, mp_dt, geom = "histogram")
```

```
pdf("直方图.pdf", width=5, height=7)
print(
  p3 +
    scale_fill_manual(values = alpha(custom_colors, 1))  # 仅修改填充色
)
dev.off()

p4 <- plot(mp_rf, mp_svm, mp_glm, mp_gbm, mp_knn , mp_nnet, mp_lasso, mp_dt, geom = "prc")
p4 +
  scale_color_manual(values = custom_colors) +
  theme(legend.position = "right")  # 调整图例位置（避免文字重叠）
print(p4)

#绘制 ROC 曲线
pred1=predict(mod_rf, newdata=test, type="prob")
pred2=predict(mod_svm, newdata=test, type="prob")
pred3=predict(mod_glm, newdata=test, type="prob")
pred4=predict(mod_gbm, newdata=test, type="prob")
pred5=predict(mod_knn, newdata=test, type="prob")
pred6=predict(mod_nnet, newdata=test, type="prob")
pred7=predict(mod_lasso, newdata=test, type="prob")
pred8=predict(mod_dt, newdata=test, type="prob")

roc1=roc(yTest, as.numeric(pred1[,2]))
roc2=roc(yTest, as.numeric(pred2[,2]))
roc3=roc(yTest, as.numeric(pred3[,2]))
roc4=roc(yTest, as.numeric(pred4[,2]))
roc5=roc(yTest, as.numeric(pred5[,2]))
roc6=roc(yTest, as.numeric(pred6[,2]))
roc7=roc(yTest, as.numeric(pred7[,2]))
roc8=roc(yTest, as.numeric(pred8[,2]))

pdf(file="ROC.pdf", width=5, height=5)
plot(roc1, print.auc=F, legacy.axes=T, main="", col="#FF4500")
plot(roc2, print.auc=F, legacy.axes=T, main="", col="#00FF00", add=T)
plot(roc3, print.auc=F, legacy.axes=T, main="", col="#0000FF", add=T)
plot(roc4, print.auc=F, legacy.axes=T, main="", col="#FF69B4", add=T)
plot(roc5, print.auc=F, legacy.axes=T, main="", col="#FFD700", add=T)
plot(roc6, print.auc=F, legacy.axes=T, main="", col="#00CED1", add=T)
plot(roc7, print.auc=F, legacy.axes=T, main="", col="#9400D3", add=T)
plot(roc8, print.auc=F, legacy.axes=T, main="", col="#FFA500", add=T)

#plot(roc1, print.auc=F, legacy.axes=T, main="", col="chocolate")
#plot(roc2, print.auc=F, legacy.axes=T, main="", col="aquamarine3", add=T)
#plot(roc3, print.auc=F, legacy.axes=T, main="", col="bisque3", add=T)
#plot(roc4, print.auc=F, legacy.axes=T, main="", col="burlywood", add=T)
#plot(roc5, print.auc=F, legacy.axes=T, main="", col="darkgoldenrod3", add=T)
#plot(roc6, print.auc=F, legacy.axes=T, main="", col="darkolivegreen3", add=T)
#plot(roc7, print.auc=F, legacy.axes=T, main="", col="dodgerblue3", add=T)
```

```r
#plot(roc8, print.auc=F, legacy.axes=T, main="", col="darksalmon", add=T)

legend('bottomright',
       c(paste0('RF: ',sprintf("%.03f",roc1$auc)),
         paste0('SVM: ',sprintf("%.03f",roc2$auc)),
         paste0('GLM: ',sprintf("%.03f",roc3$auc)),
         paste0('GBM: ',sprintf("%.03f",roc4$auc)),
         paste0('KNN: ',sprintf("%.03f",roc5$auc)),
         paste0('NNET: ',sprintf("%.03f",roc6$auc)),
         paste0('LASSO: ',sprintf("%.03f",roc7$auc)),
         paste0('DT: ',sprintf("%.03f",roc8$auc))),


       col=c("#FF4500","#00FF00","#0000FF",
             "#FF69B4","#FFD700","#00CED1",
             "#9400D3","#FFA500"), lwd=2, bty = 'n')
dev.off()
```

Other GEO Data Preprocessing Methods:

```r
library(GEOquery)
data <- getGEO(filename = "resource/GSE69063_series_matrix.txt",getGPL = F)
exprSet <- exprs(data)
metadata <- pData(data)

save(metadata,file = "output/metadata.Rdata")
ex <- exprSet
qx <- as.numeric(quantile(ex, c(0., 0.25, 0.5, 0.75, 0.99, 1.0), na.rm=T))
LogC <- (qx[5] > 100) ||
  (qx[6]-qx[1] > 50 && qx[2] > 0) ||
  (qx[2] > 0 && qx[2] < 1 && qx[4] > 1 && qx[4] < 2)

## 开始判断
if (LogC) {
  #solution 1
  ex[which(ex <= 0)] <- NaN
  ## 取 log2
  exprSet <- log2(ex)

  #solution 2
  # ex[which(ex <= 0)] <- 0
  # exprSet <- log2(ex+0.001)

  print("log2 transform finished")
  }else{
    print("log2 transform not needed")
  }

library(limma)
boxplot(exprSet,outline=FALSE, notch=T, las=2)
```

```
### 该函数默认使用 quntile 矫正差异
exprSet=normalizeBetweenArrays(exprSet)
boxplot(exprSet,outline=FALSE, notch=T, las=2)
## 这步把矩阵转换为数据框很重要
exprSet <- as.data.frame(exprSet)

#################################################################
## 探针基因名转换
library(openxlsx)
probe2symbol_df <- read.xlsx("resource/probe.xlsx", sheet = 1)
class(probe2symbol_df)
length(unique(probe2symbol_df$probe_id))
length(unique(probe2symbol_df$symbol))

####################################################################
library(dplyr)
library(tibble)
exprSet <- exprSet %>%
  ## 行名转列名,因为只有变成数据框的列,才可以用 inner_join
  rownames_to_column("probe_id") %>%
  ## 合并探针的信息
  inner_join(probe2symbol_df,by="probe_id") %>%
  ## 去掉多余信息
  select(-probe_id) %>%
  ## 重新排列
  select(symbol,everything()) %>%
  ## rowMeans 求出行的平均数(这边的.代表上面传入的数据)
  ## .[,-1]表示去掉出入数据的第一列，然后求行的平均值
  mutate(rowMean =rowMeans(.[,-1])) %>%
  ## 把表达量的平均值按从大到小排序
  arrange(desc(rowMean)) %>%
  ## 去重，symbol 留下第一个
  distinct(symbol,.keep_all = T) %>%
  ## 反向选择去除 rowMean 这一列
  select(-rowMean) %>%
  ## 列转行名
  column_to_rownames("symbol")

### 本节任务：使用 limma 来做芯片的差异分析
#############
#加载 limma 包，用于校正和比较差异
rm(list = ls())
library(limma)
### 加载数据，注意解决报错
load(file = "output/exprSet_rmdup2.Rdata")
library(openxlsx)
metadata <- read.xlsx("output/metadata.xlsx", sheet = "Sheet1")
```

```
### 1.创建分组
group <- c(rep("ARDS", 8), rep("Ctrl", 11), rep("ARDS", 49))
#group <- c("con","con","treat","con","treat","treat")
### 分组变成向量，并且限定 leves 的顺序
### levels 里面，把对照组放在前面
group <- factor(group,levels = c("Ctrl","ARDS"))

### 主成分分析 PCA：提前预测结果
### 行是样本列是基因

res.pca <- prcomp(t(exprSet), scale = TRUE)
library(factoextra)
fviz_pca_ind(res.pca,col.ind = group)

### 构建比较矩阵
design <- model.matrix(~group)
### 比较矩阵命名
colnames(design) <- levels(group)
design

### 2.线性模型拟合
fit <- lmFit(exprSet,design)
### 3.贝叶斯检验
fit2 <- eBayes(fit)
### 4.输出差异分析结果,其中 coef 的数目不能操过 design 的列数
### 此处的 2 代表的是 design 中第二列和第一列的比较
allDiff=topTable(fit2,adjust='fdr',coef=2,number=Inf)
### 这个数据很重要需要保存一下
save(allDiff,file = "output/allDiff.Rdata")


library(dplyr)
diffgene <- allDiff %>%
  filter(adj.P.Val < 0.05) %>%
  filter(abs(logFC) >1)

save(diffgene,group,file = "output/diffgene.Rdata")
```

Figure 9:

```
input_file <- "exprSet.csv"
#input_file <- "Sample Type Matrix.csv"       # 定义混合表达数据文件路径

# 定义核心算法函数，用于对参考矩阵和目标向量进行 SVR 回归分析（尝试不同的 nu 值）
coreAlgorithm <- function(refer_matrix, target_vector) {  # 函数接收参考矩阵和目标向量
  num_steps <- 3                              # 定义尝试的步数（对应不同的 nu 值）
  runSVM <- function(step_index) {            # 内部函数，接收步长索引
    if (step_index == 1) {                    # 如果索引为 1
      nu_param <- 0.25                        # 设置 nu 参数为 0.25
```

```
    } else if (step_index == 2) {              # 如果索引为 2
      nu_param <- 0.5                        # 设置 nu 参数为 0.5
    } else if (step_index == 3) {              # 如果索引为 3
      nu_param <- 0.75                       # 设置 nu 参数为 0.75
    } else {                                 # 否则（冗余判断）
      nu_param <- 0.5                        # 默认设置 nu 参数为 0.5
    }
    # 训练 SVM 模型，使用线性核且不进行数据缩放
    svm_model <- svm(refer_matrix, target_vector, type="nu-regression", kernel="linear", nu=nu_param, 
scale=FALSE)
    return(svm_model)                        # 返回训练好的模型
  }  # 结束内部函数

  # 根据系统类型选择并行计算方式（Windows 系统不支持多核并行）
  if (Sys.info()['sysname'] == 'Windows') {     # 如果操作系统为 Windows
    models_list <- mclapply(1:num_steps, runSVM, mc.cores=1)  # 使用单核运行
  } else {                                   # 否则（Linux 或 Mac）
    models_list <- mclapply(1:num_steps, runSVM, mc.cores=num_steps)  # 使用多个核心并行计算
  }

  rmse_vec <- rep(0, num_steps)               # 初始化 RMSE 向量
  corr_vec <- rep(0, num_steps)               # 初始化相关系数向量
  for (i in 1:num_steps) {                    # 循环遍历每个模型
    current_model <- models_list[[i]]         # 获取当前模型
    weight_vals <- t(current_model$coefs) %*% current_model$SV
    weight_vals[which(weight_vals < 0)] <- 0    # 将负权重置为 0
    norm_weights <- weight_vals / sum(weight_vals)  # 归一化权重
    weighted_refer <- sweep(refer_matrix, MARGIN=2, norm_weights, '*')
    est_target <- apply(weighted_refer, 1, sum)
    rmse_vec[i] <- sqrt(mean((est_target - target_vector)^2))
    corr_vec[i] <- cor(est_target, target_vector)
  }
  best_idx <- which.min(rmse_vec)             # 获取 RMSE 最小模型的索引
  best_model <- models_list[[best_idx]]        # 选择最佳模型
  best_weights <- t(best_model$coefs) %*% best_model$SV
  best_weights[which(best_weights < 0)] <- 0     # 将负权重置为 0
  final_weights <- best_weights / sum(best_weights)  # 归一化最终权重

  # 存储最佳模型的 RMSE 和相关系数
  best_rmse <- rmse_vec[best_idx]             # 最佳 RMSE
  best_corr <- corr_vec[best_idx]             # 最佳相关系数

  # 将最终结果存入列表并返回
  result_list <- list("final_weights" = final_weights, "best_rmse" = best_rmse, "best_corr" = best_corr)
  return(result_list)                        # 返回结果列表
}  # 结束 coreAlgorithm 函数

# 定义置换检验函数，用于生成经验零分布
doPermutation <- function(num_perm, refer_matrix, mix_matrix) {  # 函数接收置换次数、参考矩阵和混合矩阵
```

```
  perm_counter <- 1                        # 初始化置换计数器
  target_list <- as.list(data.matrix(mix_matrix))  # 将混合矩阵转换为列表，方便采样
  corr_distribution <- numeric()             # 初始化存储相关系数的向量

  # 初始化置换进度条
  perm_progress <- txtProgressBar(min = 0, max = num_perm, style = 3)

  # 进行置换循环
  while (perm_counter <= num_perm) {          # 当置换计数器小于等于置换次数时
    # 随机采样混合数据（允许重复采样），生成新的目标向量
    permuted_target <- as.numeric(target_list[sample(length(target_list), nrow(refer_matrix), replace = TRUE)])
    # 判断标准差是否为 0，避免除零错误
    if (sd(permuted_target) == 0) {           # 如果标准差为 0
      std_target <- permuted_target          # 保持原样
    } else {
      std_target <- (permuted_target - mean(permuted_target)) / sd(permuted_target)  # 标准化目标向量
    }
    # 调用核心算法函数处理置换数据
    perm_result <- coreAlgorithm(refer_matrix, std_target)
    current_corr <- perm_result$best_corr     # 提取当前相关系数
    # 将当前相关系数追加到分布向量中
    corr_distribution <- c(corr_distribution, current_corr)
    # 更新进度条显示
    setTxtProgressBar(perm_progress, perm_counter)
    perm_counter <- perm_counter + 1         # 增加计数器
  }

  close(perm_progress)                        # 关闭置换进度条
  sorted_corr <- sort(corr_distribution)       # 将相关系数分布进行排序
  return(list("null_distribution" = sorted_corr))  # 返回经验零分布列表
}  # 结束 doPermutation 函数

# 定义主函数，用于执行 CIBERSORT 分析
CIBERSORTModified <- function(refer_file, mix_file, num_perm = 0, do_QN = TRUE) {  # 主函数接收参考文件、混合文件、置换次数和是否进行量化归一化的标志
  library(e1071)                           # 加载 e1071 包
  library(parallel)                         # 加载 parallel 包，用于并行计算
  library(preprocessCore)                   # 加载 preprocessCore 包，用于量化归一化

  # 检查参考文件是否存在
  if (!file.exists(refer_file)) {             # 如果参考文件不存在
    stop("参考文件未找到！")                   # 报错并停止程序
  }
  # 检查混合文件是否存在
  if (!file.exists(mix_file)) {              # 如果混合文件不存在
    stop("混合文件未找到！")                   # 报错并停止程序
  }

  # 读取参考矩阵数据（假设为制表符分隔）
```

```
refer_data <- read.table(refer_file, header = TRUE, sep = "\t", row.names = 1, check.names = FALSE)
# 读取混合矩阵数据（假设为逗号分隔）
mix_data <- read.table(mix_file, header = TRUE, sep = ",", row.names = 1, check.names = FALSE)
refer_data <- data.matrix(refer_data)
mix_data <- data.matrix(mix_data)
refer_data <- refer_data[order(rownames(refer_data)), ]
mix_data <- mix_data[order(rownames(mix_data)), ]
perm_count <- num_perm
if (max(mix_data) < 50) {
  mix_data <- 2^mix_data
}
if (do_QN == TRUE) {
  orig_colnames <- colnames(mix_data)      # 保存原始列名
  orig_rownames <- rownames(mix_data)      # 保存原始行名
  mix_data <- normalize.quantiles(mix_data)  # 进行量化归一化
  colnames(mix_data) <- orig_colnames       # 恢复列名
  rownames(mix_data) <- orig_rownames       # 恢复行名
}
refer_genes <- rownames(refer_data)
mix_genes <- rownames(mix_data)
common_genes <- intersect(refer_genes, mix_genes)  # 取交集
if (length(common_genes) == 0) {             # 如果没有共同基因，则终止程序
  stop("参考数据与混合数据之间没有共同基因！")
}

# 子集化参考矩阵和混合矩阵，只保留共同基因
refer_data <- refer_data[common_genes, , drop = FALSE]
mix_data <- mix_data[common_genes, , drop = FALSE]

# 对参考矩阵进行标准化（z-score 标准化）
refer_data <- (refer_data - mean(refer_data)) / sd(as.vector(refer_data))

# 如果置换次数大于 0，则计算经验零分布
null_dist <- NULL                        # 初始化空的零分布
if (perm_count > 0) {
  perm_results <- doPermutation(perm_count, refer_data, mix_data)
  null_dist <- perm_results$null_distribution  # 获取置换得到的零分布
}

# 定义输出表头
output_header <- c("Mixture", colnames(refer_data), "P-value", "Correlation", "RMSE")
output_matrix <- NULL                    # 初始化输出矩阵

# 初始化混合样本处理进度条
total_samples <- ncol(mix_data)          # 混合样本总数
sample_progress <- txtProgressBar(min = 0, max = total_samples, style = 3)

# 循环处理每个混合样本
for (sample_idx in 1:total_samples) {
```

```
    curr_sample <- mix_data[, sample_idx]   # 提取当前混合样本
    # 判断当前样本标准差是否为 0，避免除零错误
    if (sd(curr_sample) == 0) {
      std_sample <- curr_sample             # 如果标准差为 0，则保持原样
    } else {
      std_sample <- (curr_sample - mean(curr_sample)) / sd(curr_sample)  # 否则进行标准化
    }
    # 运行核心算法处理当前样本
    alg_result <- coreAlgorithm(refer_data, std_sample)
    weight_vec <- alg_result$final_weights  # 提取最终权重
    corr_val <- alg_result$best_corr          # 提取相关系数
    rmse_val <- alg_result$best_rmse          # 提取 RMSE 值

    # 计算经验 p 值（当置换结果可用时）
    p_val <- NA                             # 初始化 p 值为 NA
    if (!is.null(null_dist)) {              # 如果零分布存在
      p_val <- 1 - (which.min(abs(null_dist - corr_val)) / length(null_dist))  # 计算经验 p 值
    }

    # 整理当前样本结果，组合样本名、权重、p 值、相关系数和 RMSE
    curr_result <- c(colnames(mix_data)[sample_idx], weight_vec, p_val, corr_val, rmse_val)
    if (is.null(output_matrix)) {           # 如果输出矩阵为空（第一次循环）
      output_matrix <- curr_result          # 则初始化输出矩阵
    } else {                                # 否则
      output_matrix <- rbind(output_matrix, curr_result)  # 追加当前结果到输出矩阵
    }

    # 更新混合样本进度条
    setTxtProgressBar(sample_progress, sample_idx)
  }

  close(sample_progress)                    # 关闭样本处理进度条

  # 将输出表头与结果合并后写入 CSV 文件
  write.table(rbind(output_header, output_matrix), file = "CIBERSORT-Results.csv", sep = ",", row.names = FALSE,
col.names = FALSE, quote = FALSE)

  # 将结果转化为数值矩阵（去掉样本名称和表头），便于后续筛选
  result_numeric <- rbind(output_header, output_matrix)
  result_numeric <- result_numeric[, -1]    # 移除第一列（样本名称）
  result_numeric <- result_numeric[-1, ]    # 移除表头行
  result_numeric <- matrix(as.numeric(unlist(result_numeric)), nrow = nrow(output_matrix))
  rownames(result_numeric) <- colnames(mix_data)  # 设置行名为混合样本名
  colnames(result_numeric) <- c(colnames(refer_data), "P-value", "Correlation", "RMSE")  # 设置列名

  return(result_numeric)                    # 返回数值结果矩阵
}  # 结束 CIBERSORTModified 函数

# 运行修改后的 CIBERSORT 分析，并将结果存储到 result_table 中
```

```r
result_table <- CIBERSORTModified("小鼠的免疫特征矩阵文件.txt", input_file, num_perm = 1000, do_QN = TRUE)

filtered_table <- result_table[result_table[, "P-value"] < 0.05, ]
weight_only <- as.matrix(filtered_table[, 1:(ncol(filtered_table) - 3)])
final_output <- rbind(id = colnames(weight_only), weight_only)
write.table(final_output, file = "CIBERSORT-Results.csv", sep = ",", quote = FALSE, col.names = FALSE)
```

A

```r
library(reshape2)
library(ggplot2)
library(RColorBrewer)
library(ggpubr)
library(dplyr)
library(broom)

# ------------------ 数据准备 ------------------
setwd("H:\\常用分析生信\\48.免疫浸润可视化 1.热图")
inputFile <- "CIBERSORT-Results.csv"

rt <- read.table(inputFile, header = TRUE, sep = ",",
                 check.names = FALSE, row.names = 1)
rt$Group <- ifelse(grepl("Ctrl", rownames(rt), ignore.case = TRUE), "Ctrl",
                   ifelse(grepl("LPS", rownames(rt), ignore.case = TRUE), "LPS", NA))

control_samples <- rownames(rt)[rt$Group == "Ctrl"]
treat_samples   <- rownames(rt)[rt$Group == "LPS"]
all_samples_ordered <- c(control_samples, "gap", treat_samples)

rt$Sample <- rownames(rt)
data_long <- melt(rt, id.vars = c("Sample", "Group"),
                  variable.name = "Immune",
                  value.name = "Fraction")
data_long$Sample <- factor(data_long$Sample, levels = all_samples_ordered)
write.csv(data_long, file = "barplot_data_long.csv", row.names = FALSE)
cat("Barplot 长格式数据已保存为： barplot_data_long.csv\n")
immune_types <- unique(data_long$Immune)
nColors <- length(immune_types)
#myColors <- colorRampPalette(brewer.pal(13, "Set3"))(nColors)

base_colors <- c(
  RColorBrewer::brewer.pal(8, "Set1"),   # 8 种高对比色
  RColorBrewer::brewer.pal(8, "Dark2"),  # 另 8 种
  RColorBrewer::brewer.pal(9, "Pastel1") # 再补 9 种
)[1:nColors]
myColors <- sample(base_colors)  # 打乱顺序避免同类色聚集

# ------------------ 绘制堆叠条形图 ------------------
barplot_with_gap <- ggplot(data_long, aes(x = Sample, y = Fraction, fill = Immune)) +
  geom_bar(stat = "identity") +
```

```
  scale_fill_manual(values = myColors) +
  scale_x_discrete(drop = FALSE) +
  theme_minimal(base_size = 18) +
  theme(
    text = element_text(face = "bold"),
    axis.text.x  = element_blank(),
    axis.ticks.x = element_blank(),
    panel.grid.major.x = element_blank()
  ) +
  labs(
    x = NULL,
    y = "Relative Percent",
    fill = "Immune\nCell Type",
    title = "Immune Cell Distribution",
    subtitle = "Control vs. Treat "
  ) +
  coord_cartesian(clip = "off") +
  scale_y_continuous(expand = expansion(mult = c(0.1, 0.05)))
control_count <- length(control_samples)
treat_count   <- length(treat_samples)

barplot_with_gap_annot <- barplot_with_gap +
  # 添加 Control 组下方的线段和文字
  annotate("segment", x = 0.5, xend = control_count + 0.5,
           y = -0.04, yend = -0.04, color = "#D65DB1", size = 5) +
  annotate("text", x = (control_count)/2 + 0.5, y = -0.08,
           label = "Control", color = "#D65DB1", size = 7, fontface = "bold") +
  # 添加 Treat 组下方的线段和文字
  annotate("segment", x = control_count + 1.5,
           xend = control_count + treat_count + 1.5,
           y = -0.04, yend = -0.04, color = "#0089BA", size = 5) +
  annotate("text", x = control_count + (treat_count)/2 + 1.5, y = -0.08,
           label = "Treat", color = "#0089BA", size = 7, fontface = "bold")
barplot_with_gap_annot

ggsave("barplot_two_lines_set3_gap.pdf", barplot_with_gap_annot,
       width = 10, height = 6)
cat("堆叠条形图已保存为：barplot_two_lines_set3_gap.pdf\n")
```

B

```
library(reshape2)
library(ggplot2)
library(RColorBrewer)
library(ggpubr)
library(dplyr)
library(broom)
library(ggsci)
library(corrplot)
library(ggridges)
```

```
inputFile <- "CIBERSORT-Results.csv"

# 读取数据（假设每行代表一个样本，列为各免疫细胞的比例）
rt <- read.table(inputFile, header = TRUE, sep = ",",
                 check.names = FALSE, row.names = 1)

# 根据行名判断组别：后缀为 _con 的为 Control，后缀为 _tre 的为 Treat
rt$Group <- ifelse(grepl("Ctrl", rownames(rt), ignore.case = TRUE), "Ctrl",
                   ifelse(grepl("LPS", rownames(rt), ignore.case = TRUE), "LPS", NA))
# 添加样本名称列
rt$Sample <- rownames(rt)

# 获取 Control 与 Treat 的样本名，并构造样本顺序：Control -> gap -> Treat
control_samples <- rownames(rt)[rt$Group == "Ctrl"]
treat_samples   <- rownames(rt)[rt$Group == "LPS"]
all_samples_ordered <- c(control_samples, "gap", treat_samples)
data_long <- melt(rt, id.vars = c("Sample", "Group"),
                  variable.name = "Immune",
                  value.name = "Fraction")
# 指定因子水平，确保 gap 保留
data_long$Sample <- factor(data_long$Sample, levels = all_samples_ordered)

write.csv(data_long, file = "barplot_data_long.csv", row.names = FALSE)
cat("Barplot 长格式数据已保存为：barplot_data_long.csv\n")

countControl <- length(unique(data_long$Sample[data_long$Group == "Ctrl"]))
countTreat   <- length(unique(data_long$Sample[data_long$Group == "LPS"]))
myCuteColors <- c("Ctrl" = "#2E4052", "LPS" = "#D1495B")
boxplot_cute <- ggboxplot(
  data_long,
  x = "Immune",
  y = "Fraction",
  fill = "Group",
  palette = myCuteColors,
  xlab = "",
  ylab = "Fraction",
  legend.title = "Group",
  notch = FALSE,
  width = 0.8
) +
  stat_compare_means(
    aes(group = Group),
    label = "p.signif",
    symnum.args = list(
      cutpoints = c(0, 0.001, 0.01, 0.05, 1),
      symbols = c("***", "**", "*", "ns")
    )
  ) +
```

```r
  theme_classic(base_size = 14) +
  theme(
    legend.position = "top",
    axis.text.x = element_text(angle = 45, hjust = 1),
    axis.line = element_line(color = "black", size = 1),
    axis.ticks = element_line(color = "black", size = 1)
  ) +
  labs(
    title = "Immune Cell Comparison",
    subtitle = paste0(" (Ctrl n=", countControl, ", LPS n=", countTreat, ")")
  )

ggsave("immune_diff-points_n.pdf", boxplot_cute, width = 8, height = 6)
cat("箱线图已保存为：immune_diff-points_n.pdf\n")

summary_table <- data_long %>%
  filter(!is.na(Group)) %>%
  group_by(Immune, Group) %>%
  summarise(
    MeanFraction = mean(Fraction, na.rm = TRUE),
    MedianFraction = median(Fraction, na.rm = TRUE),
    SD = sd(Fraction, na.rm = TRUE),
    Count = n(),
    .groups = "drop"
  )
write.csv(summary_table, file = "boxplot_summary_table.csv", row.names = FALSE)
cat("箱线图统计汇总表已保存为：boxplot_summary_table.csv\n")

pvalue_table <- data_long %>%
  filter(!is.na(Group)) %>%
  group_by(Immune) %>%
  do(tidy(wilcox.test(Fraction ~ Group, data = .))) %>%
  select(Immune, p.value)
write.csv(pvalue_table, file = "immune_pvalues.csv", row.names = FALSE)
cat("免疫细胞在两组间的 p 值已保存为：immune_pvalues.csv\n")

boxplot_with_points <- ggboxplot(
  data_long,
  x = "Immune",
  y = "Fraction",
  fill = "Group",
  palette = myCuteColors,
  xlab = "",
  ylab = "Fraction",
  legend.title = "Group",
  notch = FALSE,
  width = 0.8
) +
  stat_compare_means(
```

```
    aes(group = Group),
    label = "p.signif",
    symnum.args = list(
      cutpoints = c(0, 0.001, 0.01, 0.05, 1),
      symbols = c("***", "**", "*", "ns")
    )
  ) +
  geom_jitter(shape = 21, color = "black", alpha = 0.7, width = 0.15, size = 2) +  # 添加样本点
  theme_classic(base_size = 14) +
  theme(
    legend.position = "top",
    axis.text.x = element_text(angle = 45, hjust = 1),
    axis.line = element_line(color = "black", size = 1),
    axis.ticks = element_line(color = "black", size = 1)
  ) +
  labs(
    title = "Immune Cell Comparison",
    subtitle = paste0(" (Ctrl n=", countControl, ", LPS n=", countTreat, ")")
  )

ggsave("immune_diff-points_n_with_samples.pdf", boxplot_with_points, width = 8, height = 6)
cat("带样本点的箱线图已保存为： immune_diff-points_n_with_samples.pdf\n")
```

C

```
library("limma")
library(dplyr)
library(tidyverse)
library(ggplot2)
#devtools::install_github("Hy4m/linkET")
library(linkET)
exprFilePath <- "exprSet.csv"        # 基因表达数据文件（CSV 格式）
targetGeneFile <- "geneList.txt"      # 待分析基因列表文件（txt 格式，使用制表符分隔）
immuneDataPath <- "CIBERSORT-Results.csv"

# 判断文件是否存在，若不存在则中止执行
if (!file.exists(exprFilePath)) {
  stop("错误：基因表达数据文件不存在！")
}
if (!file.exists(targetGeneFile)) {
  stop("错误：目标基因列表文件不存在！")
}
if (!file.exists(immuneDataPath)) {
  stop("错误：免疫细胞数据文件不存在！")
}

readExprData <- function(fileExpr, fileGene) {
  cat("步骤 2.1：读取基因表达数据...\n")  # 进度提示
  exprRaw <- read.table(fileExpr, header = TRUE, sep = ",", check.names = FALSE)  # 读取 CSV 数据
  exprMat <- as.matrix(exprRaw)                           # 转换为矩阵格式
```

```r
  rownames(exprMat) <- exprMat[, 1]                        # 第一列作为行名（基因名）
  exprVals <- exprMat[, -1, drop = FALSE]                  # 去除第一列，仅保留表达值

  # 设置行和列名，转换为数值矩阵
  rowNamesTmp <- rownames(exprVals)                        # 提取行名
  colNamesTmp <- colnames(exprVals)                        # 提取列名
  numExpr <- matrix(as.numeric(as.matrix(exprVals)),
                    nrow = nrow(exprVals),
                    dimnames = list(rowNamesTmp, colNamesTmp))  # 转换为数值型

  numExpr <- avereps(numExpr)  # 对重复探针做平均处理

  cat("步骤 2.2：读取目标基因文件...\n")  # 进度提示
  geneList <- read.table(fileGene, header = FALSE, sep = "\t", check.names = FALSE)  # 读取目标基因文件
  targetGene <- as.vector(geneList[, 1])     # 提取目标基因名称

  # 判断目标基因是否存在于表达矩阵中
  if (any(!(targetGene %in% rownames(numExpr)))) {
    stop("错误：某些目标基因未在表达数据中找到！")
  }

  # 筛选出目标基因数据，并进行转置（样本为行，基因为列）
  exprSelected <- numExpr[targetGene, , drop = FALSE]
  exprSelected <- t(exprSelected)

  # 返回一个列表，包含处理后的数据和目标基因名称
  return(list(exprData = exprSelected, geneName = targetGene))
}

# 定义函数：读取免疫细胞数据并与表达数据匹配
readImmuneData <- function(fileImm, exprData) {
  cat("步骤 2.3：读取免疫细胞数据...\n")  # 进度提示
  immData <- read.table(fileImm, header = TRUE, sep = ",", check.names = FALSE, row.names = 1)  # 读取免疫数据
  # 提取在表达数据和免疫数据中共有的样本
  commonSamples <- intersect(rownames(exprData), rownames(immData))

  # 判断是否存在共有样本
  if (length(commonSamples) == 0) {
    stop("错误：没有共同的样本在表达数据与免疫数据中！")
  }

  exprData <- exprData[commonSamples, , drop = FALSE]  # 仅保留共有样本的表达数据
  immData <- immData[commonSamples, , drop = FALSE]      # 仅保留共有样本的免疫数据

  # 去除标准差为 0 的列（免疫细胞类型），以防止相关性计算错误
  validImmune <- immData[, apply(immData, 2, sd) > 0, drop = FALSE]

  # 返回匹配后的表达数据和免疫数据
  return(list(exprData = exprData, immuneData = validImmune))
```

```
}

# 定义函数：计算多个目标基因与免疫细胞的 Spearman 相关性
computeCorrelation <- function(exprData, immuneData, genes) {
  cat("步骤 2.4：计算相关性...\n")  # 进度提示
  corrResults <- data.frame()   # 初始化存储相关性结果的数据框

  # 循环遍历每个目标基因
  for (gene in genes) {
    # 循环遍历每个免疫细胞类型
    for (cellType in colnames(immuneData)) {
      # 判断当前免疫细胞数据是否有足够的变异性
      if (sd(immuneData[, cellType]) == 0) {
        next  # 如果标准差为 0，则跳过
      }
      # 将免疫细胞数据和目标基因表达数据转为数值向量
      immuneVec <- as.numeric(immuneData[, cellType])
      geneExpr  <- as.numeric(exprData[, gene])
      # 执行 Spearman 相关性检验
      testResult <- cor.test(immuneVec, geneExpr, method = "spearman")

      # 将结果存入数据框中
      tempDF <- data.frame(spec = gene,               # 目标基因名称
                           env  = cellType,           # 免疫细胞类型
                           r    = as.numeric(testResult$estimate),  # 相关系数
                           p    = as.numeric(testResult$p.value))     # P 值
      corrResults <- rbind(corrResults, tempDF)  # 合并结果
    }
  }

  corrResults$pd <- ifelse(corrResults$p < 0.05,
                           ifelse(corrResults$r > 0, "positive correlation", "negative correlation"),
                           "not significant")

  # 将相关系数取绝对值，方便后续分档
  corrResults$r <- abs(corrResults$r)

  # 为相关系数添加分档信息
  corrResults <- corrResults %>%
    mutate(rd = cut(r,
                    breaks = c(-Inf, 0.2, 0.4, 0.6, Inf),
                    labels = c("< 0.2", "0.2 - 0.4", "0.4 - 0.6", ">= 0.6")))

  # 返回计算完成的相关性结果数据框
  return(corrResults)
}
totalSteps <- 5
pb <- txtProgressBar(min = 0, max = totalSteps, style = 3)  # 创建文本进度条
```

```
# 3.1 读取表达数据并筛选目标基因
setTxtProgressBar(pb, 1)          # 更新进度条
cat("主流程 3.1：处理基因表达数据...\n")  # 输出提示信息
exprOut <- readExprData(exprFilePath, targetGeneFile)  # 调用函数读取表达数据
exprMat <- exprOut$exprData          # 获取表达矩阵（样本×基因）
targetGene <- exprOut$gene           # 获取目标基因名称
Sys.sleep(0.2)                     # 冗余延时

# 3.2 读取免疫数据并匹配样本
setTxtProgressBar(pb, 2)          # 更新进度条
cat("主流程 3.2：处理免疫细胞数据...\n")
immuneOut <- readImmuneData(immuneDataPath, exprMat)   # 调用函数读取免疫数据
exprMat <- immuneOut$exprData       # 更新表达数据（匹配后的样本）
immuneMat <- immuneOut$immuneData  # 获取处理后的免疫数据
Sys.sleep(0.2)                     # 冗余延时

# 3.3 检查数据维度是否符合预期（冗余判断）
setTxtProgressBar(pb, 3)          # 更新进度条
if (nrow(exprMat) < 5 || nrow(immuneMat) < 5) {
  warning("样本数量较少，结果可能不稳定！")
}
cat("主流程 3.3：样本数检查完毕。\n")
Sys.sleep(0.2)

# 3.4 计算目标基因与各免疫细胞的相关性
setTxtProgressBar(pb, 4)          # 更新进度条
cat("主流程 3.4：计算相关性...\n")
corrDF <- computeCorrelation(exprMat, immuneMat, targetGene)  # 计算相关性
Sys.sleep(0.2)

# 3.5 将相关性结果输出到 CSV 文件中
setTxtProgressBar(pb, 5)          # 更新进度条
cat("主流程 3.5：写入相关性结果到 CSV 文件...\n")
write.csv(corrDF, file = "gene_correlation_results.csv", row.names = FALSE)  # 保存 CSV 文件
close(pb)                        # 关闭进度条



cat("步骤 4A：绘制图形风格 A...\n")  # 提示信息
plotA <- qcorrplot(correlate(immuneMat, method = "spearman"), type = "lower", diag = FALSE) +  # 绘制相关性矩阵
  geom_square(size = 1.0, colour = "#222222") +           # 每个单元格加边框
  geom_couple(aes(colour = pd, size = rd),                # 绘制耦合线（基因与免疫细胞之间的连线）
              data = corrDF, curvature = nice_curvature()) +
  scale_fill_gradientn(colours = rev(RColorBrewer::brewer.pal(11, "Spectral"))) +  # 设置填充色谱（反转 Spectral）
  scale_size_manual(values = c(0.5, 1, 2, 3)) +             # 自定义线宽
  scale_colour_manual(values = c("positive correlation" = "#FF1493",  # 设置正相关为亮粉
                                 "negative correlation" = "#00CED1",  # 负相关为亮青
                                 "not significant"  = "#999999")) +     # 非显著为灰色
  guides(size = guide_legend(title = "abs(Cor)", override.aes = list(colour = "grey35"), order = 2),
```

```
        colour = guide_legend(title = "P-value", override.aes = list(size = 3), order = 1),
        fill = guide_colorbar(title = "Cell-cell cor", order = 3)) +
  labs(x = "immune infiltrating cells", y = "immune infiltrating cells", title = "Gene-immune infiltrating cells
Correlation") +  # 设置坐标轴标签和标题
  theme_minimal(base_size = 14) +                      # 使用极简主题
  theme(axis.title.x = element_text(size = 14, face = "bold", color = "black"),  # 设置 X 轴标题格式
       axis.title.y = element_text(size = 14, face = "bold", color = "black"),  # 设置 Y 轴标题格式
       axis.text.x  = element_text(size = 12, face = "bold", color = "black", angle = 45, hjust = 1),  # X 轴文字旋转
       axis.text.y  = element_text(size = 12, face = "bold", color = "black"),  # Y 轴文字格式
       panel.grid.major = element_blank(),             # 去除主网格线
       panel.grid.minor = element_blank(),             # 去除次网格线
       plot.background  = element_rect(fill = "white", color = NA),  # 图形背景设置为白色
       plot.title       = element_text(size = 16, face = "bold", hjust = 0.5))  # 图标题居中加粗
plotA


plotA <- qcorrplot(correlate(immuneMat, method = "spearman"), type = "lower", diag = FALSE) +  # 绘制相关性矩
阵
  geom_square(size = 1.0, colour = "#222222") +          # 每个单元格加边框
  geom_couple(
    aes(
      colour = pd,
      size = rd,   # 保持原数据映射，但通过 scale_size 调整实际大小
      alpha = pd   # 新增透明度映射（按显著性分组）
    ),
    data = corrDF,
    curvature = nice_curvature(),
    show.legend = c(size = TRUE, alpha = FALSE) # 可选：隐藏 alpha 图例
  ) +
  scale_size_manual(values = c(0.3, 0.6, 1, 1.5)) +  # 减小线宽（原值：c(0.5,1,2,3)）
  scale_alpha_manual(values = c(0.6, 0.6, 0.3)) +    # 设置透明度（正/负相关 0.6，非显著 0.3）
  scale_fill_gradientn(colours = rev(RColorBrewer::brewer.pal(11, "Spectral"))) +  # 设置填充色谱（反转 Spectral）
  scale_colour_manual(values = c("positive correlation" = "#FF1493",   # 设置正相关为亮粉
                                 "negative correlation" = "#00CED1",   # 负相关为亮青
                                 "not significant"  = "#999999")) +     # 非显著为灰色
  guides(size = guide_legend(title = "abs(Cor)", override.aes = list(colour = "grey35"), order = 2),
        colour = guide_legend(title = "P-value", override.aes = list(size = 3), order = 1),
        fill = guide_colorbar(title = "Cell-cell cor", order = 3)) +
  labs(x = "immune infiltrating cells", y = "immune infiltrating cells", title = "Gene-immune infiltrating cells
Correlation") +  # 设置坐标轴标签和标题
  theme_minimal(base_size = 14) +                      # 使用极简主题
  theme(axis.title.x = element_text(size = 14, face = "bold", color = "black"),  # 设置 X 轴标题格式
       axis.title.y = element_text(size = 14, face = "bold", color = "black"),  # 设置 Y 轴标题格式
       axis.text.x  = element_text(size = 12, face = "bold", color = "black", angle = 45, hjust = 1),  # X 轴文字旋转
       axis.text.y  = element_text(size = 12, face = "bold", color = "black"),  # Y 轴文字格式
       panel.grid.major = element_blank(),             # 去除主网格线
       panel.grid.minor = element_blank(),             # 去除次网格线
       plot.background  = element_rect(fill = "white", color = NA),  # 图形背景设置为白色
       plot.title       = element_text(size = 16, face = "bold", hjust = 0.5))  # 图标题居中加粗
```

```
plotA


pdf(file = "cor_newStyle.pdf", width = 12, height = 7)  # 打开 PDF 设备
print(plotA)            # 打印图形到设备
dev.off()               # 关闭 PDF 设备

# 定义自定义主题函数（theme_cute），用于图形美化
theme_cute <- function() {
  theme_minimal() +  # 使用极简主题
    theme(
      plot.background  = element_rect(fill = "white", colour = NA),  # 图形背景白色
      panel.background = element_rect(fill = "white", colour = NA),  # 面板背景白色
      panel.grid.major = element_line(colour = "#f0f0f0", size = 0.5), # 主网格线设置
      panel.grid.minor = element_line(colour = "#f0f0f0", size = 0.25),# 次网格线设置
      axis.text        = element_text(size = 10, colour = "#555555"),   # 坐标轴文字设置
      axis.text.x      = element_text(angle = 90, hjust = 1, vjust = 0.5),# X 轴文字竖排
      axis.title       = element_text(size = 12, face = "bold", colour = "#555555"), # 坐标轴标题设置
      plot.title       = element_text(size = 18, face = "bold", colour = "#d35400", hjust = 0.5)  # 图标题设置
    )
}

plotB <- qcorrplot(correlate(immuneMat, method = "spearman"), type = "lower", diag = FALSE) +  # 绘制相关性矩阵
  geom_square() +                                   # 为每个单元格添加边框
  geom_couple(aes(colour = pd, size = rd), data = corrDF, curvature = nice_curvature()) +  # 添加耦合连线
  scale_fill_gradientn(
    colours = rev(RColorBrewer::brewer.pal(9, "Pastel2")),  # 使用 Pastel2 色系
    name = "Cell-Cell Correlation"                       # 色条名称
  ) +
  scale_size_manual(
    name   = "abs(Cor)",
    values = c("< 0.2" = 0.5, "0.2 - 0.4" = 1, "0.4 - 0.6" = 2, ">= 0.6" = 3),
    labels = c("< 0.2" = "< 0.2", "0.2 - 0.4" = "0.2 - 0.4", "0.4 - 0.6" = "0.4 - 0.6", ">= 0.6" = "⩾ 0.6")
  ) +
  scale_colour_manual(
    name   = "p-value",
    values = c("positive correlation" = "#F28C8C",   # 正相关色：亮粉
               "negative correlation" = "#8AB8FF",   # 负相关色：亮蓝
               "not significant"      = "#B2BABB"),  # 非显著色：灰蓝
    labels = c("positive correlation" = "Positive",
               "negative correlation" = "Negative",
               "not significant"      = "Not significant")
  ) +
  guides(
    size   = guide_legend(title = "abs(Cor)", override.aes = list(colour = "grey35"), order = 2),
    colour = guide_legend(title = "p-value", override.aes = list(size = 3), order = 1),
    fill   = guide_colorbar(title = "Cell-Cell Correlation", order = 3)
  ) +
```

```
  ggtitle("Single Gene–immune infiltrating cells Correlation") +  # 设置图形标题
  theme_cute() +             # 应用自定义主题
  labs(x = NULL, y = NULL)   # 删除 X/Y 轴标题
```

Figure 11:

```
rm(list = ls())
library(Seurat)
library(harmony)
library(SeuratWrappers)

### 1.读入数据
data1 <- readRDS(file = "output/NC1_seurat.rds")
data2 <- readRDS(file = "output/NC2_seurat.rds")
data3 <- readRDS(file = "output/NC3_seurat.rds")
data4 <- readRDS(file = "output/LPS1_seurat.rds")
data5 <- readRDS(file = "output/LPS2_seurat.rds")
data6 <- readRDS(file = "output/LPS3_seurat.rds")
data <- list(data1,data2,data3,data4,data5,data6)

files = list.files(path = "output/",pattern = "*_seurat.rds")
data <- lapply(files,function(i){readRDS(paste0("output/",i))})

### 2.Merge 合并数据
scobj <- merge(x=data[[1]], y = data[-1])

rm(list =  ls(pattern="data.*"))
### 3.数据质控
scobj[["percent.mt"]] <- PercentageFeatureSet(scobj, pattern = "^MT-")
#scobj@meta.data$percent.mt <- PercentageFeatureSet(scobj, pattern = "^MT-")

metadata <- scobj@meta.data

VlnPlot(scobj, features = c("nFeature_RNA", "nCount_RNA", "percent.mt"), ncol = 3)
scobj <- subset(scobj, subset = nFeature_RNA > 200 & nFeature_RNA < 4500 & percent.mt < 5)

scobj <- NormalizeData(scobj)
scobj <- FindVariableFeatures(scobj, selection.method = "vst", nfeatures = 2000)
scobj <- ScaleData(scobj, features = rownames(scobj))
scobj <- RunPCA(scobj, features = VariableFeatures(object = scobj),reduction.name = "pca")
library(Matrix)
library(RSpectra)
library(irlba)

scobj <- RunHarmony(scobj,reduction = "pca",group.by.vars = "group",reduction.save = "harmony")
scobj <- RunUMAP(scobj, reduction = "harmony", dims = 1:30,reduction.name = "umap")

p1 <- DimPlot(scobj, reduction = "umap_naive",group.by = "group")
p2 <- DimPlot(scobj, reduction = "umap",group.by = "group")
p1+p2
```

```
scobj <- FindNeighbors(scobj, reduction = "harmony", dims = 1:30)
scobj <- FindClusters(scobj, resolution = seq(0.2,1,0.1))

metadata <- scobj@meta.data
library(clustree)
clustree(scobj)

resolutions = seq(0.2,1,0.1)
DimPlot(scobj, reduction = "umap",
        group.by = paste0("RNA_snn_res.", resolutions),
        ncol = 3, label = T) & NoLegend()

### 选定分辨率 0.4 作为分群
Idents(scobj) <- scobj@meta.data$RNA_snn_res.0.4

DimPlot(scobj, reduction = "umap", label = T)
DimPlot(scobj, reduction = "umap", group.by = "group")
DimPlot(scobj, reduction = "umap", split.by = "group")

scobj@assays$RNA@scale.data <- matrix()

names(scobj@reductions)
FeaturePlot(scobj, features = "MS4A1", order = TRUE,reduction = "umap")
scobj@reductions$umap_naive <- NULL
FeaturePlot(scobj, features = "MS4A1", order = TRUE)
saveRDS(scobj,file = "output/hamony_seurat_unannotaion_20250211.rds")


rm(list = ls())
library(Seurat)
scobj <- readRDS(file = "output/hamony_seurat_unannotaion_20250211.rds")
# 绘制 UMAP 图，使用有序的细胞群标签
DimPlot(scobj, reduction = "umap", label = T)
### 汇总画图
marker_genes <- c("Mki67","Cd79a",
                  "Sftpc", "Sftpb", "Ager","Aqp5",
                  "Cd79a","Ms4a1","Cd19","Pax5",
                  "Mcpt4","Cpa3","Kit","Cd200r3","Fcer1a",
                  "Cd209a","Itgax",
                  "Pecam1","Vwf","Fli1","Cdh5","Cldn5",
                  "Col1a1","Dcn","Pdgfra","Tcf21","Pdpn",
                  "Cd68","Cd86","Nos2","Ccl7","Mgl2",
                  "Mrc1","Arg1","Chil3","Marco",
                  "Cd14","Itgam","F13a1","Treml4",
                  "Acta2", "Myh11", "Tagln", "Aspn", "Tagln",
                  "Cd3e","Klrb1c","Ncr1","Nkg7",
                  "Ly6g","S100a8","Retnlg",
```

```
                  "Pdgfrb","Cspg4","Rgs5","Gucy1a1","Vtn","Higd1b",
                  "Foxp3","Cd4","Il2ra",
                  "Cd4","Il17a","Ifng",
                  "Ppbp"
)
VlnPlot(scobj, features = marker_genes,ncol=10)
FeaturePlot(scobj, features = marker_genes, order = TRUE,ncol=4)

all_markers <- FindAllMarkers(object = scobj)
saveRDS(all_markers,file = "output/Seurat_stim_all_markers.rds")
write.csv(all_markers,"output/all_markers.csv")
all_markers <- readRDS(file = "output/Seurat_stim_all_markers.rds")
library(dplyr)
top_markers <- all_markers %>%
  group_by(cluster) %>%
  arrange(desc(avg_log2FC))%>%
  slice(1:15) %>%
  ungroup()
saveRDS(top_markers,file = "output/Seurat_stim_top_markers.rds")
write.csv(top_markers,"output/top_markers.csv")
library(Nebulosa)
marker_genes <- c("CCR7","SELL","CREM","CD69")
marker_genes <- c("CD8A")
VlnPlot(scobj, features = marker_genes)
plot_density(scobj,features = marker_genes) + plot_layout(ncol = 2)

### old umap
# scobj1 <- readRDS("output/old/hamony_seurat_unannotaion.rds")
# DimPlot(scobj1,label = T)

### A.确认群的个数
head(Idents(scobj))
Idents(scobj) <- scobj@meta.data$RNA_snn_res.0.4
DimPlot(scobj,label = T)


current_labels <- levels(scobj@meta.data$RNA_snn_res.0.4)
new_labels <- sort(as.numeric(current_labels))
scobj@meta.data$sorted_clusters <- factor(scobj@meta.data$RNA_snn_res.0.4, levels = current_labels, labels = new_labels)
DimPlot(scobj, reduction = "umap", group.by = "sorted_clusters", label = T)


### B.给每个群添加注释
scobj <- RenameIdents(scobj,
                      "0"="B",
                      "1"="Treg",
                      "2"="M1 Mac",
                      "3"= "Mono",
```

```
                "4"= "NKT",
                "5"= "Mono",
                "6"= "M2 Mac",
                "7"= "Fib",
                "8"= "Treg",
                "9"= "Neut",
                "10"= "M1 Mac",
                "11"= "Baso",
                "12"= "M2 Mac",
                "13"= "DC",
                "14"="Undefined",
                "15"="AT I",
                "16"="AT II",
                "17"="AT I",
                "18"="Undefined",
                "19"="EC",
                "20"="AT II",
                "21"="Mono",
                "22"="MyoFib",
                "23"="EC",
                "24"="AT I",
                "25"="Plat",
                "26"="MyoFib",
                "27"="AT II",
                "28"="Mono",
                "29"="Th",
                "30"="Mono",
                "31"="Undefined",
                "32"="NKT",
                "33"="M2 Mac",
                "34"="Neut"
)
head(Idents(scobj))
DimPlot(scobj, reduction = "umap", label = T)
metadata <- scobj@meta.data
scobj@meta.data$celltype = Idents(scobj)

Idents(scobj) <- scobj@meta.data$celltype
saveRDS(scobj,file = "output/hamony_seurat_annotaion_20250211.rds")


###把多组的细胞总数矫正
rm(list = ls())
scobj <- readRDS(file = 'output/hamony_seurat_annotaion_20250211.rds')
DimPlot(scobj,label = T)
DimPlot(scobj,label = T,split.by = "group")
objectList <- SplitObject(scobj,split.by = "group")
scobj_LPS = objectList[["LPS"]]
scobj_NC = objectList[["NC"]]
```

```
cellsTokeep = sample(rownames(scobj_LPS@meta.data),5000)
scobj_LPS_LPS = subset(scobj,cells = cellsTokeep)
p1 = DimPlot(scobj_LPS_LPS,label = T)
p2 = DimPlot(scobj_LPS,label = T)
p1+p2

### 合并数据
data1 <- scobj_LPS_LPS
data2 <- objectList[["NC"]]
data <- list(data1,data2)
scobj <- merge(x=data[[1]], y = data[-1])
scobj <- NormalizeData(scobj)
scobj <- FindVariableFeatures(scobj, selection.method = "vst", nfeatures = 2000)
scobj <- ScaleData(scobj, features = rownames(scobj))
scobj <- RunPCA(scobj, features = VariableFeatures(object = scobj),reduction.name = "pca")
library(harmony)
scobj <- RunHarmony(scobj,reduction = "pca",group.by.vars = "group",reduction.save = "harmony")
Reductions(scobj)
library(Matrix)
library(RSpectra)
library(irlba)
scobj <- RunUMAP(scobj, reduction = "harmony", dims = 1:30,reduction.name = "umap")

DimPlot(scobj,reduction = 'umap',label = T,
        group.by = "celltype",split.by = "group")

FeaturePlot(scobj,features = "POLR1B",split.by = "group",pt.size = 1.2)

### 统一细胞总数
table(scobj[["group"]])
minsize = min(table(scobj[["group"]]))
cellTokeep = do.call("c",lapply(SplitObject(scobj,split.by = "group"), function(i){
  sample(rownames(i@meta.data),size = 5000,replace = F)
}))

scobj_new = subset(scobj,cells = cellTokeep)
table(scobj_new[["group"]])
FeaturePlot(scobj_new,features = "IGF2BP3",split.by = "group",pt.size = 1.2)

scobj_new@assays$RNA@scale.data <- matrix()
saveRDS(scobj_new,file = "output/samecell_seurat_annotaion_20250211.rds")



###绘图
rm(list = ls())
library(Seurat)
scobj <- readRDS(file = "output/samecell_seurat_annotaion_20250211.rds")
top_markers <- c("Ptpn6","Isg20","Myo1f")
```

```
library(Nebulosa)
plot_density(scobj,features = top_markers) + plot_layout(ncol = 1)
top_markers <- c("Ptpn6","Isg20","Myo1f")#失眠

DotPlot(scobj, features = top_markers)
DotPlot(scobj, features = top_markers) + RotatedAxis()
DotPlot(scobj, features = top_markers) + coord_flip()+ RotatedAxis()
DotPlot(scobj, features = top_markers,dot.scale = 4) + coord_flip()+ RotatedAxis()+
theme(axis.text.y = element_text(size = 6))

scCustomize::Clustered_DotPlot(scobj, features = top_markers)
```
